# Perception and neural representation of intermittent odor stimuli in mice

**DOI:** 10.1038/s41467-026-72445-1

**Published:** 2026-04-29

**Authors:** Luis E. Boero, Hao Wu, Joseph D. Zak, Paul Masset, Farhad Pashakhanloo, Siddharth Jayakumar, Bahareh Tolooshams, Demba Ba, Venkatesh N. Murthy

**Affiliations:** 1 Department of Molecular & Cellular Biology, Harvard University, Cambridge, MA USA; 2 Center for Brain Science, Harvard University, Cambridge, MA USA; 3 Department of Chemistry & Chemical Biology, Harvard University, Cambridge, MA USA; 4 Department of Biological Sciences, University of Illinois, Chicago, IL USA; 5 Department of Psychology, McGill University, Montreal, QC Canada; 6 Mila - Quebec Artificial Intelligence Institute, Montreal, Canada; 7 Alberta Machine Intelligence Institute (Amii), Edmonton, AB Canada; 8 Neuroscience and Mental Health Institute (NMHI), University of Alberta, Edmonton, AB Canada; 9 John A. Paulson School of Engineering and Applied Sciences, Harvard University, Cambridge, MA USA; 10 Kempner Institute for the Study of Natural & Artificial Intelligence, Harvard University, Cambridge, MA USA

**Keywords:** Neuroscience, Olfactory system

## Abstract

Odor cues in nature are sparse and fluctuating due to turbulent transport. To investigate how animals perceive these intermittent cues, we developed a behavioral task in which mice made binary decisions based on the total number of discrete odor pulses presented stochastically over several seconds. Mice quickly learned this task, placing higher perceptual weight to stimuli arriving during inhalation than exhalation, a phase dependency that strongly correlated with the magnitude of responses in olfactory sensory neurons. Neurons in the anterior piriform cortex responded to odor pulses with varying degrees of dependence on respiration phase. Single cortical neurons responded stochastically and transiently to odor pulses, leading to a representation that carries signatures of sensory evidence, but not its accumulation. Our study reveals that mice can integrate intermittent odor signals across dozens of breaths and that respiratory modulation imposes limits on sensory information acquisition that cortical circuits cannot overcome to improve behavior.

## Abstract (web summary)

Animals navigate odor plumes by exploiting dynamic and intermittent cues. Here, the authors show that mice can discriminate among different fluctuating odor signals, and characterize their neural encoding in the olfactory system and its modulation by breathing.

## Introduction

Many animals can navigate fluctuating odor cues to locate food or mates^1–3^. Continuous odor gradients may be accessible for microscopic organisms, but they are rarely experienced by larger animals due to the action of turbulent fluid flow^4,5^. Instead, animals navigating airborne odor plumes may go through a spatiotemporally discontinuous olfactory landscape, characterized by short pulses of detectable odor concentrations (‘whiffs’) interspersed with time-varying blanks^4,6–8^. In this sparse regime, it is unclear what features of olfactory stimuli animals extract to gain information about odor sources. Physical simulations have revealed how environmental factors can affect olfactory statistics at different locations within and outside the plume. In particular, whiffs and blanks frequencies and duration, as well as odor intermittency - a related quantity measuring the probability of odor signals being above a detection threshold - were found to depend on the animal’s position within the plume^8–10^. The spatiotemporal integration of those statistical features could allow animals to make informed decisions during odor-guided navigation, similar to the accumulation of auditory and visual information for prey localization during hunting behaviors^11,12^.

In mice, like most other mammals, snapshots of the olfactory scene are acquired by discrete sampling events contingent on the influx of air to the nasal cavity. This is exactly what happens during normal respiration, but mice can also actively control sampling further by modulating sniffing depth and frequency^13–15^. Once in the nasal cavity, odor molecules activate olfactory sensory neurons (OSNs) in the olfactory epithelium^16^. Inputs from different types of OSNs converge on distinct glomeruli in the olfactory bulb (OB)^17^, where the information is processed and transmitted to mitral and tufted cells (MTCs) that ultimately relay it to multiple areas in the olfactory cortex, hippocampus, and amygdala^18^. The anterior piriform cortex (APCx) is one of the main targets of MTCs, plays a significant role in the representation of odor identities, and it has also extensive connections with other olfactory and non-olfactory areas^19^.

Extensive prior work has characterized how sniffing modulates the basal activity and the time course of neuronal responses in the olfactory pathway^20–27^. However, those studies tended to use continuous odor stimuli extending over multiple sniffs or very brief optogenetic activations. Recent work in mice has begun to characterize the representation of fluctuating olfactory stimuli and shown that mice can discriminate variations in the frequency and phase of trains of brief, square odor pulses delivered at frequencies up to 40 Hz, probably leveraging on the high temporal fidelity of OSNs and MTCs^28,29^. Moreover, when mice were presented with more naturalistic odor plumes, neural responses in the OB were able to follow their temporal dynamics^30,31^, with different glomeruli tuned to different intermittency levels^32^. Nevertheless, little is known about how fast, fluctuating stimuli such as the ones found in naturalistic odor plumes are encoded and integrated across the olfactory pathway, how odor sampling interacts with this process, and whether their representation is useful for animals to make sense of the olfactory landscape.

Here, we show that trained mice can discriminate odor stimuli with different total pulse counts, and that their decisions can be modeled well using signal detection theory. Odor pulses falling on distinct phases of breathing were perceived differently by mice, and this phase-dependence was remarkably similar to sensory responses in the OSNs. APCx neural responses to odor pulses were also modulated by breathing phase, but were stochastic and transient, which hindered the accumulation of evidence. Therefore, our results provide new insights about the encoding of sub-sniff, fluctuating olfactory stimuli, while opening new questions regarding how olfactory evidence accumulation can underlie odor-guided navigation.

## Results

### Odor pulse counting task

**Fig. 1:**
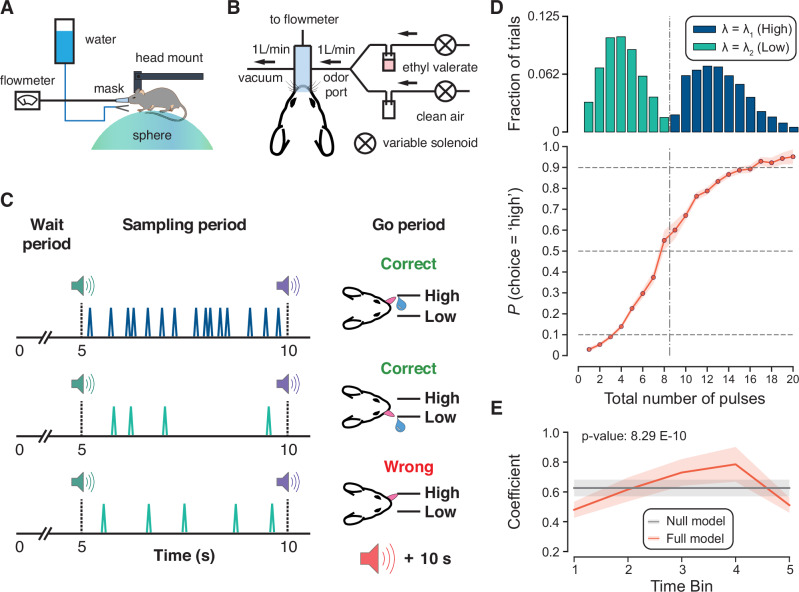
Mice can discriminate olfactory stimuli with different temporal statistics. **A** Side view of the behavioral rig. Head-restrained mice were placed over a sphere, and a mask was fitted to their snout for odor delivery. **B** Schematic of the olfactometer and connections to the mask. Output of the olfactometer arrives directly to the mask, which is also connected to a vacuum line, and a flow sensor for breathing monitoring. **C** Schematic of the behavioral task and three example trials with their corresponding outcomes given the number of pulses delivered. **D** *Top*: distribution of trials collected from previously trained mice (19,896 trials across 7 mice), using a 5 s sampling window, $\lambda$_1_ = 12 and $\lambda$_2_ = 4. Gray dashed line indicates the location of the decision boundary (8.5 pulses). *Bottom*: psychometric curve of the pooled behavioral responses across animals under the experimental conditions described above. Orange circles indicate the mean, whereas the shade represents the 95% CI. (**E**) Mixed-effects logistic regression coefficients obtained after fitting the behavioral choices to the ‘Null’ and ‘Full’ models using odor information divided in 5 bins. Models were compared using a log-likelihood ratio one-sided test with the *p*-value of the comparison indicated. Data is expressed as mean ± CI 95%. Vectorized images from SciDraw (doi.org/10.5281/zenodo.3925903 and doi.org/10.5281/zenodo.3925921) were adapted for the schematics in **A**–**C**, licensed under CC BY 4.0 (https://creativecommons.org/licenses/by/4.0/deed.en), with changes made.

We trained water-restricted, head-fixed mice in a two-alternative forced choice (2AFC) task in which the rewarded side was determined by the total number of odor pulses delivered. Animals were presented with sequences of 50 ms odor pulses of 5% ethyl valerate over a time window of 5 s, and the side of the reward was determined by whether the total number of odor pulses presented was above or below a fixed threshold (Fig. 1A, B). Photoionization detector (PID) recordings obtained directly from the mask showed that the 50 ms voltage commands to the odor valve triggered an odor signal with ~40 ms halfwidth (Supplementary Fig. 1A), confirming that our olfactometer can produce sub-sniff olfactory stimuli. Each trial of the 2AFC task (Fig. 1C) started with a sound cue marking the beginning of the sampling period in which the sequence of 50 ms odor pulses was delivered to the mask. The total number of pulses delivered in each trial was drawn with equal probability from one of two truncated Poisson distributions in which the overlap is removed, leading to trials in which the total pulse count was above or below the threshold (High and Low Trials, respectively). A second sound cue denoted the end of the sampling period and the initiation of the response period in which the animal had to lick to indicate its behavioral choice. The two water ports were respectively associated with high or low number of odor pulses presented during the sampling period, and this association was counterbalanced across animals. When the mouse licked the correct water port, it was rewarded with a drop of water. When the mouse made the wrong decision, no water was given, a warning buzz sound was played, and the mouse was put under a 10-second timeout as a punishment before the next trial started.

After training was completed, we analyzed the behavioral performance of mice when they were challenged with a 5 s sampling period and a ratio of 3 between the means of the two Poisson distributions used for generating the odor pulses (Fig. 1D, top panel). In this context, the boundary between Low and High Trials was between 8 and 9 odor pulses. Performance approached saturation (>~90% correct) when mice differentiated between stimuli with pulse count far from the decision boundary and degraded when pulse counts approached the boundary (Fig. 1D, lower panel). Animals were also able to discriminate total pulse counts when odor pulses were delivered over shorter (1.25 s, 2.5 s) or longer (10 s) sampling windows (Supplementary Fig. 1C), suggesting that mice can integrate olfactory evidence for decision-making over a wide range of intermittencies and times (Supplementary Fig. 1D). Learning or performance in the task did not rely on the integration of a property specific to ethyl valerate since a separate cohort of animals (*n* = 3) could be trained using 50 ms odor pulses of 5% methyl butyrate (see Supplementary Fig. 1A, bottom, for the PID traces) and reached similar performance in the testing condition (Supplementary Fig. 1E, green traces). To test if mice were using pressure or airflow changes as cues for decision making, we introduced catch trials with varying number of clean air pulses (instead of the odorized air), which were rewarded by following the same rule as for the odor pulses. Catch trials were categorized by mice as ‘Low’ trials irrespective of the total pulse count (Supplementary Fig. 1E, gray traces), confirming that they were basing their decision on the integrated number of odor inputs rather than mechanosensory or auditory signals arising from any airflow transients or valve openings.

Odor pulses were presented non-uniformly and stochastically during the sampling window. In principle, an accurate decision in every trial must result from an evidence accumulation process, in which the animal must integrate sensory information throughout the trial, and not just a short time window. To measure the extent to which the pulses in different parts of the trial contribute to the animal’s decision, we divided the sampling window into 5 bins (1 s each) and calculated the mean odor signal for each bin in each trial. The binary licking decisions were then fitted using two mixed effects logistic regression models: a null model in which all time bins were forced to have equal coefficients and a full model in which coefficients were allowed to differ across time bins. The full model provided a better fit to the data and was significantly different from the null model (Fig. 1E, Log-likelihood ratio test, *p*-value: 8.29 E-10), suggesting that animals weigh sensory information coming over several seconds differentially. Altogether, our newly developed task reveals how mice accumulate and weigh odor information over several seconds for decision-making.

### Breathing influence on behavior

**Fig. 2:**
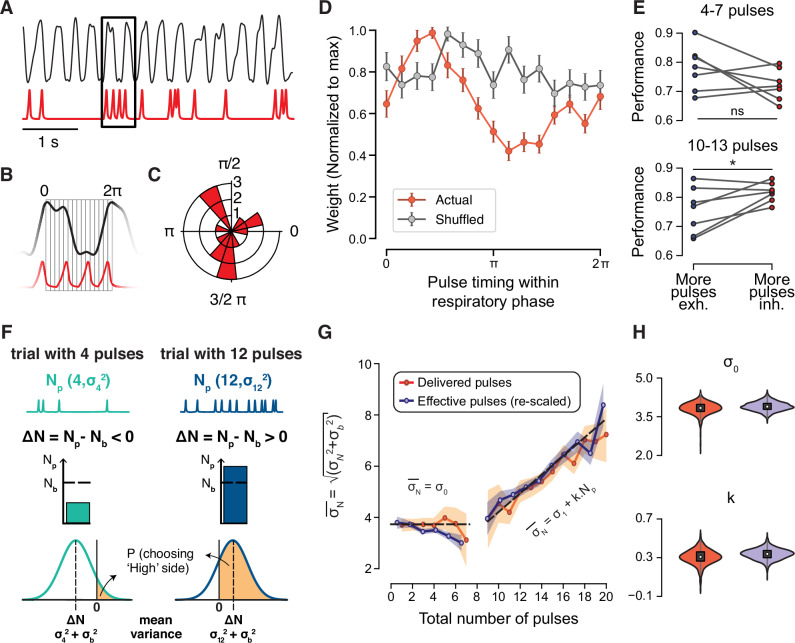
Breathing constrains odor sampling and affects choices but not noise scaling. **A** Breathing signal (black trace) and odor signal (red trace) in example trial. **B** Expansion of traces for black box in (**A**). Each breath was divided into 15 bins from 0 to 2π for sorting odor pulses. **C** Phase histogram showing the number of pulses arriving at each bin for all the breaths in trial depicted in A1. **D** Bootstrapped (n_boot = 15,000) normalized coefficients (weights) vs. phase bins obtained after fitting a binary logistic regression model for mice choices. ‘Actual’ (orange): weights obtained based on original phase histograms. ‘Shuffled’ (gray): weights obtained after shuffling pulse timing in each trial before fitting. Data is presented as mean ± SD. **E** Comparison of within-animal performances for trials with more pulses falling during exhalation or inhalation. Each dot represents mean animal performance (*n* = 7 animals) averaged across sessions for trials with 4–7 total (top) or 10–13 total pulses (bottom). *p = 0.021, one-sided paired t-test. **F** Decision noise model rationale. *Top*: *N*_*p*_ follows a Gaussian distribution with a mean at the true number of pulses and a variance value (σ_N_^2^) for each value of *N*_*p*_. *Center*: ΔN is calculated by subtracting *N*_*p*_ from the estimate of decision boundary, *N*_*b*_, also following a Gaussian distribution with variance σ_b_^2^. *Bottom*: ΔN follows a Gaussian distribution, with the orange shade representing the probability of choosing the ‘High’ side. **G** Bootstrapped (n_boot = 1000) Maximum Likelihood Estimation (MLE, see Methods) of $\bar{{\sigma }_{N}}$ for each value of *N*_*p*_ based on delivered pulses (orange trace) or the effective pulse count after adjusting for breathing (purple trace). Data is presented as mean ± SD. **H** Violin plots of σ_0_ and k values obtained from linear regression of the bootstrapped $\bar{{\sigma }_{N}}$ estimations based on delivered *N*_*p*_ (orange) or re-scaled effective *N*_*p*_ (purple). White dot, box, and whiskers in box plot represent median, IQR and 1.5*IQR, respectively. 95% Confidence interval of the difference in σ_0 Delivered_ = (−0.73, 0.43), and k = (−0.24, 0.14). All data in the figure was derived from 19,896 trials from 7 mice.

The sampling of olfactory inputs by mice is tied to the airflow driven by inhalation and exhalation during breathing, or its active variant, sniffing. While there was a slight trend towards lower breathing rate over the course of task learning (Supplementary Fig. 2A), trained mice showed a stable breathing rate of ~3 Hz that was not affected by odor pulse presentation (Supplementary Fig. 2B). Therefore, the amount of odor molecules inhaled from each pulse will depend on the arrival time of a given odor pulse relative to the breathing cycle. To test how this variability affects behavioral choice, we divided each breath into 15 bins and computed a ‘phase histogram’ representing the number of pulses that arrived at each phase bin across successive breaths on a trial (Fig. 2A–C). When phase histograms were used as the input to train a logistic regression model to predict the licking decisions of mice in each trial, we observed that the perceptual weights (i.e., the logistic regression coefficients associated with each bin) were maximal around the peak of the inhalation and decreased progressively towards the exhalation (Fig. 2D, orange trace). The phase-dependence was absent when the model was trained on the same data but shuffling pulse timing during the trial (Fig. 2D, gray trace).

Additionally, by computing the dot product between the phase histogram in each trial with the mean perceptual weights from logistic regression, we were able to estimate the effective number of pulses experienced by mice in each trial (Supplementary Fig. 2C). We found a linear relationship between the delivered pulse count and the effective pulse count, with increased variance for higher pulse counts (Supplementary Fig. 2). When effective pulse counts were binned (Supplementary Fig. 2E) and used to re-compute a psychometric curve, the relationship between choice and the amount of sensory information was steeper than was observed in the original psychometric constructed using delivered pulses (Supplementary Fig. 2F). These findings inspired us to classify trials based on whether more than 50% of pulses on a trial fell on the inhalation or exhalation phase (Fig. 2E). We then looked at a subset of trials with total pulse counts (4–7 pulses) below the decision boundary and found no significant differences in the performance of each mouse between the two groups of trials (one-sided paired t-test, *t* = 1.687, df = 6, *p*-value: 0.071). However, when we looked at trials with total pulse counts above the decision boundary, within-animal performance was significantly higher for trials with the majority of pulses arriving around inhalation (one-sided paired t-test, *t* = −2.579, df = 6, *p*-value: 0.021; Fig. 2E). Therefore, these results indicate that the timing of fluctuating stimuli relative to the breathing cycle determines its perceptual weighing for decision-making and suggest that part of the mistakes made by animals arose from partially or totally missed pulses that arrived in proximity to or during exhalations.

### Models to fit behavioral data

Our experiments describe how well mice categorize odor pulses arriving in the sampling period into High or Low, and how the relative timing of a single odor pulse relative to the animal’s respiratory cycle impacts its weighing in the context of the task. These variables affect not only how mice arrive at the estimate of the total pulse count in each trial, but also their estimate of the decision boundary used for categorizing trials as High or Low. Since both quantities are internally estimated by mice, there is uncertainty (or noise) associated with them. These uncertainties could, in principle, account for the shape of the psychometric curve, including the shallow dependence on the pulse number and the asymmetry around the decision boundary, as well as the deviation from the performance predicted by a model of a binary choices based on two different Poisson rates (Supplementary Fig. 3A). Inspired by a previous report^33^, we built a decision model where the perceptual estimation of the odor pulse number was Gaussian-distributed around the total number of odor pulses delivered in a trial (*N*_p_), with a variance σ^2^_N_, which could take different values for different *N*_p_ (Fig. 2F). We also considered that the animal needs to generate an estimate of where the boundary between the High and Low conditions (*N*_*b*_) lies in the sensory space, with its corresponding error σ^2^_b_. Altogether, the ΔN value - the difference between *N*_p_ and *N*_b_ - should inform the decision of the animal: the mouse should choose the High side when ΔN > 0, and the Low side in the opposite case (Fig. 2D), with an error $\bar{{\sigma }_{N}}$ reflecting the contributions of σ^2^_N_ and σ^2^_b_. Bootstrapped estimations of $\bar{{{{\rm{\sigma }}}}_{{{\rm{N}}}}}$ for each value of *N*_*p*_ were generated using maximum likelihood estimation (MLE) and by setting the value of *N*_*b*_ = 8: the pulse count closest to 0.5 in the average psychometric curve. $\bar{{{{\rm{\sigma }}}}_{{{\rm{N}}}}}$ values were constant for low values of *N*_p_ but eventually started to scale with *N*_p_ (Fig. 2E, orange trace).

Empirical evidence across tasks involving the simultaneous or sequential integration of quantities has supported a diverse set of models to explain the relationship between decision noise and sensory stimulus quantity^33–35^. We found that any model assuming noise scaling with the number of pulses provided better fits to the behavioral data than a model assuming a constant noise across different numbers of pulses delivered, but the best fit to the data corresponded to a model assuming a non-zero constant noise for low pulse quantities, followed by a linear scaling of noise with higher pulse counts (Supplementary Fig. 3B–D). Next, we wanted to explore whether breathing sets any constraints in the scaling of decision noise. We binned our effective pulse counts and used them to generate new estimates for $\bar{{{{\rm{\sigma }}}}_{{{\rm{N}}}}}$ using MLE with *N*_*b*_ = 5.01 (again, the bin center closest to 0.5 in the effective psychometric curve, Supplementary Fig. 3E), and then re-scaled $\bar{{{{\rm{\sigma }}}}_{{{\rm{N}}}}}$ values using the regression shown in Supplementary Fig. 2D. $\bar{{{{\rm{\sigma }}}}_{{{\rm{N}}}}}$ values obtained from the effective pulse counts (Fig. 2G, purple trace) showed the same trend as the estimates generated with the delivered pulse count, and no significant differences were found in the values of σ_0_ and k, the parameters of the model that provided the best fit to data for the constant and linear parts of the plot, respectively (Fig. 2H). This result is also supported by the fact that the psychometric curve built using effective pulse counts still shows an asymmetric dependence of choice with odor pulse counts. Taken together, our results suggest that decision noise is not constant across the whole range of total number of stimulus pulses that mice can experience in the task, and such variation is not related to sampling.

### Sensory input constrains behavior

**Fig. 3:**
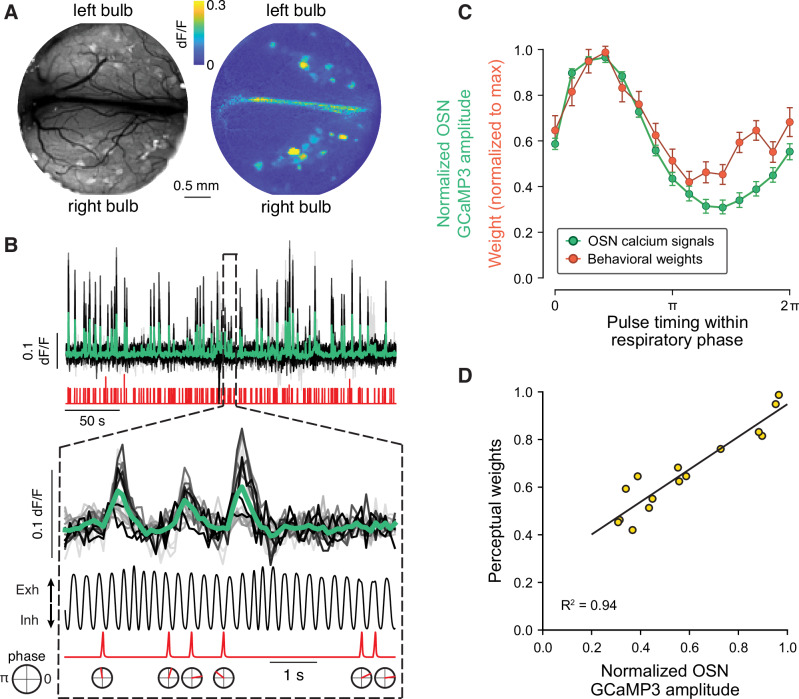
Perceptual weights correlate with odor-evoked OSN activity. **A** Top view of the cranial window over the OB showing GCaMP3 signals in the OB during pulse presentation (**left**: raw image; **right**: dF/F). The ROIs with high dF/F values correspond to glomeruli responding to an odor pulse. **B** dF/F traces from multiple glomeruli (top trace) in response to odor pulses (bottom trace). Traces corresponding to single glomeruli are shown in grayscale, whereas the average across glomeruli is shown in green. Traces in the dashed gray box in the top panel are displayed enlarged at the bottom, with the addition of the breathing trace and a depiction of the arrival time of each of the pulses relative to the respiratory signal. **C** Green curve: Average pulse-triggered calcium signals (normalized to the maximum response) plotted against the phase of the breathing cycle in which the pulse arrived. Error bar corresponds to the 95% confidence interval. Amplitude of pulse-triggered calcium responses depended on the arrival time of odor pulses relative to the breathing cycle. Behavioral data from Fig. 2D is reproduced here for comparison (orange) and is expressed as mean ± sd (*n* = 15,000 bootstrapped samples). **D** Scatter plot of the pairs of phase bin weights for choice prediction and the normalized averaged OSN activity for each bin, and the corresponding regression line (black trace, R^2^ = 0.94; *p* = 1.41 E-7, two-sided t-test). Sample size of calcium imaging data (*n* = 3 animals).

Having discovered that the respiratory cycle governed the weighing of olfactory stimuli for the behavioral responses, we were interested in testing if that dependence was already apparent in the activity in the very first stage of odor processing: the olfactory bulb glomeruli. Therefore, we evaluated the odor pulse-triggered activity of the axonal terminals of the OSNs through wide-field calcium imaging in awake mice expressing GCaMP3 in OSNs (Fig. 3A). Several glomeruli in the dorsal surface displayed detectable responses to pulses of ethyl valerate (Fig. 3B), with their response timing reflecting the known kinetics of GCaMP3^36^, which validates the brevity of the odor pulse stimuli. The magnitude of pulse-triggered calcium signals was highly variable, even within a single glomerulus (Fig. 3B; the 3rd, 5th and 6th pulses elicit negligible responses). To address if that variability in the responses was due to a breathing modulation of the sensory input, we took the same approach we used above for the behavioral data and computed the amplitude of the GCaMP3 signals as a function of pulse arrival time relative to the respiratory cycle. The average phase response curve revealed that odor pulses arriving around the peak of the inhalation phase elicited the strongest OSN calcium responses, whereas the weakest responses corresponded to pulses arriving during exhalation (Fig. 3C, green trace), mirroring the dependence observed for the bins of the phase histogram for binary prediction of choices (Fig. 3C, orange trace). The phase-dependence of calcium signals was observed across all the glomeruli responding to the odor pulses (Supplementary Fig. 4). In fact, there was a high correlation between the logistic regression coefficients and the mean GCaMP3 pulse-triggered responses (Fig. 3D, *r* = 0.94, *p* = 1.41E-7). Hence, the differential weighing of pulses for decision-making as a function of their arrival time in the respiratory cycle is likely constrained by the intensity of OSN responses to each pulse.

### Olfactory cortical responses largely reflect sensory information

The anterior piriform cortex (APCx) is one of the main areas receiving inputs from the olfactory bulb glomeruli, and it, in turn, connects to the prefrontal cortex. These observations have supported the idea of APCx as a possible hub for transformation of the olfactory representations into a category-related signal interpretable for decision-making centers in the brain^37–41^. Therefore, we were interested in assessing how the sequences of intermittent odor pulses were represented in the APCx in expert mice.

**Fig. 4:**
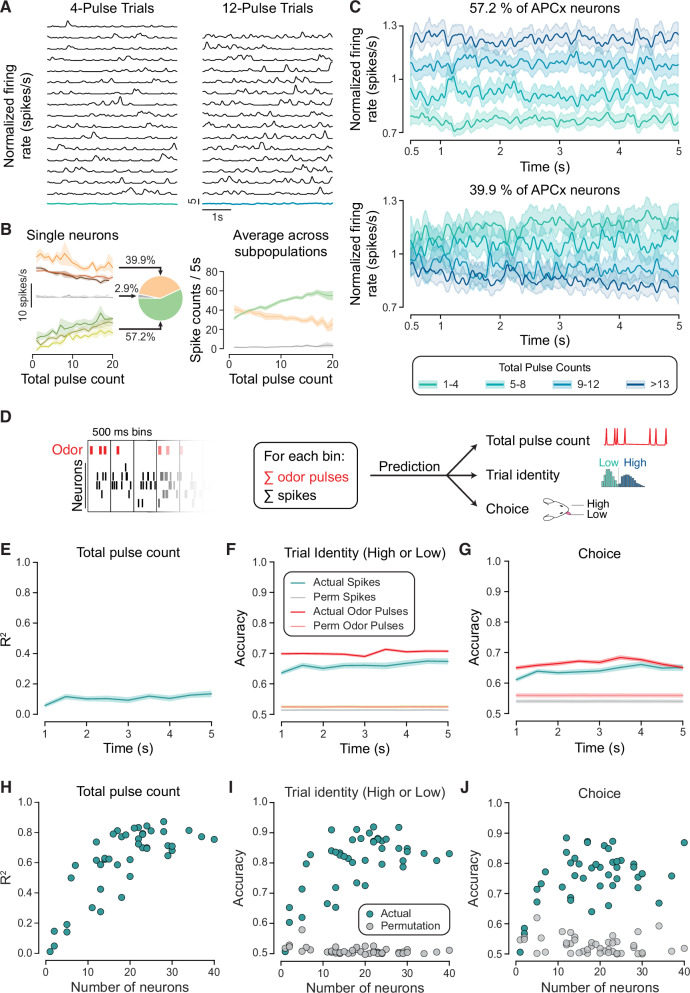
APCx firing rates do not show signs of perceptual evidence accumulation. **A** Firing rates of an APCx neuron across all trials in a session with 4 (**left**) or 12 pulses (**right**). Colored traces: average across trials. **B Left**: Average spike counts over the 5 s sampling window vs. total number of pulses in a trial. Each colored line represents a single neuron, whereas shaded bands correspond to 95% CI. **Center**: Distribution of APCx neurons based on firing rates becoming higher (green, 57.2% of APCx neurons), lower (beige, 39.9% of APCx neurons) or unaffected (gray, 2.9% of APCx neurons) with increasing number of pulses. **Right**: Average spike count for the three subpopulations vs. total number of pulses in a trial. **C** Average firing rates of neurons from the green (top) and beige (bottom) groups in B for different ranges of pulse numbers. **D** Decoding analysis rationale. Trials were divided in 500 ms bins, with summed pulse counts and spikes calculated for each bin. For each trial, total pulse count, trial identity and choice were predicted based on spike counts or odor pulse counts in each bin. **E** Average multiple linear regression R^2^ of total pulse count prediction using population spike counts over independent 500 ms intervals. Prediction accuracy of logistic regression on trial identity (**F**) and behavioral choice (**G**) using spike counts (teal traces) or odor pulses (red traces) over independent 500 ms intervals. Gray and pink traces are the accuracies when target labels were permuted for predictions based on spikes and odor pulses, respectively. **H** Average R^2^ score of linear regression between spike counts and total pulse count in a trial against the number of recorded neurons in a session. **I**−**J** Same as **H**, but for the dependence in the accuracy of logistic regression prediction on trial identity (**I**) and animal’s decision (**J**). Teal dots indicate the accuracies calculated based on the actual data, whereas gray dots are the accuracies after permutation of samples. Data is shown as mean ± CI 95%, *n* = 3 animals (over 17, 13, and 12 behavioral sessions). Vectorized images from SciDraw (doi.org/10.5281/zenodo.3925903) were adapted for the schematics in D, licensed under CC BY 4.0 (https://creativecommons.org/licenses/by/4.0/deed.en), with changes made.

Extracellular spike recordings in APCx from trained animals during behavior revealed that the activity of many neurons was correlated with the delivery of odor pulses, exhibiting greater activity fluctuations over the 5 second period with more pulses (Fig. 4A). We selected neurons whose activity was modulated by odor stimuli using a predictive criterion as described in the Methods (see *Analysis of APCx spiking data* in the Methods section). We found that many neurons exhibited graded activity (summed over 5 s) as pulse counts changed (Fig. 4B). We first analyzed the variation in firing rates of neurons across trials with different total pulse counts (binned into 4 groups: 1–4, 5–8, 9–12, >13 pulses). We normalized the activity of a neuron in each trial to the mean activity across all trials, and classified neurons based on activity being above the mean with increasing number of pulses (57.2%), above the mean with decreasing number of pulses (39.9%), and relatively homogeneous across all pulse counts (2.9%). We then computed - for the two main groups - the average firing rates for different ranges of total pulse counts during the sampling window of the task (Fig. 4C). We interpret the first group (Fig. 4C, top) to have odor pulse-triggered increases in activity, and the second group (Fig. 4C, bottom) to arise from odor-triggered inhibition of activity, such that these neurons spike less when there are more pulses in a trial. Importantly, we did not observe a significant cumulative increase in the firing rate over the 5 s period, as proposed for evidence accumulation areas in other studies^42–44^.

The overall flat structure of the average activity of neurons over the trial period of 5 seconds suggests that the information encoded by APCx neurons reflects ongoing sensory input. To test whether there was some latent accumulating information not revealed by simple trial averaging, we performed different decoding analyses to test whether the APCx activity in the late sampling period close to the time of decision making was different from earlier periods (Fig. 4D). We calculated the R^2^ value of linear regression between the spike counts for each trial in successive 500 ms windows (a total of 10 such bins in 5 s) and the total number of pulses in the trial; this regression value was low and uniform across the entire sampling period (Fig. 4E). We also used a logistic regression model to predict the trial identity (Low or High), as well as the animal’s choice using the spike counts in the successive 500 ms sampling window (Fig. 4F, G). Accuracy of predictions on actual data (‘Actual spikes’) was higher than for data in which labels were shuffled (‘Perm spikes’), but still below the maximal accuracy given by the actual stimulus pulse (‘Actual Odor Pulses’). Remarkably, accuracy levels of predictions made from spike counts were uniform, with no evidence that there was higher decodability closer to the decision time. Decoding based on cumulative 500 ms intervals revealed a higher accuracy when spikes over the whole sampling period were considered, but in agreement with the previous results, no abrupt increases in decodability were seen for later intervals (Supplementary Fig. 5A–C).

Neural activity in short time windows in APCx was not sufficient to predict task variables. We next investigated whether any downstream areas integrating information from APCx neurons over the entire 5 s sampling period will be able to recover task information. First, for each recording session, we calculated the R^2^ value of the linear regression between the total spike counts of all neurons and the total pulse count over the 5 s period in a trial. Across different sessions, this value increased with the number of recorded neurons available until a plateau was reached for population sizes higher than 20 neurons (Fig. 4H). The ceiling of 20 neurons was also observed when total spike counts were fed into a logistic regression model to predict trial identity (Fig. 4I) or the behavioral choice (Fig. 4J). Cumulative population spike counts predicted trial identity better than choice (comparing Fig. 4I, J). These data indicate that collective neural activity in a small population of APCx neurons over the entire 5 s period can be integrated downstream to make accurate binary decisions, but the activity in APCx at the end of the sampling period does not reflect decision variables.

### Detailed structure of cortical responses to fluctuating stimuli

**Fig. 5:**
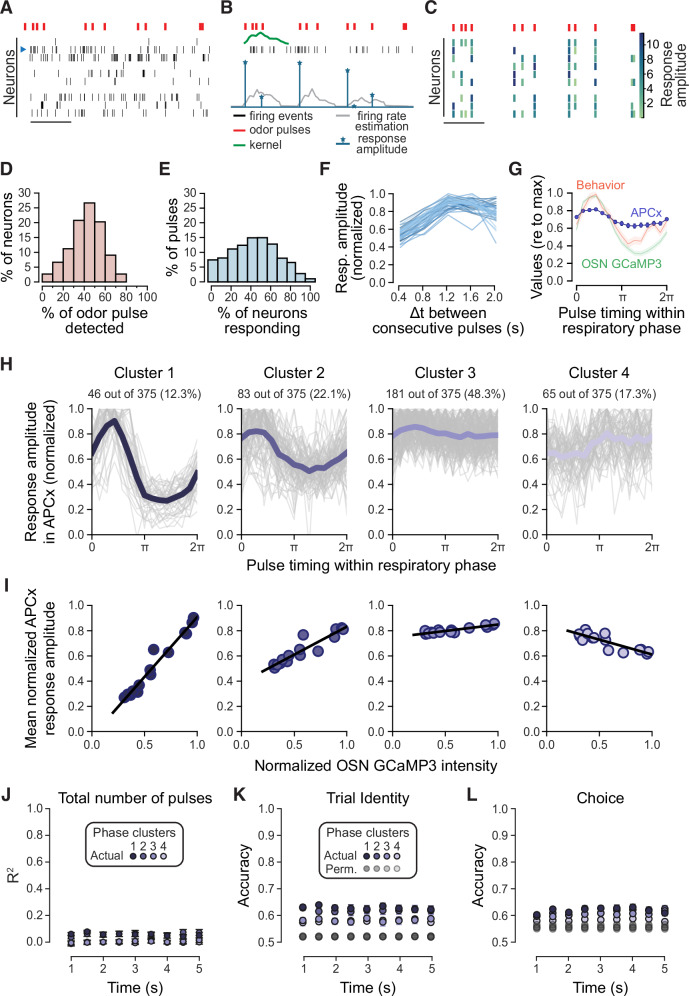
APCx population responses to odor pulses are stochastic and heterogeneous. **A**−**C** Example of DUNL implementation for the neuron marked with the blue arrowhead in the raster plot shown in (**A**) for a specific trial. **B** Timing of spikes and odor pulses are used to train a model that learns a specific event-based kernel for each neuron (‘kernel’, green trace), as well as which sensory stimuli were detected and the intensity of the neuronal response (blue trace, also referred as ‘response amplitude’). Convolution of response amplitudes with the kernel provides an estimation of the firing rate of the neuron (‘firing rate estimation’, gray trace). **C** Same as **A**, but with every line showing the response amplitude in a color-coded scale. Scale bar in A,C: 1 s. **D** Distribution of the percentage of neurons detecting different proportions of odor pulses presented. **E** Distribution of the percentage of odor pulses that elicited spiking in different proportions of the neural population. **F** Variation in response amplitudes as a function of the time between consecutive pulses. Each curve is the average for individual neurons. **G** Average pooled APCx response amplitudes (mean ± CI 95%, normalized to the maximum of each neuron) against pulse timing within the respiratory cycle. Behavioral weights (mean ± sd, orange trace) and calcium signals in OSNs (mean ± CI 95%, green trace) are displayed for comparison. **H** K-means clustering of normalized APCx neuronal responses vs. arrival time of odor pulses. Colored traces: average responses of all neurons in cluster. Gray traces: single-neuron responses. **I** Mean APCx phase responses in each cluster plotted against mean OSN responses (from Fig. 3C) for each of the 15 bins of the respiratory phase. *n* = 3 animals over multiple recording sessions. **J**–**L** Same as in Fig. 4C–E but using spikes over 500 ms independent intervals from neurons in each of the 4 clusters on H for decoding. Data is shown as mean ± CI 95%, *n* = 3 animals over 17, 13 and 12 behavioral sessions.

The stochastic nature of stimulus presentation and the non-repeating structure across trials in our task precluded simple trial-averaging to reveal neuronal responses to individual odor pulses (which will occur at distinct times across trials) (Fig. 5A). To overcome this limitation, we implemented a deconvolutional unrolled neural learning (DUNL) method^45^, which uses a deep learning framework to learn locally low-rank structures from neural data. In particular, it is useful in characterizing neural responses to sparse discrete events in experiments with non-repeating trial structure or with no structure. We used DUNL to independently learn, for each neuron, its response kernel to odor pulses as well as the response intensity to each specific pulse (Fig. 5B, C). Different neurons had distinct response kernels (which are essentially the impulse responses of each neuron to the odor pulse), but K-means clustering revealed the existence of 4 different groups, suggesting that the population of APCx neurons was heterogeneous in terms of how each neuron transformed the sensory input into firing (Supplementary Fig. 5D). Using this method, we were also able to estimate that each odor pulse activated a subpopulation of recorded neurons, but the size and neuronal identities of those subpopulations varied from pulse to pulse. Overall, a neuron detected ~50% of odor pulses (Fig. 5D), and an odor pulse triggered the activation of ~50% of the recorded neurons (Fig. 5E). These findings clearly indicate that reliable encoding of each odor pulse must occur at the population level, rather than at the level of single neurons, in line with our analysis in Fig. 4.

In addition to revealing which specific event any given neuron responded to, the DUNL method allowed us to infer a representation, estimating the intensity of odor response (“response amplitude” in Fig. 5C) from the spiking frequency of APCx neurons. We then asked whether the intensity of the response to a given odor pulse in a single neuron was affected by the timing of the preceding odor pulse. We found that response amplitudes were reduced for the second pulse when the time between pulses was less than one second, presenting a signature of sensory adaptation in APCx neuronal firing (Fig. 5F). This analysis of pairwise interactions did not consider the timing of the pulse arrival with respect to the breathing phase but averaged it over all the different arrival times.

Since olfactory sensory responses were modulated by the respiratory phase (Fig. 3C), we next analyzed the relationship between cortical neuron responses and the timing of the pulses within the respiratory phase. We calculated the average response amplitude for all neurons after binning the stimuli in the same 15 bins as for behavioral and OSN calcium data. Compared to OSNs responses or the behavioral weights, the dependence of response intensity on the phase of pulse arrival was shallower, but pulses arriving around the peak of inhalation still triggered the largest responses (Fig. 5G). K-means clustering of the normalized response amplitudes of individual APCx neurons over the entire respiratory phase suggested the existence of four different functional groups (Fig. 5H). For comparison, we plotted the mean normalized response amplitudes in each cluster and the normalized average OSN responses from the bulbar imaging, with their corresponding linear regression (Fig. 5I). One cluster -comprising ~50% of the APCx neurons- showed no respiratory phase tuning (Fig. 5H, Cluster 3), whereas the other three clusters displayed different levels of modulation of the neuronal responses by pulse timing during a breath (Fig. 5H, Clusters 1,2,4). The distributions of response intensities were not significantly different across phase clusters (Supplementary Fig. 5E, F), suggesting that the observed phase modulations are not due to difference in the amplitude of neuronal responses. The clustering of modulatory shapes was supported by further analysis based on individual neurons and kernel density estimation of the regression slopes (Supplementary Fig. 5G–I). No correspondence was found between the clusters of neurons arising from the respiratory phase modulated responses and clusters based on the kernel shape (Supplementary Fig. 5J). Therefore, fast, intermittent olfactory stimuli are represented in the APCx by neuronal ensembles that are heterogeneous in terms of their respiratory phase tuning and firing properties, features that may underlie the stochastic recruitment of neurons upon odor-pulse presentation.

Our behavioral results indicate that odor pulses arriving at different phases of respiration have different perceptual weights (Fig. 2D). Brain regions downstream of APCx may be able to interpret the total intensity of global APCx output (Fig. 5G) as modulated by respiration to make the necessary perceptual decision. However, it is also possible that the downstream decision-making brain regions could be informed largely by those neurons with a particular functional group of neurons – for example, those with strong respiratory modulation (Fig. 5H, I, left) but not by those APCx neurons whose responses are independent of the respiratory phase (Fig. 5H, I, right). If this were the case, the different groups of neurons identified from the clustering of the phase responses (Fig. 5H) will differ in their decoding accuracies in predicting either trial type or behavioral choice. This prediction was confirmed: when task-related variables were predicted based on spikes from neurons belonging to the cluster with maximal modulation by respiratory phase, logistic regression scores were significantly higher than for neurons with lower tuning to the arrival time of the input during the respiratory phase (Fig. 5J–L).

In summary, the responses of APCx population triggered by odor pulses showed little to no signatures of evidence accumulation. Task-related variables could be represented through small subsets of neurons, with the fidelity of the encoding being affected by the phase-tuning and firing pattern of the individual neurons.

## Discussion

Using a novel behavioral task, we have demonstrated that mice can recognize differences in features of fluctuating olfactory stimuli that become apparent only when integrated over several seconds. We also showed that mice valued the odor pulses differentially depending on their timing with respect to the respiratory phase, and that this heterogeneity stems from phase-dependent changes in the amplitude of OSN responses. Modeling of mouse choices revealed that pulse timing relative to the breathing phase accounts for some of the mice’s choice errors. APCx neurons responded stochastically to odor pulses within a trial and their response time courses were heterogeneous. APCx neuron responses were also heterogeneously modulated by the respiratory cycle, suggesting a multiplexed stream of information diverging from highly tuned glomeruli to different neuronal subpopulations in the APCx. While many APCx neurons scaled (up or down) their firing rates with the total odor pulse count, the average firing rates were constant over the duration of the sampling window, rather than ramping up or down over time. This absence of directional changes in firing rates over time with increasing sensory information rules out APCx as a potential olfactory evidence accumulation center. Altogether, our study provides insights into the physiological and behavioral representations of fluctuating stimuli in the context of an olfactory evidence accumulation task.

### The behavioral task

Mice in our task were asked to distinguish and categorize random sequences of brief odor pulses of the same odorant based on whether the integrated signal was below or above a threshold. Importantly, PID recordings confirmed that, when pulses happened close in time, odor signals could sum and lead to short intervals of higher intermittency and overall amplitude. Therefore, by building stochastic sequences of odor pulse delivery, our olfactometer could successfully recreate naturalistic odor profiles. The signal integrated by mice for decision-making could be related to one or a combination of statistical features that characterize odor plumes: intermittency, whiff (or blank) count and frequency and concentration profile^8^. In the current task, the duration of each pulse and the duration of the sampling window were fixed, allowing us to control the values of those statistics by varying the total number of odor pulses delivered. For instance, by increasing or decreasing the size of the sampling window but keeping the total pulse counts the same, we could manipulate the range of intermittencies of the olfactory stimuli presented (Supplementary Fig. 1D). Mice were not only capable of categorizing odor stimuli across sessions with different ranges of intermittencies (Supplementary Fig. 1C), but more remarkably, they were able to accurately discriminate pulse counts at opposite sides of the decision boundary that only differed in ~0.05 units of intermittency, a value significantly lower than the one shown in a recent report^32^. Although we cannot rule out the possibility that mice are capable of extremely fine intermittency discrimination, the fact that they performed correctly across different intermittency ranges but same pulse counts supports the idea that they were integrating odor pulse counts rather than intermittency. It is also important to note that which statistic is being integrated can also be a result of the experimental design: the sensory stimuli in our task was composed of a stochastic array of odor pulses with temporal properties that supported their discretization, whereas in Gumaste (2024) animals were trained in odor profiles that varied in intermittency but with a lower range of variation in the number of whiffs. Therefore, our observation of odor pulse (whiff) count discrimination independent of intermittencies is not necessarily in conflict with their observation of intermittency discrimination irrespective of whiff counts. The question regarding which feature or statistic of olfactory stimuli animals integrate during odor-guided navigation is still open, but our results indicate that mice are capable of integrating whiff counts for decision-making. Assays acutely manipulating the duration of the sampling window during catch trials have been used as a strategy to disambiguate the variable that animals integrated^46^, and they can be incorporated in future experiments.

The present work also confirms that the temporal integration of olfactory information can occur over time windows covering multiple respiration cycles, in agreement with previous reports using fluctuating stimuli^28,47^. The random delivery of pulses within the sampling window led to trials with equivalent total pulse counts that differ in their temporal profiles, but more importantly, also allowed trials with pulse counts at both sides of the decision boundary to display similar statistics over short intervals. Good performance in this task requires a decision-making process guided by sensory evidence accumulation, in which mice have to store transient changes in olfactory statistics in a signal that needs to be updated upon encountering newer stimuli. In this context, we observed mice have the flexibility to accurately discriminate stimuli presented in windows ranging from ~1 to 10 s (Supplementary Fig. 1B), similar to what has been reported in rodents for discrimination of visual flashes^33^ or integration of object quantities in a virtual reality setup^48^. Whereas sub-second integration times can be detrimental for performance^49^, there is still an open question about whether evidence accumulation over large timescales is achieved by time-invariant cognitive mechanisms or by flexible adaptation of those mechanisms across different timescales^50^. For instance, regression analysis on the licking decisions made on 5 s trials revealed that mice place different values to olfactory information depending on its timing during the sampling window (Fig. 1E). The reduced weights for inputs coming at the beginning of the sampling period implies the absence of a primacy effect for decision-making, suggesting that the storage of early sensory information could be affected by leakage in the integrator, similar to what was previously shown for rats performing multisensory integration for binary choices^46^. On the contrary, the reduced weights towards the end of the sampling period could, in principle, indicate the lack of a recency effect. However, it is also possible that the decrease in perceptual weights by the end of the sampling period could be explained by animals already committing to a decision and then neglecting sensory inputs coming afterwards, as it has been proposed by recent work modeling neural dynamics during binary decision-making^51,52^. In that scenario, animals could be reaching a decision commitment before the end of sampling period and the increased perceptual weights in the middle could be reflecting a recency effect relative to decision commitment. Future experiments with neural recordings from brain areas carrying explicit information about the accumulated olfactory evidence in the task could help to clarify this issue.

Sensory evidence accumulation is not only defined by the memory displayed by the accumulator, but also by the level of noise in the accumulating signal. Applying a similar approach to a previous report^33^, we showed that noise in the estimation of pulses was initially constant for low pulse counts until a point after which it started to scale with the number of pulses (Fig. 2G). Constant noise in the estimation of low quantities has been shown before in multiple animal and human experiments, and has been explained by the concept of ‘subitizing’^34,35^. Subitizing refers to the perceptual process allowing animals to immediately provide a correct estimation (noiseless) of the total number of perceptual items when the number of elements is small (usually below 5)^53,54^. However, our noise modeling results challenge subitizing in two main aspects: (i) the estimation of low quantities is not noiseless and (ii) the constant noise extends further than four to five stimuli^35^. Regarding the first point, noise values were significantly reduced when model parameters were estimated using the perceived odor in a trial -i.e., the odor pulses convolved with the sniff kernel- (Supplementary Fig. 2F), suggesting that part of the basal noise derives from perceptual variabilities due to sampling, but the source of the remaining noise offset is still unknown. Regarding the second point, it is important to note that the majority of reports about the limits of subitizing come from human experiments using visual or auditory stimuli, whereas larger limits were found for animal subjects^34^. Once noise starts to scale with the number of pulses, it is better fitted by a scalar variability model in which the noise scales linearly with the stimulus count. The persistence in the noise scaling after accounting for breathing, suggests that noise scaling is independent of sampling. Scalar variability has been observed in other rodent behaviors involving perceptual evidence accumulation^33,55^, and it is believed that it can emerge from the neural mechanisms used by the brain to encode quantity estimations^56^. As noted above, although models for counting discrete stimuli seem to be a valid approach to approximate the accumulation of olfactory statistics in our task, modeling results might not be sufficient to prove that mice are implementing a counting strategy over discretized statistics - such as odor pulse/whiff counts - as opposed to just accumulating temporal variations of a continuous variable such as odor intermittency or pulse rate.

### Breathing modulation of sensory information

The brief nature of the odor pulses used in our task was essential for testing the effects of breathing in the representation of olfactory stimuli across the ascending olfactory pathway and its later weighing for behavior. Previous reports have shown that MTC responses to steady odor stimuli tile the respiratory phase with a bias towards inhalation^20,23,57^. Here, we show that OSN responses were larger for pulses arriving during inhalation than for pulses arriving during exhalation, suggesting that breathing-modulated odor responses are already present at the first relay of the ascending olfactory pathway. This can be logically explained if OSN activity is contingent on the amount of odor molecules carried by the nasal airflow during breathing. While this may be expected, the high-degree of correlation in the phase-dependence of OSN responses and the behavioral weights captured by the logistic regression analysis (Fig. 2D) suggests that decision-making centers in the brain do not compensate for weaker ‘exhalatory’ inputs, remaining limited by the constraints imposed by breathing. Our results are related to previous reports on mice discriminating the sub-sniff timing and intensity of brief optogenetic stimulation of OSNs^24,58^, and confirm that variations in the amplitude of OSN responses elicited by differences in pulse timing within breathing cycle can also be differentially read and integrated by downstream areas for decision-making. MTC responses to odors have been shown to tile the breathing cycle^20,23,57^, but OSN responses in our experiments have fairly homogeneous breathing phase dependence. This difference suggests that circuits within the OB, likely the abundant and diverse types of inhibitory neurons^59^, shape the OSN inputs into MTC responses with different phase preferences. Our results also offer a basis for MTCs being able to track changes in the frequency of olfactory stimuli^22,28,29^. Lastly, there have been descriptions of changes in the amplitude of MTCs responses as a consequence of variations in breathing frequency^21,26^, which could partly arise from frequency-dependent variation in OSN responses.

We would like to note that there are some caveats that may have to be considered before generalizing the scope of these results. First, similar to previous work using head-fixed behavioral setups, mice displayed a limited range of respiratory frequencies while performing the task (mostly 2.5–5 Hz, Supplementary Fig. 2A, B). The current bandwidth is in the lower end of the range displayed during active exploration or trail following^15,60,61^. In addition, trained animals did not show any variations in the breathing frequency upon odor pulse presentation (Supplementary Fig. 2B) probably as a result of familiarization with the odorant. This contrasts with previous results revealing dynamic changes in breathing after presentation of novel odors^62,63^. Finally, the decrease over learning in the dominant frequency of breathing prevents us from ruling out the possibility that mice learn to adjust their breathing frequency to the odor pulse delivery rates they experienced. Altogether, it is unclear whether the dependence of perceptual weighing (or OSN activity levels) of intermittent stimuli with the breathing cycle would be similar during high-frequency breathing, or the sampling of novel or unexpected odor cues.

The dependence of the amplitude of glomerular responses on the timing of odor pulses relative to breathing can be helpful to understand how olfactory inputs are weighed when animals need to integrate information across multiple breaths. To exclude any effects of sensory adaptation in OSN responses, we focused on changes in GCaMP3 signal for odor pulses that were relatively temporally isolated (time between pulses > 1 s). Therefore, how OSNs respond to multiple pulses within a single breath remains to be explored in future work. Experiments in anesthetized, tracheotomized rats have shown that MTC responses to fluctuating odor stimuli scale linearly with stimulus duration, but non-linearly with stimulus concentration^57^. Moreover, recent work both using calcium imaging and in vivo electrophysiological recordings has suggested that MTC populations across glomeruli seem to be correlated with odor plume temporal dynamics^30,31^. Recent work tracking OSN responses and behavior to fluctuating odor stimuli have revealed that glomerular responses to different levels of intermittency are heterogeneous, and that heterogeneity is necessary for better predictions of intermittency^32^. We note that the current work likely used odorant concentrations above what can be found in natural environments^64^. Consequently, the number of glomeruli activated by odor pulse presentation is likely higher than what a naturalistic intermittent plume would trigger. However, it is important to emphasize that all activated glomeruli shared the same phase-dependence in the response, irrespective of their concentration sensitivity (Supplementary Fig. 4). Altogether, our results provide functional evidence of the integration of olfactory stimuli over time for odor plume categorization, although complementary experiments are still required to determine which aspect of odor plume dynamics glomeruli track and what the underlying neural mechanisms are.

### Piriform cortex encoding of fluctuating stimuli

We have characterized, for the first time, the response of APCx neurons to highly fluctuating olfactory stimuli. Odors presented passively or within a behavioral task are shown to be represented by not-so-sparse neuronal ensembles in APCx^65–68^. These studies typically used longer and non-fluctuating odor stimuli and mostly focused on characterizing the size of the ensembles required for reliable odor identity representation. Our results show that when the same odorant is delivered as brief pulses, different subpopulations within the ensemble are recruited each time in a stochastic fashion. In trials in which multiple odor pulses were delivered, timing between the first and second pulse determined the neuronal response to the second pulse, providing clear evidence of sensory adaptation in APCx responses, which could partly account for the variable responses of neurons to each odor pulse. Another source of variability is the differential tuning of APCx neuron response to the timing of odor pulses within the breathing cycle. We found that roughly 50% of the APCx population responded equally to odor pulses falling at any time during the breathing cycle, whereas the remaining 50% may be tuned to different pulse timings over the breathing cycle. It is important to note that neuronal response amplitudes were estimated by the DUNL method by grouping spikes in 50 ms bins, which may influence inferences about the sparseness and phase-tuning of the responses.

What are the signals that shape the phase-tuned odor responses in APCx? One possibility is that a subpopulation of APCx neurons may inherit the tuning from their MTC inputs. Recurrent inhibitory signals in the APCx appear to be essential in controlling the timing and gain of APCx population responses to odors^66^, and may contribute to phase tuning observed in our experiments. Moreover, a recent report has shown that, even in the absence of odors, inhalation can trigger bidirectional changes in the firing rates of APCx neurons, driven mostly by feedforward bulbar input, but with a small top-down component^27^. Mice decreased their dominant breathing frequency across task training (Supplementary Fig. 2A). In this scenario, it becomes unclear if the phase clusters observed are innately present in the APCx population or if they emerge over the course of learning. Future experiments can test these alternatives, as well as assessing whether the functional differences observed across clusters are correlated with differences in molecular identity or connectivity of APCx neurons.

Do olfactory inputs trigger similar response profiles across the APCx neuronal population? The neuron-specific response kernels revealed that the APCx population is also heterogeneous in the time course of their responses to olfactory inputs. It is possible that different kernel clusters may correspond to different cell-types within the APCx. APCx harbors a molecularly and anatomically diverse population of excitatory cells^41,69^, and also two subpopulations of inhibitory cells which have been shown to engage in local feed-forward and feed-back inhibitory circuits^66^. In fact, Bolding and Franks (2018) described how those inhibitory circuits were essential for silencing APCx population responses after a peak firing during the first sniff. Interestingly, we did not find a correspondence between the clusters defined by the kernels and those defined by the phase-tuning, suggesting that APCx neurons with a specific phase-tuning can diverge in terms of their response profile and vice-versa. However, we take the kernel-based clusters as a global snapshot of the functional diversity within the APCx, and their correspondence with neuronal clusters defined by gene-expression or projection patterns will need to be clarified with future experiments.

### Sensory vs. decision information in piriform cortex

We found that task-related variables could be decoded from a small number of APCx neurons (~20) when the entire sampling window was considered. This might not be surprising if we take into account that subpopulations of ~100 randomly sampled APCx could accurately classify different odor identities^65,67,70^. In those previous reports as well as in the current work, we found that further increasing the number of neurons did not improve classification accuracy, supporting the idea of high redundancy in the encoding of olfactory inputs at the APCx. Higher classification accuracies were found for trial identity compared to choice, providing the first indication that APCx population firing might be reflecting the time course of sensory inputs, rather than any sort of neural correlate of evidence accumulation. This idea gets additional support after finding the classification accuracy for APCx neuronal firing does not significantly increase over time for independent time intervals spanning the sampling window. Moreover, previous studies in other sensory modalities have shown that despite reliably responding to sensory inputs, and the impairments in performance after their inactivation, early sensory cortices may not be responsible for sensory evidence accumulation^71–74^. The fact that the amount of sensory input is unbalanced between the two trial types poses an additional caveat for the interpretation of these results. In the future, perturbation experiments, as well as behavioral assays in which the integrated olfactory signal could be balanced between the two trial types in the current task - for instance, computing the ratio of pulses between two different odorants - will provide further clarification about the role of APCx in the encoding of olfactory statistics. Different clusters of APCx neurons achieved different decoding accuracies, but whether the most accurate neurons have specific projection patterns to non-olfactory areas that might be involved in decision making remains to be seen.

Our study leaves open the question of where in the mouse brain olfactory statistics are accumulated for decision-making during the task. APCx sends both extensive projections to other olfactory areas, and also direct projections to the orbitofrontal cortex, medial prefrontal cortex, and medial dorsal thalamus^75^. Extensive work in mice assessing accumulation of visual or auditory evidence has identified areas in the parietal and prefrontal cortex responsible for the graded accumulation of sensory inputs and the consequent categorization of the accumulated value into a behavioral decision^43,73,74,76^. It might be likely, then, that olfactory evidence could be also accumulated in those areas. Additionally, recent rodent work has shown the involvement of the anterior dorsal striatum in auditory evidence accumulation^77^, a relevant finding if we consider that olfactory inputs can reach the ventral striatum through the olfactory tubercle^78–80^, an area associated with the encoding of odor valence but with direct connections from OB and APCx. If the temporal integration of olfactory stimuli plays a role in naturalistic behaviors such as trail following, which may also involve multimodal integration, it is likely that similar brain hubs for sensory evidence accumulation are engaged across different sensory modalities.

## Methods

### Experimental animals

All experimental animals used for behavior and electrophysiology were C57Bl6/J mice of either sex acquired from Jackson Laboratories, aged two to four months at the start of experiments. Following the implantation of a tetrode drive and/or head plate, all mice were housed individually. Behavior and physiology experiments took place over the course of one to two months. After the completion of all experiments, mice were euthanized, and post-mortem histology was performed to confirm the location of electrophysiological recordings. Adult heterozygous OMP-GCaMP3^81^ from a breeding stock maintained within Harvard University’s Biological Research Infrastructure were used for the olfactory bulb imaging. All mice used in this study were housed in an inverted 12 h light cycle and fed ad libitum. Animals were housed at 22  ±  1 °C at 30–70% humidity. All the experiments were performed following the guidelines set by the National Institutes of Health and approved by the Institutional Animal Care and Use Committee at Harvard University.

### Behavioral apparatus

A custom-made apparatus was built inside a Faraday cage to allow the execution of behavioral experiments simultaneously with electrophysiology and imaging. Animals were head-fixed over a styrofoam ball which allowed them to freely walk during behavioral sessions. A pair of lick ports were appropriately positioned in front of the animal through a 3D printed platform coupled to a stepping motor controlled from a TinyG v8 microcontroller (Synthetos). Water delivery was executed from an Arduino-Mega (Arduino), controlling two 3-way solenoid valves (LHDA12333115H, Lee Company). Licking detection was achieved through a capacitive circuit.

Odor pulse delivery was accomplished using a custom olfactometer equipped with fast proportional solenoid valves (EV-P-05-09-A0; Clippard) controlled from a Teensy 3.2 (PJRC). The fast kinetics of the solenoid valves used allowed us to switch between constant clean air delivery and 50 ms activations of another solenoid valve connected to a vial containing a diluted monomolecular odorant. 5% (v/v) ethyl valerate (Sigma-Aldrich) was used for all the experiments, except Supplementary Fig. 1E that shows the psychometric curves for a separate cohort trained with 5% (v/v) methyl butyrate (Sigma-Aldrich). The resulting odor stream was sent directly to a 3D printed mask fitted to the snout of the animal. For behavior and electrophysiology experiments, ethyl valerate (or methyl butyrate) dilutions were done in mineral oil (Sigma-Aldrich), whereas for imaging experiments they were done using di-ethyl-phthalate as solvent (Sigma-Aldrich). Catch trials were introduced by delivering 50 ms odor pulses from a separate valve connected to a clean air vial. The mask was also connected to an airflow sensor (AWM3100V; Honeywell) to monitor animal breathing during the task, as well as to a vacuum line to ensure quick removal of the odorized air from the snout of the mouse. Generally, a constant airflow of 1 L/min was kept during the experiment, but we identified small transients of 25 sccm during odor pulse delivery. Vacuum flow from the mask, as well as the airflow input to the olfactometer, were regulated using two different mass-flow controllers (QPV1, Proportion Air). Photoionization detector (PID; Aurora Scientific) measurements were performed in absence of any animals but in the same conditions of an experimental session, with the probe of the PID placed at the approximate position of the nostrils of the mouse.

Global task structure and communication with the different microcontrollers and cameras was achieved using custom-written software in Python. Behavioral data (licking and breathing) was acquired using a PCIe-6351 card (National Instruments).

### Behavioral task

Task structure was controlled by custom-written software in Python. Each trial in the Poisson-distributed Odor Pulses Task comprised three different periods (Fig. 1). The first one – “Wait Period” – had a 5 s duration and its ending was marked by a 10 kHz sound cue. The second period - “Sampling Period”- could have different durations across different training and testing sessions (see Behavioral Training below) and was the time window during which odor pulses were delivered and lick ports were moved away from the animal by a motor. A 6 kHz sound cue indicated the end of the “Sampling Period” and the displacement of the lick ports closer to the animal, leading to a 1.5 s “Go Period” in which the animal had to report its decision by licking one of the lick ports according to the total pulse count. If the animal made the correct licking choice, a 7 µL water drop was delivered, whereas if the lick was in the wrong lick port no water was delivered, a 3.8 kHz buzz tone was presented, and a 10 s timeout was given. A regular behavioral session consisted of 100–200 trials. Catch trials were presented in a separate experiment with a cohort of 3 animals and represented 10% of the total trials in a session.

### Behavioral training

One week after surgery, mice were habituated to be handled and head-fixed by experimenters. Next, animals were water restricted in compliance with approved protocols. On the first day of water restriction, mice were given 20 min of free exploration of the behavioral apparatus with water available from lick ports. In the next session, animals were head-fixed in the dark, and water drops were delivered frequently to encourage them to find the lick ports. Then, animals were moved to the ‘Lick Training’ phase, in which they were presented for the first time with the trial structure, but without delivering any odor pulses. During this phase, water was automatically delivered to either of the two lick ports after the second sound cue to encourage animals to lick from the lick ports to get the reward. Lick port location adjustments and manual delivery of water to neglected lick ports were done -if needed- to avoid the development of biases towards a specific side. Once animals showed commitment to lick until they were satiated, they started the actual training in the behavioral task. A summary of the stages of the training for an example animal is provided in Supplementary Fig. 1B. Initially, mice started in the ‘Block training’ phase: actual task structure was introduced, with odor pulses being delivered during a 1 s sampling window. At this point, the total number of pulses in a trial could be drawn from two different Poisson distributions with different mean values: *λ*_1_ = 16 for ‘High Trials’ and *λ*_2_ = 1 for ‘Low Trials’, with both types of trials presented in blocks of 17 trials. Also, for the first 1–2 sessions of ‘Block Training’, water was delivered automatically to the rewarded side to help the animal to learn the association between the number of pulses and the side of the reward. After the second session in this phase, water was only delivered upon the animal licking in the side associated with reward for that type of trial. Block size was progressively reduced after animals reached more than 80% success in a session until the two types of trials were delivered randomly. Then the behavioral shaping phase started: while keeping the random trial structure, the difference between *λ*_1_ and *λ*_2_ was progressively reduced, and the sampling window duration was increased. The whole training process, including training in the initial binary classification and later shaping, took 4–6 weeks.

### Olfactory bulb craniotomy

A craniotomy was performed to provide optical access to both olfactory bulbs. Mice were first anesthetized with an intraperitoneal injection of ketamine and xylazine (160 and 16 mg/kg, respectively) and the eyes were covered with petroleum jelly to keep them lubricated. Before starting the surgery, carprofen (7.5 mg/kg) and dexamethasone (10 mg/kg) were injected subcutaneously, followed by an intramuscular injection of cefazolin (500 mg/kg). Body temperature was maintained at 37 °C by a heating pad. The scalp was shaved and then opened with a scalpel blade. After thorough cleaning and drying, the exposed skull was gently scratched with a blade, and a titanium custom-made headplate was glued on the scratches with Loctite 404 Quick Set Adhesive. The cranial bones over the OBs were then removed using a 3 mm diameter biopsy punch (Integra Miltex) and then the brain surface was cleared of debris. The exposed brain area was kept moist with artificial cerebrospinal fluid containing (in mM) 125 NaCl, 5 KCl, 10 Glucose, 10 HEPES, 2 CaCl_2_ and 2 MgSO_4_ [pH 7.4], as well as Gelfoam soaked in the same solution (Patterson Veterinary). Two 3 mm No. 0 glass coverslips (Warner) were glued together with optical adhesive (Norland Optical Adhesive 61) and adhered to the edges of the vacated cavity in the skull with Vetbond (3 M). C&B-Metabond dental cement (Parkell, Inc.) was used to cover the headplate and form a well around the cranial window. After surgery, mice were injected with slow-release buprenorphine (3.25 mg/kg). Animals were allowed to recover for at least 7 days.

### Wide-field calcium imaging

Wide-field imaging was performed on adult OMP-GCaMP3 mice as described previously^82^ using a 4X objective (Olympus, NA 0.1). Blue light from an LED (M470L3, Thorlabs) was used for excitation, and the emitted light was filtered (MF525-39, Thorlabs) and collected with a CMOS camera (BFLY-U3-23S6M-C, FLIR). Videos were acquired using a 10 Hz frame rate and a 2 × 2 binning of the image.

### Chronic tetrode implantation

Mice were anesthetized with an intraperitoneal injection of ketamine and xylazine (100 mg/kg and 10 mg/kg, respectively), then placed in a stereotaxic apparatus. After the skull was cleaned and gently scratched, a custom-made titanium head bar was glued to it. A small craniotomy was performed above the implantation site, before 8 custom-built tetrodes (Chang et al., 2013)^83^ were lowered together into the brain (coordinates for APC: antero-posterior 1.6 mm, medio-lateral −2.8 mm, dorso-ventral 3.4 mm; all antero-posterior and medio-lateral coordinates are given relative to Bregma; all dorso-ventral coordinates are given relative to the brain surface). A reference electrode was implanted on the occipital crest. All the mice were implanted in the right hemisphere. The whole system was stabilized with dental cement. Mice were given a week of recovery before any new manipulation.

### Neuronal recordings

A week after chronic tetrode implantation, mice were habituated and trained in the task as described above. Brain activity was recorded once a day, always at the same period of the day and only once mice reached proficiency in the task. Recordings were conducted while animals were awake and head-restrained, and data was only collected during the 5 s ‘Sampling Window’ period of each trial. However, the first 500 ms of the recordings were discarded due to artifacts. The tetrodes were slightly lowered in the brain after each recording session (around 40 µm deep), ensuring that different neurons were recorded each day. Electrical activity was amplified, filtered (0.3–6 kHz), and digitized at 30 kHz (Intan Technologies, RHD2132, connected to an Open Ephys board). Single units were sorted offline manually using Kilosort v1^84^. Units with more than 1% of their inter-spike intervals below 2 ms refractory period were discarded. Units displaying large changes of amplitude or waveform during the recording were also discarded^85^. The position of the tetrodes in the brain was confirmed post-mortem through electrolesion (200 μA for 4 s per channel).

### Noise modeling

The modeling of how the total number of pulses delivered, *N*_*p*_, contributes to the decision noise was inspired by a previous report^33^. Our model assumed that the decision-making process of a mouse in each trial starts with the estimation of the total number of pulses delivered (*N*_est_), followed by the comparison of this value to the estimation of the threshold for trial categorization (*N*_*b*_).

As is shown in Eq. 1, we assumed *N*_*est*_ followed a normal distribution centered around *N*_*p*_, with a variance *σ*^*2*^_*N*_, which constitutes the first source of decision noise and reflects the noise in the estimation of the total number of pulses.

1 $${N}_{{est}}{{ \sim }}{{\mathscr{N}}}({N}_{p},{\sigma }_{N}^{2})$$

The second source of noise arises from the internal estimation of the *N*_*b*_, a parameter that we also assumed follows a normal distribution with constant variance *σ*^*2*^_*b*_ for all *N*_*p*_. Then, as it is shown in Eq. 2, we propose that licking decision on a trial is based on ΔN, i.e., the internal representation that the animal has of the difference between sensed pulses (*N*_*est*_) and the decision boundary (*N*_*b*_).

2 $$\varDelta N{{ \sim }}{{\mathscr{N}}}({N}_{{est}}-{N}_{b},{\sigma }_{N}^{2}+{\sigma }_{b}^{2})$$

Therefore, the probability of mouse choosing the ‘High’ side is equal to the probability of ΔN being greater than 0, which corresponds to the area under the Gaussian curve defined by Eq. 2:

3 $$P({{\rm{choice}}}=^{\prime} {{\rm{High}}}^{\prime} {|N}b,\sigma 2b,\sigma 2N)=P(\varDelta N > 0)=	\int\limits^{{\infty }}_{0}{{\mathscr{N}}}({N}_{p}-{N}_{b},{\sigma }_{N}^{2}\\ 	+{\sigma }_{b}^{2})d({N}_{p}-{N}_{b})$$

and simplifies to:

4 $${{\rm{P}}}({{\rm{choice}}}=^{\prime} {{\rm{High}}}^{\prime} |{{\rm{N}}}{{\rm{b}}},{{\rm{\sigma }}}2{{\rm{b}}},{{\rm{\sigma }}}2{{\rm{N}}})=\frac{1}{2}\left[1+{{\rm{erf}}}\left(\frac{{N}_{p}-{N}_{b}}{\sqrt{2\left({\sigma }_{N}^{2}+{\sigma }_{b}^{2}\right)}}\right)\right]$$

Erf refers to the error function used to calculate the area under the normal distribution that reflects the probability of choosing ‘High’. Fitting the model with the values of *N*_*p*_ and the mouse choice in each trial, we can obtain the maximum likelihood estimation of the parameters. Confidence intervals of parameters were estimated through bootstrapping 20,000 trials with replacement over 1000 iterations.

The likelihood function can be described with a total of 22 parameters (*N*_*b*_, *σ*_*b*_ and 20 *σ*_*N*_’s corresponding to distinct numbers of pulses). Note however, that since the variances appear as ${\sigma }_{N}^{2}+{\sigma }_{b}^{2}$, we will define $\bar{{\sigma }_{N}}=\sqrt{{\sigma }_{N}^{2}+{\sigma }_{b}^{2}}$ and find these values instead. Further, note that *N*_*b*_ should correspond to the point where the psychometric probability crosses 0.5. However, because of the discrete nature of *N*_*p*_’s, *N*_*b*_ could be determined up to an interval between two integers. We choose *N*_*b*_ to be the closest integer to the point of 0.5-crossing, which in our data leads to *N*_*b*_ = 8. This leaves us with 20 $\bar{{\sigma }_{N}}$ parameters to be calculated from MLE.

For testing the different models of noise scaling to the behavioral data, maximum likelihood estimation of the specific parameters of each of the models -and their correspondent confidence intervals- was performed bootstrapping 15,000 trials with replacement over 1000 iterations.

### Mixed-models logistic regression

Odor pulses in every trial were convolved with a kernel derived from PID recordings, and odor signal was binned and averaged for every bin. Then, the contribution of odor information to mouse’s licking decision was assessed by fitting two different mixed effects logistic regression models using Pymer4 package^86^. The first model was fitted specifying a single, shared coefficient for all bins of odor information, whereas the second model was fitted allowing each bin of odor information to have different coefficients. Therefore, the models represent scenarios in which odor information is weighed equally or differentially across the whole ‘Sampling Period’. Models were compared using a log-likelihood ratio test.

### Perceptual weight estimation through binary logistic regression

For each trial, the breathing signal was split into individual breaths, and each breath was divided into 15 bins. Therefore, the length of each bin could vary from one breath to the other depending on the length of each breath, but each bin represented a specific phase of the breathing cycle across multiple bins. Next, each odor pulse was assigned to a specific phase bin in the breath which arrived, and a phase histogram was computed for each trial compiling the number of pulses that arrived at each of the 15 phase bins. Subsequently, the binary logistic regression model was fitted for each trial following Eq. 5 below:

5 $$y \sim \sigma \left({\sum }_{n=1}^{n=15}{x}_{n}{w}_{n}\right)$$

Where *y* is the binary choice (0 = ‘low’ side, 1=’high’ side) done by the mouse in that trial, *x*_*n*_ represents the number of pulses in bin *n*, *w*_*n*_ corresponds to the weight associated with that specific bin, and *σ* indicates the logistic function. Regression coefficients were estimated using bootstrapping (1000 iterations, sampling 15,000 trials in each iteration and then fitting the model). Figure 2D depicts the values of the *w*_*n*_ for each of the phase bins.

Next, effective pulse counts could be calculated by computing the dot product between the phase histogram (i.e., a vector indicating how many pulses occurred at each of the 15 phase bins) and the vector of weights plotted in Fig. 2D.

### Analysis of APCx spiking data

For each behavioral session, spikes from the entire APCx neural population were used to fit a linear regression model aimed at predicting the total number of odor pulses delivered on a trial. Neurons were sorted based on the *p*-values obtained from the regression and new linear regression models were fitted excluding neurons one by one. Next, the different regression models (each containing spikes from different subsets of neurons) were compared using the Akaike Information Criterion (AIC). AIC is an objective statistical tool used for comparing and selecting the best-fitting model from a set of candidate models. It takes into account goodness-of-fit but also adds penalties for the number of parameters (individual neurons, in this case). Then, only those neurons whose spikes led to a reduction in the AIC of the regression model fitted for every session were considered for all subsequent analysis (except for the dependence on the decoding with the size of population in Fig. 4F–H).

In particular, for the decoding analysis, a linear regression model was fitted to predict total odor pulse counts based on the spiking of the selected neurons in the APCx population, whereas logistic regression models were used to predict variables with binary outcomes, such as trial identity (‘High’ or ‘Low’) or mouse choice (‘High side’ or ‘Low side’). To test whether APCx neurons were accumulating evidence for decision-making, APCx firing during odor pulse presentation was divided into different intervals, and regression models were ran using the spikes in cumulative or independent intervals.

#### Analysis of APCx neural responses using deconvolutional unrolled neural learning (DUNL)

Single-trial neural activity was decomposed into impulse-like responses to odor pulse presentation as it has been described in a previous report^45^. DUNL modeled the neural spikes with a Poisson distribution, whose rates in a given trial was expressed as the convolution of a kernel characterizing the response of the neuron to an odor pulse with sparse codes (a vector representing the timing of events), together with the strength of the neural responses associated with those events, complemented by a baseline trial activity. DUNL used a bin size of 50 (i.e., for learning a 1 s kernel at 1 ms resolution data, the kernel has 20 samples). Kernels were learned fully from data, and in this dataset a single kernel was learnt for each APCx neuron. DUNL has knowledge of the code support, i.e., non-zero elements of the sparse code were aligned to the timing of the odor pulses, and the encoder was unrolled for 100 iterations (with step size of 1) using ReLU activation functions to impose additional sparsity on the sparse codes. The baseline trial activity is computed using the average spike counts scaled by the number of occurred events (odors), passed through the link function. Stochasticity in the estimated activity was added by passing the convolved signal through a generative model using a Poisson process. The kernel characterization is achieved per neuron in two stages: (i) given the known timing (support), DUNL is trained with zero bias (no L1-based sparse) regularization; (ii) DUNL is further fine-tuned with additional L1-regularization sparsity and a smoothness regularization on the kernel. We used smooth regularization of 0.1 for all neurons, and the L1 code regularization is fine-tuned based on the best fit (KS and R2) within the set [0, 0.005, …, 1, 2, 3, 4, 5]. This additional code sparsity is applied to account for the fact that neurons may not always respond to an odor. DUNL is trained with Adam optimizer (lr = 0.01, and eps = 0.001) where kernels are initialized from alignment of responses at the odors onset (regardless of overlap). For convenience, we are renaming the code amplitudes as ‘response amplitudes’ in Fig. 4. Analysis on response amplitudes and kernels from APCx neurons was only performed in those neurons that were considered for the analysis of the spiking data. ‘Phase clusters’ were obtained by performing K-means clustering on the response amplitudes elicited by pulses at different times during respiratory phase in each neuron. ‘Kernel clusters’ were obtained after performing K-means clustering on the 1 s kernels learned for each neuron.

#### Analysis of imaging data

Images were processed using both custom and available MATLAB (MathWorks) scripts. Motion artifact compensation and denoising were done using NoRMCorre^87^. The CaIMaN CNMF pipeline^88^ was used to select and demix ROIs. dF/F values were calculated using the mean of a baseline period of at least 20 frames. To identify glomeruli that were significantly odorant-modulated, three points centered on the peak dF/F signal after odorant delivery were averaged. Glomeruli were classified as significantly odorant-modulated if their peak averaged response exceeded 2.5 standard deviations of the baseline noise. The dF/F signal for each odor-modulated glomerulus was normalized to the 99.5th percentile across the entire session. Estimation of the dependence of OSN calcium signals with odor pulse arrival time within the respiratory cycle was achieved by first selecting odor pulses that were isolated by at least 1 s both before and after their occurrence and then extracting the calcium signals around those peaks.

### Reporting summary

Further information on research design is available in the Nature Portfolio Reporting Summary linked to this article.

## Supplementary information

Supplementary Information

Reporting Summary

Transparent Peer Review file

## Source data

Source Data

## Data Availability

The data generated in this study have been deposited in Figshare under accession code 10.6084/m9.figshare.31792705. Additionally, source data are provided with this paper in the Source Data File. Source data are provided with this paper.

## Code availability

Code for the analysis of the data is provided at: https://github.com/leboero/Odor_Pulses_Task_Analysis/tree/main/NComms_Submission.
