# Supplementary material for: Perception and neural representation of intermittent odor stimuli in mice
Source: Nat Commun. 2026 Apr 29;17:5575. doi: 10.1038/s41467-026-72445-1 (PMC13304163; doi:10.1038/s41467-026-72445-1)
Supplement: Supplementary file 1 — Supplementary Information [file 41467_2026_72445_MOESM1_ESM.pdf]

Title: Perception and neural representation of intermittent odor stimuli in mice

Supplementary Figures

Supplementary Figure 1

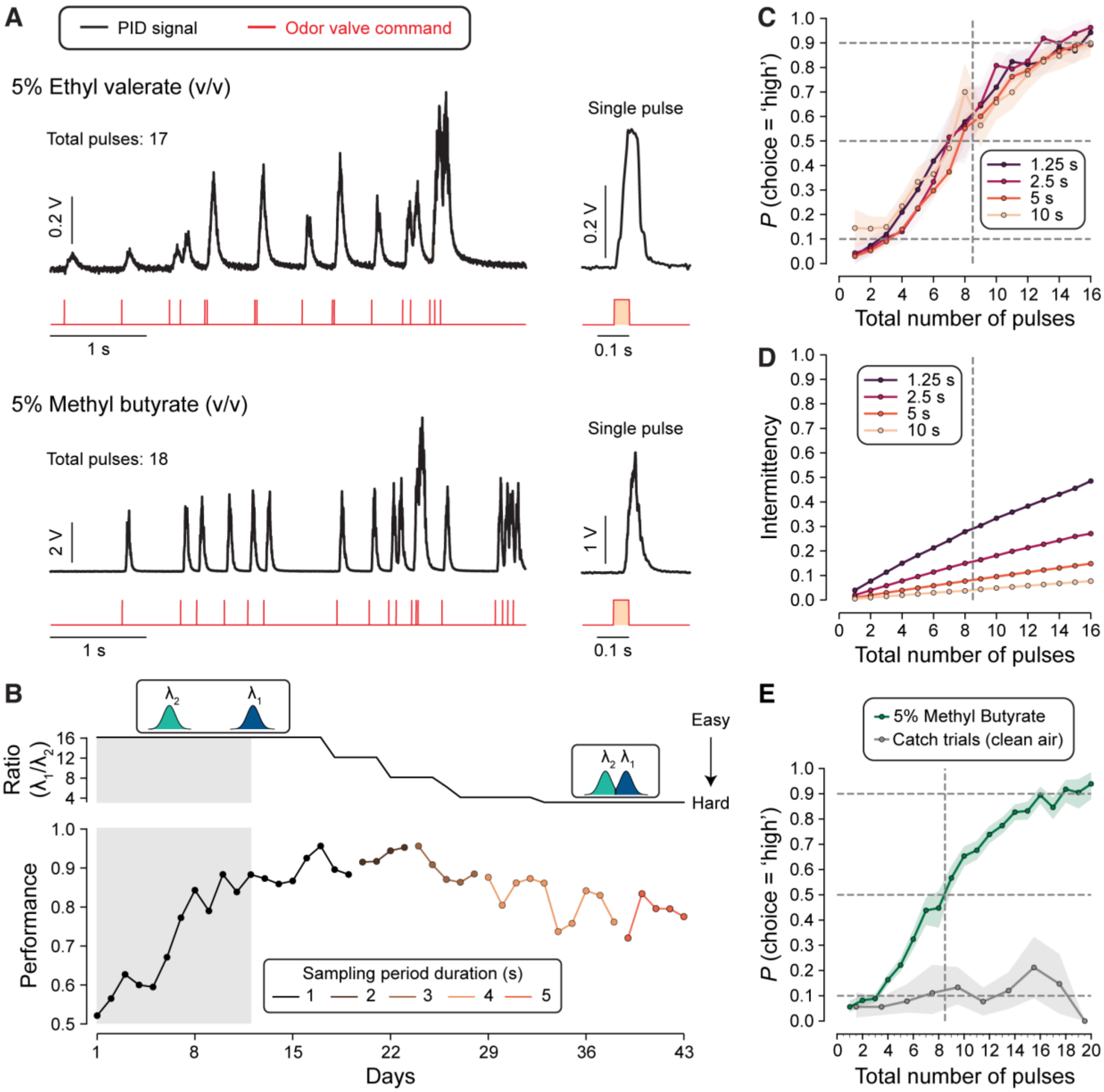

(Figure Caption on the next page)

Olfactometer characterization and task learning. (A) Left: PID recordings (black trace) after the delivery of a sequence of multiple 50 ms odor pulses (red trace, voltage command to the solenoid). Right: Example of the PID signal measured after a single 50 ms odor pulse. Top traces were obtained using 5% ethyl valerate (v/v) whereas bottom traces were obtained with 5% methyl butyrate (v/v). Temporal scale is the same for both PID and odor traces. An estimation of the odor signal in each trial could be generated by convolving the temporal course of voltage command to odor valves with this PID profile obtained from a single pulse. **(B)** Task learning for an example mouse. Top: The ratios (distance) between the two Poisson distributions controlling the total odor pulse counts in a trial. Bottom: Performance of the animal over the course of training. For details about how ratios and sampling windows durations were modified during the shaping phase please refer to Methods. Gray shaded area corresponds to training days with a trial structure having consecutive blocks of one type of trial. **(C)** Psychometric curves obtained with a $\lambda_1/\lambda_2 = 3$ but using different sampling window durations. The curve corresponding to the 5 s sampling window is the same as shown in Figure 1D. Sample sizes: 1.25 s (4 animals, 2760 trials), 2.5 s (4 animals, 2533 trials), 5 s (7 animals, 19991 trials), 10 s (4 animals, 2715 trials). **(D)** Intermittency of the odor signal for all total pulse counts in the conditions described in C. (**E**) Green trace: Psychometric curve obtained in a separate cohort of animals (n=3 mice, 12343 trials) using 50 ms odor pulses of 5% methyl butyrate in the same experimental conditions as Figure 1D. Grey Trace: Psychometric curve of the same animals for catch trials with clean air pulses delivered (690 trials). For catch trials, choice values were binned for consecutive pairs of odor pulses just for aesthetic purposes. For C-E, data is presented as mean $\pm$ 95% confidence interval.

29 **Supplementary Figure 2**

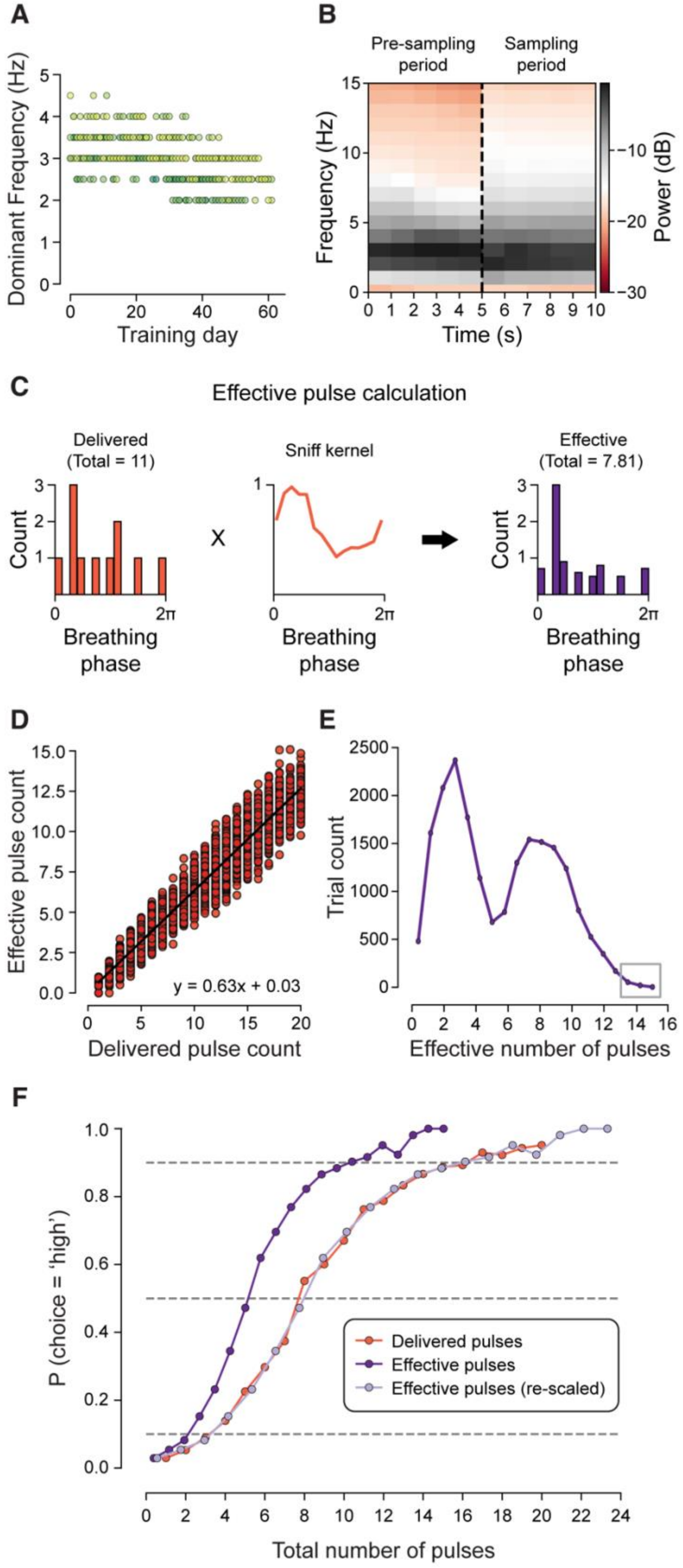

(Figure Caption on the next page)

Calculation of effective pulse counts based on pulse and breathing information. (A) Mean dominant frequency in mice breathing across training. Each color corresponds to a different mouse (n=7). (**B**) Average spectrogram of breathing for 5s trials across all mice. (**C**). For effective pulse calculation, the dot product between the phase histogram of pulses in a trial and the sniff kernel in Figure 2D was computed. (**D**) Regression between the delivered pulse counts in each trial and their corresponding effective pulse counts calculated by computing the dot product of the phase histograms and the coefficients from B. Each dot corresponds to a trial. **(E)** Histogram of number of trials across the whole range of effective pulse quantities. Since bins within the gray box had very low occurrence, they were pooled together with the closest bin (bin center = 12.72) for MLE calculations. **(F)** Comparison of the mean psychometric curve based in the total delivered pulse count (orange trace, same as in Figure 1D), the mean psychometric based in the effective pulse count -i.e. after convolving the phase histograms of each trial with the logistic regression coefficients from Figure 2D- (dark purple trace), and the mean psychometric curve based on effective pulse counts after re-scaling based on the linear regression shown in Supplementary Figure 2D (light purple trace). N = 19,896 trials across 7 mice.

Supplementary Figure 3

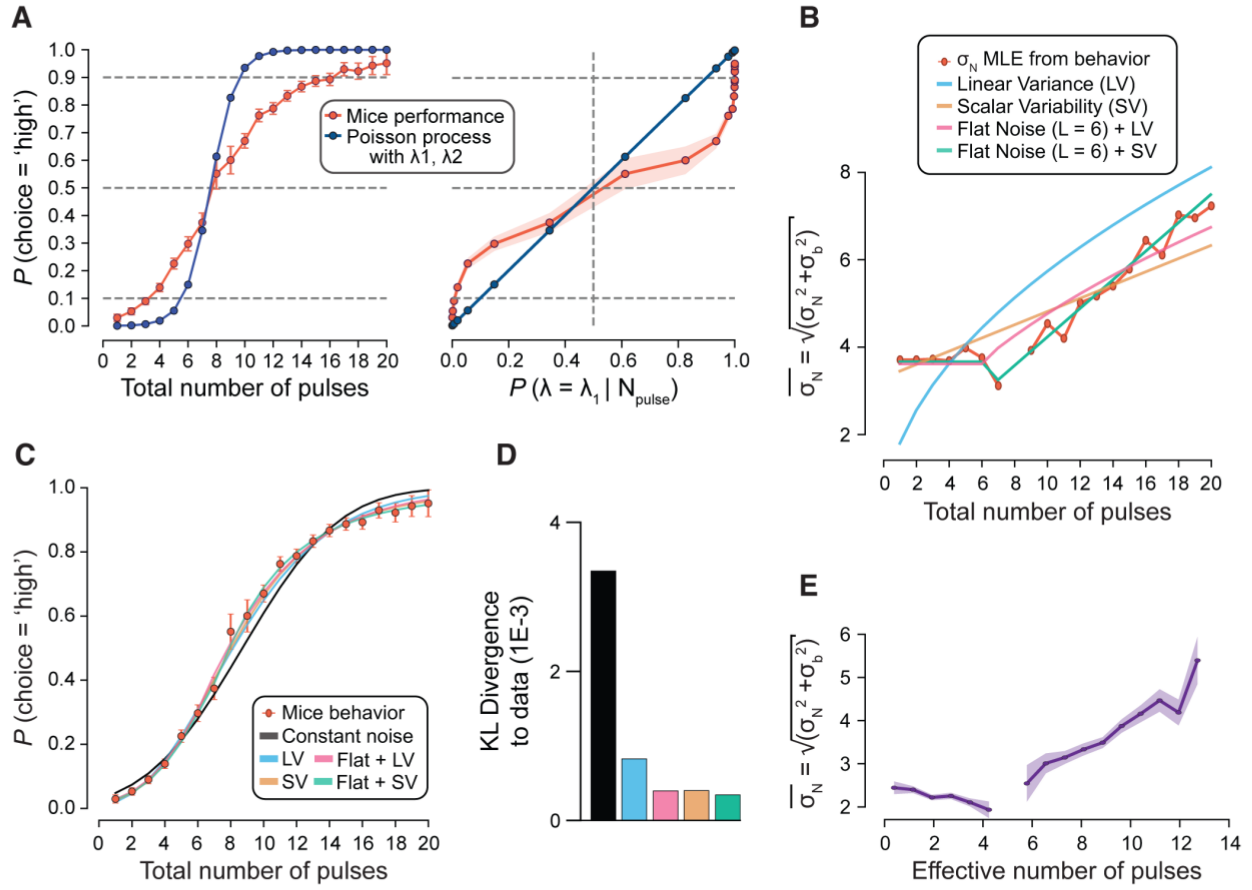

Decision noise scales with the number of odor pulses presented. **(A) Left**: Comparison of the psychometric curve obtained from mice choices (orange trace, same as the one in the bottom panel of Figure 1D) and the psychometric resulting from calculating the probability of binary choice assuming the animal is making the choice based on the ratio of conditional probabilities based on the known values of λ_1 and λ_2 for the two Poisson distributions generating the total pulse count on each trial (blue trace). The λ_1 and λ_2 values used were the same as those used for generating total pulse counts in a trial for the behavioral data. Mice data: mean + 95% CI. **Right**: Similar to B, the probability of choosing the 'High' side was computed as a function of the probabilities calculated based on the ratio of probabilities between the two Poisson distributions used for generating the total pulse counts on the trial. Mice choices do not follow the choice predictions based on the ratio of probabilities arising from the two Poisson distributions. (**B**) Mean bootstrapped MLE estimates of $\overline{\sigma_N}$ (same as in Figure 2G) and the predicted $\overline{\sigma_N}$ values according to the different models of noise scaling. Scalar variability (SV) models assume a linear scaling of noise with stimuli count, whereas linear variance (LV) models assume a square root scaling of noise with stimulus count. Flat noise models assume constant noise values for low pulse quantities until a limit, followed by some form of noise scaling -such as LV or SV. **(C)** Fits of different models of noise scaling with stimulus number to the observed psychometric curve (orange trace, same as in Figure 1D). For comparison, we also show fits of a model assuming a constant value of $\overline{\sigma_N}$ irrespective of the total number of stimuli presented ('Constant Noise'). (**D**) Kullback-Leibler (KL) divergence to data was computed for all models. The model with the best fit to data was the one assuming a flat noise followed by a linear scaling of noise with the number of stimuli (Flat Noise (L=6) + SV). **(E)** MLE estimation of $\overline{\sigma_N}$ across effective pulse count values without rescaling. Results are expressed as mean $\pm$ SD.

Supplementary Figure 4

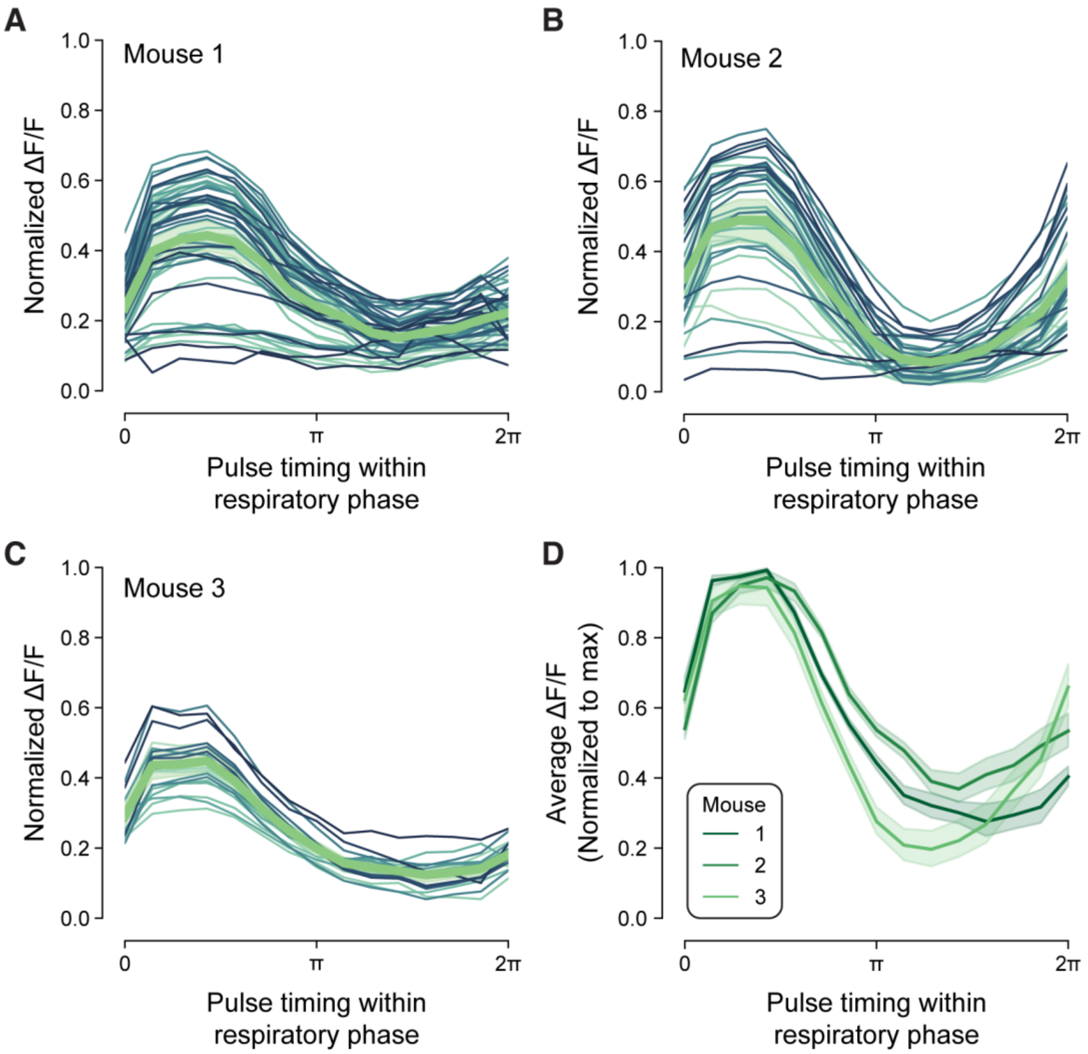

Phase-dependence of individual glomerular responses. (**A-C**) GCaMP3 fluorescence (ΔF/F normalized to the 99.5th percentile) as a function of odor pulse timing relative to the respiratory phase for the three different animals tested. Each thin trace corresponds to a single glomerular ROI (all ROIs that showed significant GCaMP3 response), whereas the thick green line is the average across ROIs. The dependence on pulse timing for response amplitude can be verified even at the level of single glomerulus. (**D**) GCaMP3 ΔF/F (normalized to the maximum value) as a function of odor pulse timing relative to the respiratory phase averaging across all ROIs in each animal. Data in this panel is expressed as mean ± CI 95% (n = 3 animals).

Supplementary Figure 5

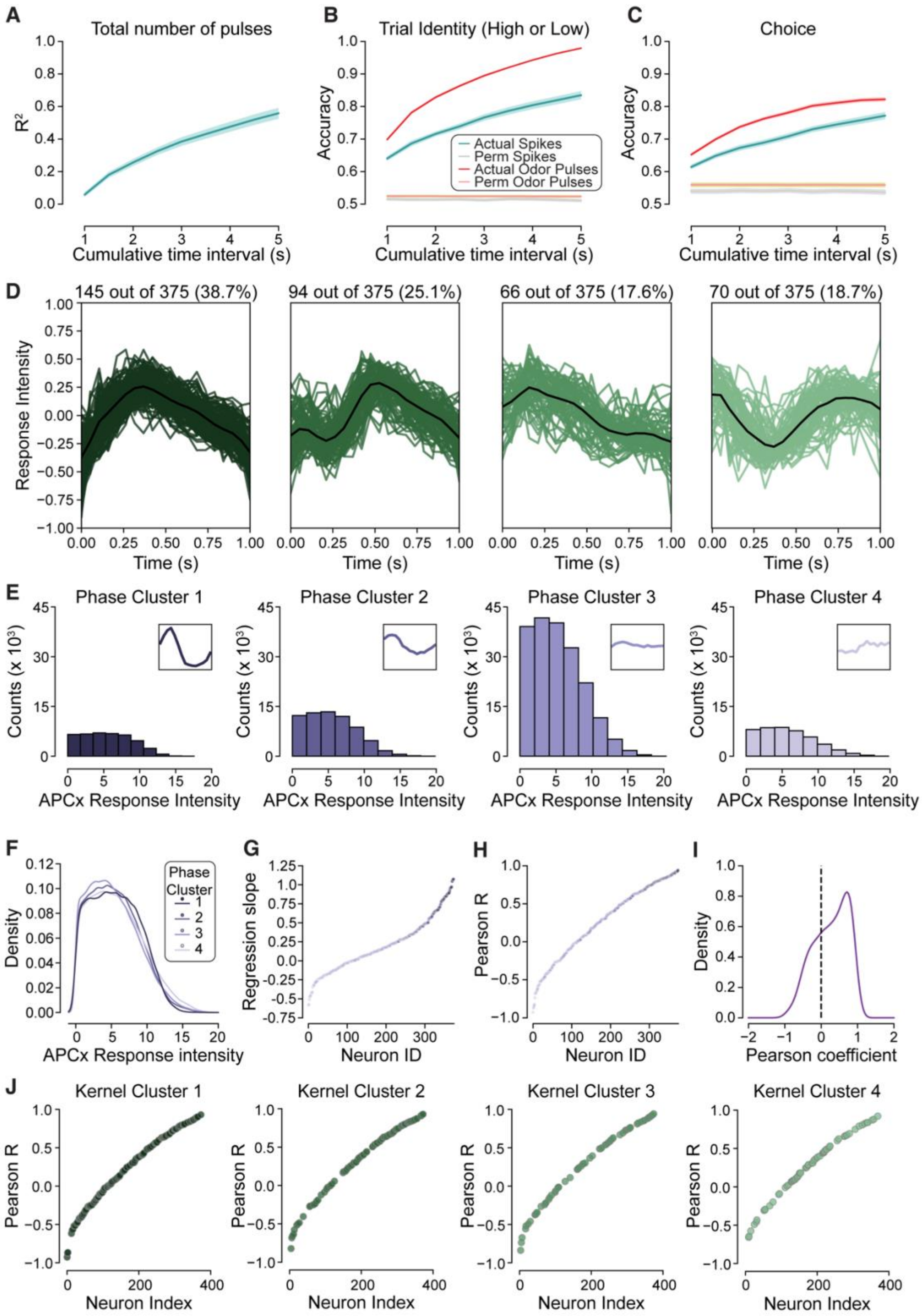

(Figure caption on the next page)

Heterogeneity in the phase-tuning and estimated response kernels of APCx neurons. (A) Average multiple linear regression R^2 using spike counts over cumulative 500 ms intervals to predict total pulse counts. **(B-C)** Teal traces depict prediction accuracy of logistic regression on trial identity **(B)** and behavioral choice **(C)** using population spike counts over cumulative 500 ms intervals. Gray traces are the accuracies obtained when target labels were permuted. Red traces show the decoding based on the odor pulse information available, whereas pink traces correspond to the accuracies after permutation of samples. **(D)** K-means clustering of the zero-mean kernels obtained for every neuron from DUNL. Colored lines: individual neurons. Black lines: average for each cluster. **(E)** Histogram of APCx response intensities for each phase cluster in Figure 5H-I (average cluster response profile shown in inset). **(F)** Kernel Density Estimations of APCx responses for each cluster. Jensen-Shannon divergences computed across pairs of phase cluster densities were all below 0.0072, suggesting that distributions were all nearly identical. **(G)** Sorted slopes of the linear regression between the response of every APCx neuron to pulses arriving at different times during the respiratory phase and the mean normalized OSN phase responses obtained from calcium imaging. Coloring indicates the phase cluster for each neuron. **(H)** Same as G, but for the Pearson coefficients of the correlation between the response of every APCx neuron and the mean OSN phase response from Figure 3C. **(I)** Kernel Density Estimation of the distribution of correlation coefficients in H. The distribution did not follow a normal distribution (Kolmogorov-Smirnov test, p-value = 1.21 E-23) and was skewed-left, supporting the existence of a significant group of neurons with high respiratory-tuning in their sensory-triggered responses **(J)** Neurons sorted using a similar approach as in H, but coloring based on the clusters of neuronal kernels in D. Data is shown as mean $\pm$ CI 95%, n = 3 animals (over 17, 13 and 12 behavioral sessions).
