# Supplementary material for: Perception and neural representation of intermittent odor stimuli in mice
Source: Nat Commun. 2026 Apr 29;17:5575. doi: 10.1038/s41467-026-72445-1 (PMC13304163; doi:10.1038/s41467-026-72445-1)
Supplement: Supplementary file 2 — Reporting Summary [file 41467_2026_72445_MOESM2_ESM.pdf]

Corresponding author(s): Double-anonymous peer review submissions: write DAPR and your manuscript number here instead of author names.

Last updated by author(s): YYYY-MM-DD

Reporting Summary

Nature Portfolio wishes to improve the reproducibility of the work that we publish. This form provides structure for consistency and transparency in reporting. For further information on Nature Portfolio policies, see our Editorial Policies and the Editorial Policy Checklist.

Statistics

For all statistical analyses, confirm that the following items are present in the figure legend, table legend, main text, or Methods section.

n/a	Confirmed	
☐	☒	The exact sample size (n) for each experimental group/condition, given as a discrete number and unit of measurement
☐	☒	A statement on whether measurements were taken from distinct samples or whether the same sample was measured repeatedly
☐	☒	The statistical test(s) used AND whether they are one- or two-sided<br>*Only common tests should be described solely by name; describe more complex techniques in the Methods section.*
☒	☐	A description of all covariates tested
☐	☒	A description of any assumptions or corrections, such as tests of normality and adjustment for multiple comparisons
☐	☒	A full description of the statistical parameters including central tendency (e.g. means) or other basic estimates (e.g. regression coefficient) AND variation (e.g. standard deviation) or associated estimates of uncertainty (e.g. confidence intervals)
☐	☒	For null hypothesis testing, the test statistic (e.g. F, t, r) with confidence intervals, effect sizes, degrees of freedom and P value noted<br>*Give P values as exact values whenever suitable.*
☒	☐	For Bayesian analysis, information on the choice of priors and Markov chain Monte Carlo settings
☐	☒	For hierarchical and complex designs, identification of the appropriate level for tests and full reporting of outcomes
☐	☒	Estimates of effect sizes (e.g. Cohen's d, Pearson's r), indicating how they were calculated

Our web collection on statistics for biologists contains articles on many of the points above.

Software and code

Policy information about availability of computer code

Data collection	Data was collected using PyCharm 2019 and 2024.3.5, as well as Visual Studio Code 1.109.4
Data analysis	Data was analyzed using Python 3.9 and Matlab R2021a. Code for the data analysis can be found at https://github.com/leboero/Odor_Pulses_Task_Analysis/tree/main/NComms_Submission

For manuscripts utilizing custom algorithms or software that are central to the research but not yet described in published literature, software must be made available to editors and reviewers. We strongly encourage code deposition in a community repository (e.g. GitHub). See the Nature Portfolio guidelines for submitting code & software for further information.

Data

Policy information about availability of data

All manuscripts must include a data availability statement. This statement should provide the following information, where applicable:

- Accession codes, unique identifiers, or web links for publicly available datasets
- A description of any restrictions on data availability
- For clinical datasets or third party data, please ensure that the statement adheres to our policy

The data generated in this study have been deposited in Figshare under accession code https://doi.org/10.6084/m9.figshare.31792705

Additionally, source data are provided with this paper in the Source Data File.

Research involving human participants, their data, or biological material

Policy information about studies with human participants or human data. See also policy information about sex, gender (identity/presentation), and sexual orientation and race, ethnicity and racism.

Reporting on sex and gender	Not applicable
Reporting on race, ethnicity, or other socially relevant groupings	Not applicable
Population characteristics	Not applicable
Recruitment	Not applicable
Ethics oversight	Not applicable

Note that full information on the approval of the study protocol must also be provided in the manuscript.

Field-specific reporting

Please select the one below that is the best fit for your research. If you are not sure, read the appropriate sections before making your selection.

☒ Life sciences ☐ Behavioural & social sciences ☐ Ecological, evolutionary & environmental sciences

For a reference copy of the document with all sections, see nature.com/documents/nr-reporting-summary-flat.pdf

Life sciences study design

All studies must disclose on these points even when the disclosure is negative.

Sample size	No sample size calculation was performed. For behavioral experiments, the sample size was 10 animals belonging to different cohorts. For imaging experiments, the sample size was 3 animals. For APCx recordings paired with behavior, the sample size was 3 animals (3 out of the 10 used for behavior) and electrophysiological recordings were performed over multiple behavioral sessions.
Data exclusions	No data was excluded from the analysis.
Replication	Animals from different experimental cohorts were trained in the task, to confirm that task learning was independent of animal cohort or specific experimental conditions in each cohort. Reward sites for each of the conditions were switched between animals, to control for any side bias during training.
Randomization	No randomization was applied since there was no division between experimental groups in the current study
Blinding	No blinding was necessary since there was no division between experimental groups in the current study

Reporting for specific materials, systems and methods

We require information from authors about some types of materials, experimental systems and methods used in many studies. Here, indicate whether each material, system or method listed is relevant to your study. If you are not sure if a list item applies to your research, read the appropriate section before selecting a response.

Materials & experimental systems

n/a	Involved in the study
☒	☐ Antibodies
☒	☐ Eukaryotic cell lines
☒	☐ Palaeontology and archaeology
☐	☒ Animals and other organisms
☒	☐ Clinical data
☒	☐ Dual use research of concern
☒	☐ Plants

Methods

n/a	Involved in the study
☒	☐ ChIP-seq
☒	☐ Flow cytometry
☒	☐ MRI-based neuroimaging

Animals and other research organisms

Policy information about studies involving animals; ARRIVE guidelines recommended for reporting animal research, and Sex and Gender in Research

Laboratory animals	Mus musculus, strain: C57BL/6J, RRID: IMSR_JAX:000664, age 2-6 months old.<br>Mus musculus, strain: OMP-GCaMP3, age 2-6 months old
Wild animals	The study did not involve wild animals.
Reporting on sex	3 out of the 10 animals used for behavior were female. The animals used for calcium imaging and in vivo electrophysiology were all males.
Field-collected samples	The study did not involve samples collected from the field.
Ethics oversight	The study did not involve samples collected from the field.

Note that full information on the approval of the study protocol must also be provided in the manuscript.

Plants

Seed stocks	Not applicable.
Novel plant genotypes	Not applicable.
Authentication	Not applicable.
