# Supplementary material for: Perception and neural representation of intermittent odor stimuli in mice
Source: Nat Commun. 2026 Apr 29;17:5575. doi: 10.1038/s41467-026-72445-1 (PMC13304163; doi:10.1038/s41467-026-72445-1)
Supplement: Supplementary file 3 — Transparent Peer Review file [file 41467_2026_72445_MOESM3_ESM.pdf]

nature portfolio

Peer Review File

Perception and neural representation of intermittent odor stimuli in mice

Corresponding Author: Professor Venkatesh Murthy

This file contains all reviewer reports in order by version, followed by all author rebuttals in order by version.

Version 0:

Reviewer comments:

Reviewer #1

(Remarks to the Author)
The manuscript Perception and neural representation of intermittent odor stimuli in mice by Bouero et al. was a pleasure to review.

Odors in nature can be turbulent and hence intermittent as function of odor source distance. Recent work suggests the olfactory system is quite able to detect and discriminate fine temporal structure, which may inform mice of odor locations. However, it remains entirely unclear where and how in the brain behavioral decisions are made based on these temporal properties.
The Murthy lab trained mice to discriminate odor stimuli with 1-16 odor pulses during 1.5-10sec evaluation period, with left vs right lick responses. Mice are suggested to perform 3-4sec temporal integration, however no processing of accumulation of pulse number information, by increasing accuracy of estimated # pulses over time, was evident in the OB OSNs (optically imaged via GCaMP3) nor aPCx (tetrodes). Both behavior and neural responses showed dependence on breathing cycle, though about half aPCx neurons did not. Integrated over total assessment time about 20 aPCx neurons provide >80% accuracy. The discussion highlights several key issues and questions in a very interesting and thorough manner. Their DUNL method is very nice.
In my view this is a very interesting, timely and thorough paper, that required a lot of effort, and that was meticulously crafted and I hence have only a few comments.

General issues/questions
1. Given that the research question pertains to understanding how mice discriminate (these) intermittent odor signals, I found the discussion a bit vague on the explicit issue of what properties of this signal the mice may have used, despite a thorough discussion of error distributions pertaining to what the underlying processing model may be. Are they counting, integrating, using intermittency (fraction of time over threshold), etc, or some combination. I may have missed it but I only really see it in p17 L545-551 and p16L504. Indeed, SFig 1D and E are quite interesting in that the psychrometric functions seem duration and hence intermittency-range independent, while showing keen ability discriminating 6% differences in pulse counts/intermittency, etc. This suggests that counting discrete number of pulses rather than evaluating their intermittency is what these mice may have done. Intermittency is even more unlikely as the pulse time above say 10% threshold is about 100ms, leading to intermittency of 1 at 13-16 pulses at 1.25sec evaluation time, while the psychometric curve does not show such a ceiling.

2. The authors propose their data suggests a subitizing model. It would be nice to provide some back ground information about such a model, especially as it pertains to non-instantaneous stimuli as presented here.

3. P18 L606-608. About half aPCx neurons were interestingly found to respond in breath cycle invariant way. Besides perhaps suggesting OSN-MTC phase processing, could this imply temporal integration which may aid this task? Their accuracy seems not higher than other clusters, nor to increase at the 1 sec binning. The final paragraph of the discussion is intriguing.

4. It would be nice to demonstrate the airflow and pressure constancy (L701-702).

5. Fig 4F&G and L333-335: It appears spike based accuracy is very close to actual pulse accuracy, even merging in F4G.

Perhaps also show cumulative pulse based accuracy to highlight lack of spike based accumulated information?

6. Fig 4B right panel: colors are flipped for inhibitory vs excitatory groups?

7. Fig 5B: colors are flipped wrt legend?

5. Sfig3 define what phase 0 means - start inhalation? Are high dF/F correct?

Edits:
L206 subtracted from
L212 Np italicize
L810 error
L822 briefly explain erf
L847 briefly explain AIC

Reviewer #2

(Remarks to the Author)
The study introduces an innovative experimental approach to understand how olfactory input is processed and integrated when stimuli fluctuate. The authors designed a paradigm in which trained mice perform a forced-choice task: they must categorize sequences of odor pulses—delivered randomly over a 5-second window—as either “high” or “low” based on a predefined threshold. The precise control over both the number and timing of the pulses, along with their synchronization with the animals’ respiratory cycle, allows the researchers to explore how inhalation versus exhalation critically influences sensory perception.
In addition, the study combines behavioral data, advanced statistical analyses (such as logistic regression), and neurophysiological measurements—including recordings from the piriform cortex and imaging of olfactory sensory neurons—to offer a comprehensive view of olfactory processing. The findings show that although the mice can effectively discriminate these stimuli, and respiratory modulation significantly impacts perception, the cortical activity merely reflects the sensory input without showing signs of evidence accumulation. This observation suggests that other brain regions might be responsible for integrating evidence during decision-making. Notably, the mice achieve accuracies above 90% when the pulse count is clearly distant from the decision threshold (defined as 8.5 pulses), but accuracy drops significantly when the stimulus nears this threshold. Furthermore, logistic regression analyses indicate that pulses coinciding with inhalation are given more weight than those occurring during exhalation—a result confirmed by both behavioral and neural data, which directly links respiration to decision-making based on sensory input.
The manuscript is important because it addresses key questions about how fundamental physiological processes, such as respiration, affect sensory perception, placing it in the context of research on other sensory systems more broadly. However, there are critical issues that need to be addressed. For example, the absence of a cumulative signal in the piriform cortex, which is interpreted as a lack of integration of evidence, is controversial and deserves careful examination. This absence might stem from limitations in neuronal sampling or from analytical methods that are not sensitive enough to capture subtle changes over time. It is possible that integration signals present in specific neuron subgroups are being overlooked, or that evidence accumulation occurs in other, unrecorded brain areas. Furthermore, the decision model assumes that mice form an internal estimate of the number of pulses following a Gaussian distribution with noise that scales with the number of stimuli, implying a uniform, linear integration of information. Certainly, mice might use different strategies—for example, weighting early or late pulses more heavily, or integrating the pulses nonlinearly—which could affect both the final decision and the behavioral variability. The authors are encouraged to expand their discussion on these factors, providing additional analyses or experimental controls to rule out that the lack of accumulation in the piriform cortex is solely due to technical or methodological limitations.
Another concern involves the correction of the sensory signal using an adjustment based on respiratory synchronization to compute an “effective count” of pulses. Although the methods section describes this procedure, does not supply sufficient detail to ensure reproducibility and robustness. The authors should clearly explain how the adjustment parameters are determined —for example, what criteria were used to link the respiratory phase with the effectiveness of each pulse, and how the bin boundaries for the logistic regression were set. Additionally, they should specify the calibration and validation methods used to assess the variability of the “effective count” in different assays or subjects and include a sensitivity analysis to identify any possible limitations of the method. Details regarding the specific parameters used in the deconvolution via the DUNL method would also benefit from a clearer presentation. Including these extra details will enhance transparency and help other researchers replicate the study.
There are also minor issues that, while not detracting from the overall strength of the study, could improve the clarity and presentation of the manuscript. For example, some typographical errors and minor editorial inconsistencies need to be corrected, and some sections could also benefit from a more organized layout. The inclusion of schematics or flow diagrams in the description of the analytical methods and in the presentation of the results would greatly facilitate the understanding of both the experimental procedures and the statistical analyses.
In summary, the findings of the manuscript are novel and highly relevant and have the potential to significantly influence the field of olfactory neuroscience. However, the current version has several aspects that require clarification and refining to guarantee that its conclusions are solid and reproducible. Given the potentially transformative nature of this work, I recommend a major revision to address these critical points, thereby strengthening the study’s foundation and ensuring that its contributions are fully understood and replicable by the scientific community.

Reviewer #3

(Remarks to the Author)
The manuscript by Boero and colleagues uses a wide array of experimental and theoretical approaches to investigate how the olfactory system in mice transforms intermittent odor stimuli into neuronal codes along the olfactory system and how it correlates with behavior. In particular, they address how the olfactory system responds to repeated stimuli during longer time windows, how this depends on the sampling, or breathing, cycle and how olfactory sensory neurons and neurons in the anterior piriform cortex deferentially represent such stimuli. Their approach is sophisticated, their results interesting, novel and informative and deepen our understanding of sensory coding within the olfactory system and also in comparison the other sensory modalities.

Mayor comments
1 One issue the authors, somewhat briefly only mention in the discussion, is the question of the breathing frequency range their mice operate in. Guesstimating from the few sniff traces in their figures, the mice sniff around 4 Hz, which is only around 1/3 of their maximal, often used when exploring an olfactory environment, sniff frequency. They attribute this to the olfactory stimuli not being novel, but their head fixation also contributes to the lowered breathing rate. While this is unavoidable, the authors should discuss the implications. In particular, how their “long” inhalation phase (in comparison to a 10-12 Hz sniff rate) will affect not “missing” their odor stimuli, basically if they are over-counting the stimuli mice are perceiving. Alternatively, the mice might also learn (more on this issue below) to match their breathing rate to the task to improve their performance? The authors should state the breathing frequencies and limit their conclusion to that range.
2 Fig. 5G: So do the authors believe that APCx acts as a low pass filter? Given that cluster 3 is the largest?
3 It might be worth considering adding Suppl Fig. 5 to the main Fig. 5 given its relevancy.
4 How does the training to learn the task affect the encoding in the APCx? Are the observed 4 clusters a consequence of learning? Have the authors recorded from naïve mice using the same stimulus protocol?

Minor comments:
1 Line 77: Delete one “how”
2 Fig. 1E legend. Some of this text there (especially the conclusion) might be best moved to the main text?

Reviewer #4

(Remarks to the Author)
What are the noteworthy results?

Mice can be trained to discriminate odor pulses delivered during multi-second trials based solely on their number. This task required the subjects to integrate information over several seconds. Pulses that arrived during inspiration were more important for the decision process than those arriving during exhalation. The authors show, using calcium imaging, that pulse number can be represent at the
first synapse of the olfactory system in the olfactory bulbs. Finally, the authors show, using extracellular recordings, that the piriform or olfactory cortex is unlikely to be the integrator of odor pulse information given the stochastic and transient nature of its responses to a grouping of odor pulses.

Will the work be of significance to the field and related fields? How does it compare to the established literature? If the work is not original, please provide relevant references.

This work will be of interest to any investigator trying to understand how animals follow odor trails/plumes including the memorial processes involved. The work is derivative of earlier work by Gumaste et al., 2024 cited it the paper. But, to be fair, the lab head was also an early contributor to this line of investigation (e.g. Rokni et al., 2014).

Does the work support the conclusions and claims, or is additional
evidence needed?

Generally yes but see consideration below.

Are there any flaws in the data analysis, interpretation and conclusions? Do these prohibit publication or require revision?

Revisions may be necessary based on considerations below.

Is the methodology sound? Does the work meet the expected standards in your field?

Yes

Is there enough detail provided in the methods for the work to be reproduced?

Yes

Additional Considerations (in no particular order).

1. The use of a single odorant at an exceedingly high concentration---one unlikely ever to be found in nature---requires some justification. For example, such a high concentration would recruit low specificity glomeruli obscuring the bulbs “normal” response profile.

2. The use of identical pulse sizes also would seem highly artificial and thus deserves comment.

3. The authors make a lot out of the fact that pulses arriving during inspiration were more important for the decision process than those arriving during exhalation. But this might have a trivial explanation in that expired air does not travel the same path across the entirety of the mucosal sheet as inspired air. You would need to uncouple breathing from sniffing to get around this confound.

4. It’s unclear whether or not the authors tested mice without odor in the operant chamber. Showing that the mice work at chance levels without odor is the only way to be sure they are not using other cues besides odor, e.g. valve noise or vibrations or air movement changes, to discriminate S+ and S- trials (see Burt Slotnick’s work for example).

5. At least one example of the raw data from a mouse working to criterion in the operant task should be included if only in supplementary documents.

6. Some further interpretation of the data in Figure 1E seems warranted. For example, shouldn’t we have expected the rules of primacy and recency to apply; not at all what you are seeing.

7. Given that Gumaste et al., 2024 found sniffing to influence odor intermittency discrimination some justification for not measuring sniffing modulation in this work is appropriate. In any event, fast sniffing must have influenced the measurement of synchrony of inhalation/exhalation with pulse. Some further explanation about sniffing’s effect on synchrony measurements is warranted.

8. To be convincing, Supplementary Figure 1 should show a PIP sensor readout for multiple puffs including high frequency puffs.

9. Justification for use of ethyl valerate as the lone odorant used in the study should be added.

David M. Coppola, Ph.D.
Department of Biology
Randolph-Macon College

Version 1:

Reviewer comments:

Reviewer #2

(Remarks to the Author)
After evaluating the authors’ responses and the revised manuscript, I am satisfied that the main concerns raised in the previous review round have been fully addressed.
The authors now provide a clear and well-supported rationale for the absence of evidence-accumulation signals in the piriform cortex, placing their findings appropriately within the broader literature on sensory decision-making. Their response convincingly explains why the lack of ramping activity is unlikely to result from methodological limitations and clarifies that their conclusions apply specifically to the present task.
The expanded discussion on alternative decision strategies, as well as the justification of the modeling assumptions, also strengthens the conceptual framework of the study. Importantly, the authors have substantially improved methodological transparency by providing detailed explanations of the effective pulse calculation and the DUNL deconvolution procedure, which now meet standards for reproducibility.

Minor issues of clarity and presentation have been corrected, and the inclusion of additional schematics helps visualize key analytical steps.
Overall, the manuscript presents novel and rigorous work that will be of significance to the field. The revisions adequately support the main conclusions.
I recommend the manuscript for publication

Reviewer #3

(Remarks to the Author)
The manuscript by Boero and colleagues uses a wide array of experimental and theoretical approaches to investigate how the olfactory system in mice transforms intermittent odor stimuli into neuronal codes along the olfactory system and how it correlates with behavior. In particular, they address how the olfactory system responds to repeated stimuli during longer time windows, how this depends on the sampling, or breathing, cycle and how olfactory sensory neurons and neurons in the anterior piriform cortex differentially represent such stimuli. Their approach is sophisticated, their results interesting, novel and informative and deepen our understanding of sensory coding within the olfactory system and also in comparison the other sensory modalities.

In their revised manuscript, the authors added new analysis to address questions raised and also discussed them appropriately and expertly in the discussion. I would like to thank them for the added revisions to improve their manuscript. I have no further concerns.

Reviewer #4

(Remarks to the Author)
All of my comments on the original draft have been thoroughly dealt with in the resubmission.

Open Access This Peer Review File is licensed under a Creative Commons Attribution 4.0 International License, which permits use, sharing, adaptation, distribution and reproduction in any medium or format, as long as you give appropriate credit to the original author(s) and the source, provide a link to the Creative Commons license, and indicate if changes were made.
In cases where reviewers are anonymous, credit should be given to 'Anonymous Referee' and the source.
The images or other third party material in this Peer Review File are included in the article's Creative Commons license, unless indicated otherwise in a credit line to the material. If material is not included in the article's Creative Commons license and your intended use is not permitted by statutory regulation or exceeds the permitted use, you will need to obtain permission directly from the copyright holder.
To view a copy of this license, visit https://creativecommons.org/licenses/by/4.0/

Response to Reviewers

(Reviewer comments are in **bold** and our responses in regular type)

We have exhaustively reworked the manuscript to fully address the reviewers' suggestions in order to improve it and provide further support for our conclusions. The manuscript has been changed accordingly and a point-by-point response to major and minor concerns raised by both reviewers is included below.

<u>Reviewer 1</u>

The manuscript Perception and neural representation of intermittent odor stimuli in mice by Bouero et al. was a pleasure to review.

Odors in nature can be turbulent and hence intermittent as function of odor source distance. Recent work suggests the olfactory system is quite able to detect and discriminate fine temporal structure, which may inform mice of odor locations. However, it remains entirely unclear where and how in the brain behavioral decisions are made based on these temporal properties.

The Murthy lab trained mice to discriminate odor stimuli with 1-16 odor pulses during 1.5-10sec evaluation period, with left vs right lick responses. Mice are suggested to perform 3-4sec temporal integration, however no processing of accumulation of pulse number information, by increasing accuracy of estimated # pulses over time, was evident in the OB OSNs (optically imaged via GCaMP3) nor aPCx (tetrodes). Both behavior and neural responses showed dependence on breathing cycle, though about half aPCx neurons did not. Integrated over total assessment time about 20 aPCx neurons provide >80% accuracy. The discussion highlights several key issues and questions in a very interesting and thorough manner. Their DUNL method is very nice.

In my view this is a very interesting, timely and thorough paper, that required a lot of effort, and that was meticulously crafted and I hence have only a few comments.

We thank the reviewer for their kind support of this work and for the helpful comments, which we answer in detail below.

<u>General issues/questions</u>

1. Given that the research question pertains to understanding how mice discriminate (these) intermittent odor signals, I found the discussion a bit vague

on the explicit issue of what properties of this signal the mice may have used, despite a thorough discussion of error distributions pertaining to what the underlying processing model may be. Are they counting, integrating, using intermittency (fraction of time over threshold), etc, or some combination. I may have missed it but I only really see it in p17 L545-551 and p16L504. Indeed, SFig 1D and E are quite interesting in that the psychrometric functions seem duration and hence intermittency-range independent, while showing keen ability discriminating 6% differences in pulse counts/intermittency, etc. This suggests that counting discrete number of pulses rather than evaluating their intermittency is what these mice may have done. Intermittency is even more unlikely as the pulse time above say 10% threshold is about 100ms, leading to intermittency of 1 at 13-16 pulses at 1.25sec evaluation time, while the psychometric curve does not show such a ceiling.

We appreciate the reviewer's feedback on this specific point. As it has been stated in the manuscript and by the reviewer, one possibility is that mice are counting the number of odor pulses for discriminating between 'High' or 'Low' trials. First, our PID recordings (Supplementary Figure 1A) confirm that odor pulses are presented as a brief, transient sensory input. Therefore, our olfactometer is capable of delivering sensory stimuli with temporal properties good enough to be discrete, a key requirement for any counting-based algorithm. Secondly, our behavioral results in Supplementary Figure 1C (previously Supplementary Figure 1D) show clearly how mice can still have a good performance in the categorization of the total number of odor pulses with different sampling window durations. Since the range of pulses delivered was maintained for the different trial lengths, increases in sampling window duration will necessarily reduce the range of intermittencies experienced by the animal (and vice versa), as shown in Supplementary Figure 1D (previously Supplementary Figure 1E). For instance, when a 5s sampling window was used, the range of intermittency values was between 0 and 0.15. Also, an additional odor pulse produced a little increase in intermittency, rendering total pulse counts of 6 and 11 (both equally distant of the decision boundary) with just a difference of ~0.05 in intermittency but performance levels of ~70%. Previous work exploring odor intermittency discrimination (Gumaste et al., 2024), showed that mice can discriminate odor stimuli with low intermittencies (<0.15) from those with high intermittencies (ranging from 0.2 to 0.8). Therefore, we cannot entirely disregard the possibility that mice were performing the task by identifying these small differences in intermittency, but (1) the fact that the differences we observed are smaller than what was tested in the literature, and (2) the fact that the same mice can perform the task in different ranges of intermittency, offer strong evidence against intermittency being the sole olfactory statistic guiding mice decisions in the task

Taking all that into account, the Discussion now reads (lines 548-579 – new text underlined here):

Mice in our task were asked to distinguish and categorize random sequences of brief odor pulses of the same odorant based on whether the integrated signal was below or above a threshold. Importantly, PID recordings confirmed that, when pulses happened close in time, odor signals could sum and lead to short intervals of higher intermittency and overall amplitude. Therefore, by building stochastic sequences of odor pulse delivery, our olfactometer could successfully recreate naturalistic odor profiles. The signal integrated by mice for decision making could be related to one or a combination of statistical features that characterize odor plumes: intermittency, whiff (or blank) count and frequency and concentration profile (Celani et al., 2014)*. In the current task, the duration of each pulse and the duration of the sampling window were fixed, allowing us to control the values of those statistics by varying the total number of odor pulses delivered. For instance, by increasing or decreasing the size of the sampling window but keeping the total pulse counts the same, we could manipulate the range of intermittencies of the olfactory stimuli presented (Supplementary Fig. 1D). Mice were not only capable of categorizing odor stimuli across sessions with different ranges of intermittencies (Supplementary Fig. 1C), but more remarkably, they were able to accurately discriminate pulse counts at opposite sides of the decision boundary that only differed in ~0.05 units of intermittency, a value significantly lower than the one shown in a recent report* (Gumaste et al., 2024)*. Although we cannot rule out the possibility that mice are capable of extremely fine intermittency discrimination, the fact that they performed correctly across different intermittency ranges but same pulse counts supports the idea that they were integrating odor pulse counts rather than intermittency. It is also important to note that which statistic is being integrated can also be a result of the experimental design: the sensory stimuli in our task was composed of a stochastic array of odor pulses with temporal properties that supported their discretization, whereas in Gumaste (2024) animals were trained in odor profiles that varied in intermittency but with a lower range of variation in the number of whiffs. Therefore, our observation of odor pulse (whiff) count discrimination independent of intermittencies is not necessarily in conflict with their observation of intermittency discrimination irrespective of whiff counts. The question regarding which feature, or statistic of olfactory stimuli animals integrate during odor-guided navigation is still open, but our results indicate that mice are capable of integrating whiff counts for decision-making. Assays acutely manipulating the duration of the sampling window during catch trials have been used as a strategy to disambiguate the variable that animals integrated* (Raposo et al., 2012)*, and they can be incorporated in future experiments.*

Modelling results are insufficient to confirm what statistical feature animals were accumulating. Therefore, we made very subtle changes to that part of the Discussion (lines 640-645):

As noted above, although models for counting discrete stimuli seem to be a valid approach to approximate the accumulation of olfactory statistics in our task, modelling results might not be sufficient to prove that mice are implementing a counting strategy over discretized statistics - such as odor pulse/whiff counts - as opposed to just accumulating temporal variations of a continuous variable such as odor intermittency or pulse rate.

2. The authors propose their data suggests a subitizing model. It would be nice to provide some back ground information about such a model, especially as it pertains to non-instantaneous stimuli as presented here.

We appreciate the Reviewer's concern about skipping the details of the concept of Subitizing that we were using in the Discussion of the manuscript. We added a sentence with a rephrasing of Von Glasersfeld's reinterpretation of the original definition made by Kaufman in 1949. Since we do not exactly know the perceptual algorithms supporting the decisions in our task, we do not intend to imply that mice are using subitizing for choosing the 'low' side when just a few pulses are presented. Our intention is to connect our analysis of decision noise and our model to hypotheses from the field of numerosity estimation used explain these types of perceptual observations in human and other animals.

The paragraph now reads (lines 615-628):

"*Sensory evidence accumulation is not only defined by the memory displayed by the accumulator, but also by the level of noise in the accumulating signal. Applying a similar approach to a previous report* (Scott et al., 2015)*, we showed that noise in the estimation of pulses was initially constant for low pulse counts until a point after which it started to scale with the number of pulses (Figure 2G). Constant noise in the estimation of low quantities has been shown before in multiple animal and human experiments, and has been explained by the concept of 'subitizing'* (Davis & Pérusse, 1988; Gallistel & Gelman, 2000)*. Subitizing refers to the perceptual process allowing animals to immediately provide a correct estimation (noiseless) of the total number of perceptual items when the number of elements is small (usually below 5)* (Kaufman et al., 1949; von Glasersfeld, 1982)*. However, our noise modelling results challenge subitizing in two main aspects: (i) the estimation of low quantities is not noiseless and (ii) the constant noise extends further than four to five stimuli* (Gallistel & Gelman, 2000)*.*

P18 L606-608. About half aPCx neurons were interestingly found to respond in breath cycle invariant way. Besides perhaps suggesting OSN-MTC phase processing, could this imply temporal integration which may aid this task? Their accuracy seems not higher than other clusters, nor to increase at the 1 sec binning. The final paragraph of the discussion is intriguing.

We appreciate the Reviewer's question about the functional role of this cluster. We believe that if the cluster was involved in some temporal integration, their decoding accuracy for the task should be higher than the accuracy levels reached by the other clusters. But that is not the case, as depicted in new Figure 5J-L (old Supplementary Figure 5), which shows that decoding accuracies are very similar between clusters, with spikes from the phase-invariant neurons being slightly worse at predicting task-relevant variables. We are not entirely clear why the reviewer suggests that he breath-cycle invariant response should imply temporal integration – these responses are still transient and time-locked to the odor pulse onset, but just happen to have approximately the same amplitude independent of which breathing phase the pulse falls in. Therefore, it has more to do with how the odor pulse timing with respect to the breathing phase affects aPCx response. This is illustrated in the cartoon below where we sketch how the response of a typical neuron in cluster 1 or 3 (Figure 5H) look like to pulses falling on 2 different breathing phases.

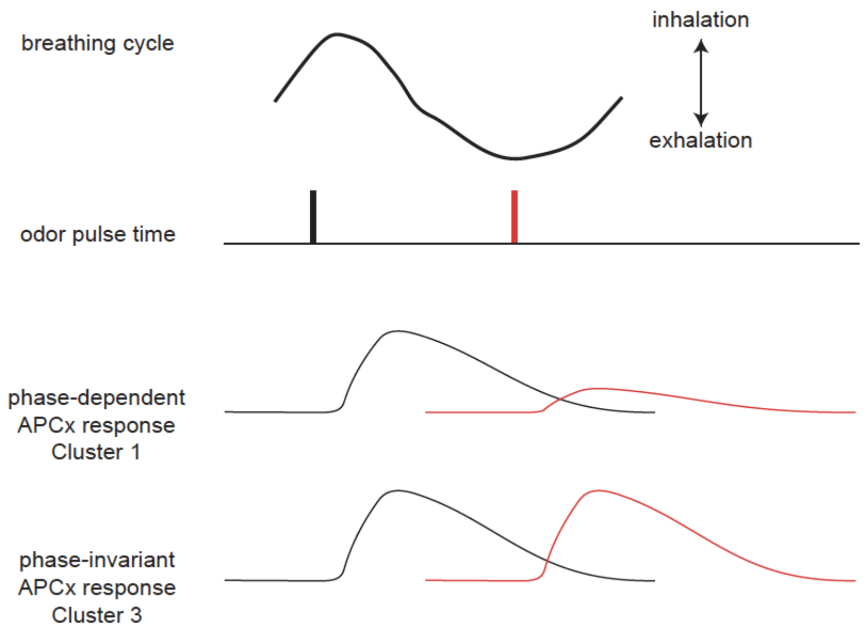

Based on the point raised by the Reviewer, we have added a brief comment in the Discussion (lines 729-738):

Another source of variability is the differential tuning of APCx neuron response to the timing of odor pulses within the breathing cycle. We found that roughly 50% of the APCx

population responded equally to odor pulses falling at any time during the breathing cycle, whereas the remaining 50% may be tuned to different pulse timings over the breathing cycle. <u>Remarkably, clusters showed similar levels of decoding accuracy irrespective of their phase-tuning (Figure 5J-L), arguing against a preferential involvement of a specific cluster(s) in the temporal integration of odor inputs.</u> It is important to note that neuronal response amplitudes were estimated by the DUNL method by grouping spikes in 50 ms bins, which may influence inferences about the sparseness and phase-tuning of the responses.

The final paragraph of the discussion is aimed at proposing a roadmap of other brain areas that could be accumulating olfactory evidence and driving the binary decisions, given we did not find signatures of that in the APCx. We highlighted how APCx is connected with multiple areas in the prefrontal cortex, and how olfactory inputs can reach the striatum through the olfactory tubercle. Both stratium and prefrontal cortex -as well as areas heavily connected to prefrontal cortex, such as the Posterior Parietal Cortex- have been implicated in the accumulation of sensory evidence for tasks using visual, auditory or tactile stimuli in rodents and primates. Therefore, it would not be surprising that any of those areas could be also involved in the accumulation of olfactory evidence. We aim to pursue this actively in the future, but since we do not have additional experimental data, we prefer to keep the discussion in the paper brief.

4. It would be nice to demonstrate the airflow and pressure constancy (L701-702).

We appreciate the concern of the Reviewer about this specific point. We used our Honeywell flow sensor to measure the flow disturbances produced by valve switching during odor pulse delivery. We found that odor pulse delivery led to flow transients in the order of 25 standard cubic centimeters per minute (sccm) (peak-to-peak) that lasted approximately 70 ms (see Rev Figure 1 below).

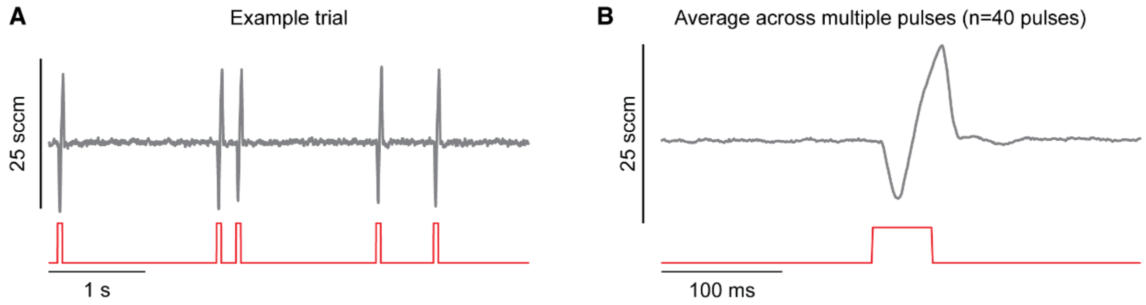

Rev Figure 1. Fluctuations in airflow during odor pulse delivery. **(A)** Example trial with 5 clean air 50 ms pulses (red trace) presented over a 5s window and the measurements from the airflow sensor (gray trace). **(B)** Average flow transient elicited by a clean air pulse. Same color scheme as in **(A)**.

Although these fluctuations were smaller and briefer in time than what has been reported in the literature to be discriminable by mice (Mahajan et al., 2024), this finding triggered the concern that mice could have integrated airflow fluctuations rather than the odor pulses to perform the task. A related concern was raised by Reviewer 4, who pointed out that mice could be attending to other cues during the task and suggested a control experiment in which odorless pulses were presented. Therefore, we decided to train a new cohort of animals (n=3) in the task and once they were proficient in the task, we introduced catch trials (10% of total trials in a session) in which pulses were delivered from a valve connected to clean air. Pulse timing and counts in the catch trials were generated with the same parameters as for the regular odor pulses, and reward sites followed the same dependency based on total pulse counts. Performance in catch trials is currently shown in Supplementary Figure 1E and shows clearly that mice largely categorized clean air pulses as 'Low Trials', irrespective of the total air pulse count. The same animals still showed good performance in odor pulse categorization. We believe this result proves that animals were integrating the odor information for making their decisions rather than integrating other cues, such as sounds linked to valve opening or transients in airflow.

We added a comment about this experiment in Results (lines 163-169)

To test if mice were using pressure or airflow changes as cues for decision making, we introduced catch trials with varying number of clean air pulses (instead of the odorized air), which were rewarded by following the same rule as for the odor pulses. Catch trials were categorized by mice as 'Low' trials irrespective of the total pulse count (Supplementary Fig. 1E, grey traces), confirming that they were basing their decision on the integrated number of odor inputs rather than mechanosensory or auditory signals arising from any airflow transients or valve openings.

And we also add a clarification about this in the Methods section (lines 829-850):

Odor pulse delivery was accomplished using a custom olfactometer equipped with fast proportional solenoid valves (EV-P-05-09-A0; Clippard) controlled from a Teensy 3.2 (PJRC). The fast kinetics of the solenoid valves used allowed us to switch between constant clean air delivery and 50 ms activations of another solenoid valve connected to a vial containing a diluted monomolecular odorant. 5% (v/v) ethyl valerate (Sigma-Aldrich) was used for all the experiments, except Supplementary Fig. 1E that shows the psychometric curves for a separate cohort trained with 5% (v/v) methyl butyrate (Sigma-Aldrich). The resulting odor stream was sent directly to a 3D printed mask fitted to the snout of the animal. For behavior and electrophysiology experiments, ethyl valerate (or methyl butyrate) dilutions were done in mineral oil (Sigma-Aldrich), whereas for imaging experiments they were done using di-ethyl-phthalate as solvent (Sigma-Aldrich). Catch trials were introduced by delivering 50 ms odor pulses from a separate valve connected

to a clean air vial. The mask was also connected to an airflow sensor (AWM3100V; Honeywell) to monitor animal breathing during the task, as well as to a vacuum line to ensure quick removal of the odorized air from the snout of the mouse. Generally, a constant airflow of 1L/min was kept during the experiment, but we identified small transients of 25 sccm during odor pulse delivery. Vacuum flow from the mask, as well as the airflow input to the olfactometer, were regulated using two different mass-flow controllers (QPV1, Proportion Air). Photoionization detector (PID; Aurora Scientific) measurements were performed in absence of any animals but in the same conditions of an experimental session, with the probe of the PID placed at the approximate position of the nostrils of the mouse.

And also in lines 865-866:

Catch trials were presented in a separate experiment with a cohort of 3 animals and represented 10% of the total trials in a session.

5. Fig 4F&G and L333-335: It appears spike based accuracy is very close to actual pulse accuracy, even merging in F4G. Perhaps also show cumulative pulse based accuracy to highlight lack of spike based accumulated information?

We appreciate the Reviewer's suggestion, and we have included now the decoding of task relevant variables using cumulative intervals for the odor pulses and spikes in Supplementary Figure 5A-C. Decoding accuracy for 'Trial Identity' (Supplementary Figure 5B) - a variable directly determined by the total number of pulses - reaches approximately 1 (maximum value) when the total pulse count over 5 seconds is used, whereas the accuracy based on neuronal spikes in the APCx population is around 0.8. Nonetheless, it is important to note that good levels of decoding accuracy with cumulative intervals can be achieved even when a neuron spikes upon sensory input presentation without any accumulated response driving the decision. If neurons in APCx were acting as an evidence accumulation hub, firing rates would be ramping towards the end of the sampling period for trials with pulse counts easily indicating the choice (1 and 16, for example), which would lead to a significant increase in the decoding accuracy for the last independent intervals. Our data, summarized in Figures 4F-G and Supplementary Figures 5B-C, do not show any signs of that, arguing against APCx neurons as evidence accumulators.

Below is an illustration of the logic. On the left, the firing rates over 5 seconds are shown for a putative neuron with transient pulse response for 4 trials of low pulse rate (top) and high pulse rate (bottom). Note that at the end of this 5 second period, the average firing rates are not different – hence decoding of high-vs-low will be poor. In the right column, we illustrate what an evidence-accumulating response might look like. Here, at the end of the stimulus period, the instantaneous firing rate is clearly higher for the high pulse

rate. While this type of neuron was not detected in APCx, we anticipate their existence in other brain areas mediating choice.

We added a brief comment on the Results section about this point (lines 379-381):

Decoding based on cumulative 500 ms intervals revealed a higher accuracy when spikes over the whole sampling period were considered, but in agreement with the previous results, no abrupt increases in decodability were seen for later intervals (Supplementary

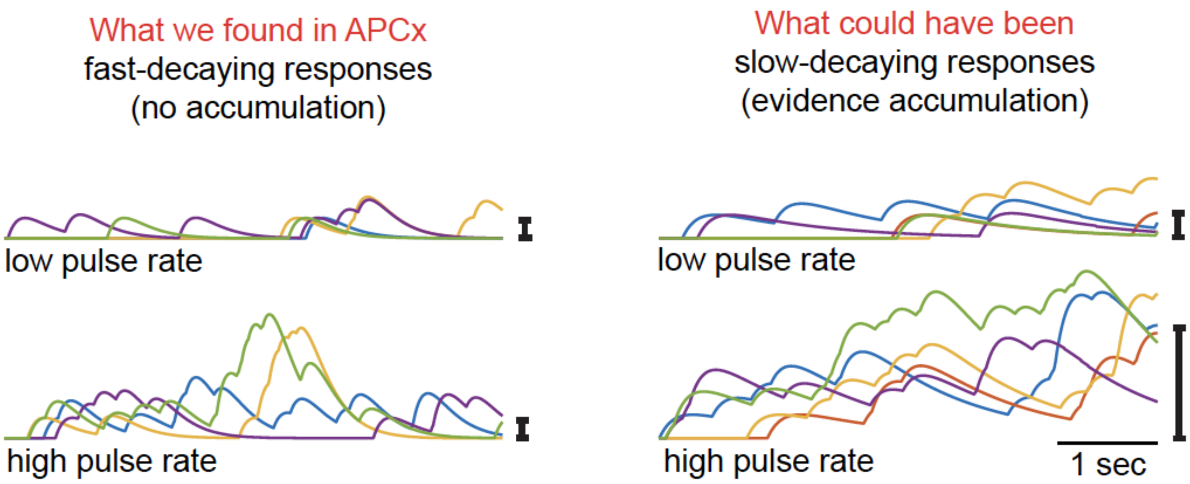

Fig. 5A-C).

Caption of Supplementary Figure 5 now reads (lines 81-87 in Supplementary Information file):

Supplementary Figure 5. ***Heterogeneity in the phase-tuning and estimated response kernels of APCx neurons.*** ***(A)*** *Average multiple linear regression R^2 using population spike counts over cumulative 500 ms intervals to predict total pulse counts.* ***(B-C)*** *Teal traces depict prediction accuracy of logistic regression on trial identity* ***(B)*** *and behavioral choice* ***(C)*** *using population spike counts over cumulative 500 ms intervals. Gray traces are the accuracies obtained when target labels were permuted. Red traces show the decoding based on the odor pulse information available, whereas pink traces correspond to the accuracies after permutation of samples.* ***(D) (…)***

6. Fig 4B right panel: colors are flipped for inhibitory vs excitatory groups?

We thank the Reviewer for pointing out this mistake. We now have changed the colors in the right panel of Figure 4B to be coherent with the left panel and the pie chart: green trace reflects the average spike counts per second vs total odor pulse counts for those

neurons that increase their firing with the total number of odor pulses presented, whereas the orange trace corresponds to the neurons that decrease their firing rate with increases in odor pulse counts.

7. Fig 5B: colors are flipped wrt legend?

We thank the Reviewer for pointing out this mistake. Colors were changed in the caption of Figure 5, and lines 484-490 now read:

Figure 5. APCx population responses to odor pulses are stochastic and heterogeneous. *Example of DUNL the implementation for the neuron marked with the blue arrowhead in the raster plot shown in (**A**) for a specific trial. (**B**) Timing of spikes and odor pulses are used to train a model that learns a specific event-based kernel for each neuron ('kernel', green trace), as well as which sensory stimuli were detected and the intensity of the neuronal response (blue trace, also referred as 'response amplitude'). Convolution of response amplitudes with the kernel provides an estimation of the firing rate of the neuron ('firing rate estimation', gray trace).*

5. Sfig3 define what phase 0 means - start inhalation? Are high dF/F correct?

For Supplementary Figure 3 we defined respiratory phase in the same way as in Figure 2: 0 and 2π correspond to the peaks of consecutive exhalations. Therefore, 0 indicates the time point at which the inhalation part of the cycle starts.

We thank the Reviewer for asking about the dF/F values in Supplementary Figure 3, because it made us realize that there was an issue with the y-axes in those plots. Specifically, the peak values for the mean glomerular response in the representative traces shown in Figure 3B are ~ 0.1 dF/F, whereas the peak responses for the mean glomerular response in the plots in Supplementary Figure 3A-C (Now Supplementary Figure 4A-C) are 0.4-0.6 dF/F. The source of the discrepancy is that Figure 3B shows the raw glomerular responses (and their mean across glomeruli), whereas the values in Supplementary Figure 4A-C were normalized to the 99.5th percentile of the calcium signal across the entire behavioral session.

Therefore, we made a series of changes to amend this important omission:

1) We added the word 'Normalized' to the Y-axes of Supplementary Figure 4A-C.
2) Caption in Supplementary Figure 4 (former Supplementary Figure 3) now reads (lines 70-77 in Supplementary Information):

Supplementary Figure 4. Phase-dependence of individual glomerular responses. *(**A-C**) GCaMP3 fluorescence (<u>ΔF/F normalized to the 99.5th percentile</u>) as a function of odor pulse timing relative to the respiratory phase for the three different animals tested. Each thin trace corresponds to a single glomerular ROI (all ROIs that showed significant GCaMP3 response), whereas the thick green line is the average across ROIs. The dependence on pulse timing for response amplitude can be verified even at the level of single glomerulus. (**D**) GCaMP3 ΔF/F <u>(normalized to the maximum value)</u> as a function of odor pulse timing relative to the respiratory phase <u>averaging</u> across all ROIs in each animal. Data in this panel is expressed as mean $\pm$ CI 95% (n = 3 animals).*

3) We also clarified this in the Methods section (lines 1061-1072):

<u>Analysis of imaging data:</u> Images were processed using both custom and available MATLAB (MathWorks) scripts. Motion artifact compensation and denoising were done using NoRMCorre (Pnevmatikakis & Giovannucci, 2017)*. The CaImaN CNMF pipeline* (Pnevmatikakis et al., 2016) *was used to select and demix ROIs. dF/F values were calculated using the mean of a baseline period of at least 20 frames. To identify glomeruli that were significantly odorant-modulated, three points centered on the peak dF/F signal after odorant delivery were averaged. Glomeruli were classified as significantly odorant-modulated if their peak averaged response exceeded 2.5 standard deviations of the baseline noise. <u>The dF/F signal for each odor-modulated glomerulus was normalized to the 99.5th percentile across the entire session. Estimation of the dependence of OSN calcium signals with odor pulse arrival time within the respiratory cycle was achieved by first selecting odor pulses that were isolated by at least 1 s both before and after their occurrence and then extracting the calcium signals around those peaks.</u>*

L206 substracted from:

We thank the reviewer for pointing out this mistake. The size of the caption was significantly reduced to comply with the editorial requirement of 350 words or less. Now lines 241-243 read:

*'**Center:** ΔN is calculated by <u>subtracting</u> N_p <u>from</u> the estimate of the decision boundary, N_b, also following a Gaussian distribution with variance σ_b^2.'*

L212 Np italicize

We thank the reviewer for pointing out this mistake. This line was removed from the caption to comply with the editorial requirement of 350 words or less.

L810: Error

We thank the reviewer for pointing out this mistake. There was a duplicated text box that got on top of text in line 810. Now lines 950-954 read:

The second source of noise arises from the internal estimation of the N_b, a parameter that we also assumed follows a normal distribution with constant variance σ^2_b for all N_p. Then, as it is shown in Equation 2, we propose that licking decision on a trial is based on ΔN, i.e. the internal representation that the animal has of the difference between sensed pulses (N_{est}) and the decision boundary (N_b).

L822: briefly explain erf.

Erf in Equation 4 refers to the error function used for calculating the area under the distribution determining the probability of choosing the ‘High’ side.

Now lines 964-965 read:

Erf refers to the error function used to calculate the area under the normal distribution that reflects the probability of choosing ‘High’.

L847 briefly explain AIC

AIC refers to Akaike Information Criterion, a statistical estimator used for comparing and selecting models. It takes into account goodness-of-fit, while adding penalties with increasing number of variables in the model. In this case, we used it for selecting the neurons that actually showed changes in their activity (increase or decrease in firing) correlated with the total number of odor pulses presented in a trial. Briefly, we fitted a linear model with the spikes from the entire neuronal population (each neuron would be a variable in the linear model) and fitted multiple additional models by removing neurons one by one. Only those neurons that led to a reduction in AIC were kept for future analysis (except for the dependence of decoding with the size of neuronal population).

Now the paragraph reads (lines 1005-1016):

Analysis of APCx spiking data. For each behavioral session, spikes from the entire APCx neural population were used to fit a linear regression model aimed at predicting the total number of odor pulses delivered on a trial. Neurons were sorted based on the p-values obtained from the regression and new linear regression models were fitted excluding neurons one by one. Next, the different regression models (each containing spikes from different subsets of neurons) were compared using the Akaike Information Criterion (AIC). AIC is an objective statistical tool used for comparing and selecting the best-fitting model

from a set of candidate models. It takes into account goodness-of-fit but also add penalties for the number of parameters (individual neurons, in this case). Then, only those neurons whose spikes led to a reduction in the AIC of the regression model fitted for every session were considered for all subsequent analysis (except for the dependence on the decoding with the size of population in Figure 4 F-H).

Reviewer 2

The study introduces an innovative experimental approach to understand how olfactory input is processed and integrated when stimuli fluctuate. The authors designed a paradigm in which trained mice perform a forced-choice task: they must categorize sequences of odor pulses—delivered randomly over a 5-second window—as either "high" or "low" based on a predefined threshold. The precise control over both the number and timing of the pulses, along with their synchronization with the animals' respiratory cycle, allows the researchers to explore how inhalation versus exhalation critically influences sensory perception.

In addition, the study combines behavioral data, advanced statistical analyses (such as logistic regression), and neurophysiological measurements—including recordings from the piriform cortex and imaging of olfactory sensory neurons—to offer a comprehensive view of olfactory processing. The findings show that although the mice can effectively discriminate these stimuli, and respiratory modulation significantly impacts perception, the cortical activity merely reflects the sensory input without showing signs of evidence accumulation. This observation suggests that other brain regions might be responsible for integrating evidence during decision-making. Notably, the mice achieve accuracies above 90% when the pulse count is clearly distant from the decision threshold (defined as 8.5 pulses), but accuracy drops significantly when the stimulus nears this threshold. Furthermore, logistic regression analyses indicate that pulses coinciding with inhalation are given more weight than those occurring during exhalation—a result confirmed by both behavioral and neural data, which directly links respiration to decision-making based on sensory input.

The manuscript is important because it addresses key questions about how fundamental physiological processes, such as respiration, affect sensory perception, placing it in the context of research on other sensory systems more broadly.

We greatly appreciate the reviewer's positive summary of our work.

However, there are critical issues that need to be addressed.

For example, the absence of a cumulative signal in the piriform cortex, which is interpreted as a lack of integration of evidence, is controversial and deserves careful examination. This absence might stem from limitations in neuronal sampling or from analytical methods that are not sensitive enough to capture subtle changes over time. It is possible that integration signals present in specific neuron subgroups are being overlooked, or that evidence accumulation occurs in other, unrecorded brain areas. Furthermore, the decision model assumes that mice form an internal estimate of the number of pulses following a Gaussian distribution with noise that scales with the number of stimuli, implying a uniform, linear integration of information. Certainly, mice might use different strategies—for example, weighting early or late pulses more heavily, or integrating the pulses nonlinearly—which could affect both the final decision and the behavioral variability. The authors are encouraged to expand their discussion on these factors, providing additional analyses or experimental controls to rule out that the lack of accumulation in the piriform cortex is solely due to technical or methodological limitations.

We appreciate the reviewer's suggestion of taking other alternative explanations for our experimental results.

With respect to the lack of evidence integration in piriform, we would like to clarify that it is not our intention to claim that the piriform cortex cannot integrate olfactory evidence in general, especially since there is a great body of literature characterizing the piriform cortex as a sensory-associative cortex. In our task we found that APCx neurons clearly respond to odor pulses. However, we did not find changes over the sampling period that were indicative of temporal integration, especially if we compare them to what has been shown in other evidence accumulation centers in the brain during tasks that required the integration of visual or auditory information. A good example of this is available in Figure 1C from Hanks et al. (2015), where it is evident that neurons in the posterior parietal cortex (PPC) displayed a steady increase (ramp) in their firing rates proportional to the amount of sensory information pointing in their preferred direction. Remarkably, the temporal evolution of firing rates in our APCx recordings has shown how different neurons may respond by increasing or decreasing firing rates with more odor pulses but did not reveal any form of ramping indicative of integration for decision. We illustrate this logic in our response to point #5 of Reviewer #1. This was particularly coherent with our decoding analysis, that failed to find a difference in decoding accuracy of neurons across the sampling window. Since we were using similar experimental and analytical approaches than those used in the literature of rodent perceptual evidence accumulation, we believe that the lack of support for APCx having an evidence accumulation role in the current task is not an artifact of experimental or analytical limitations.

We hypothesize that while piriform cortex may not have a direct role in evidence accumulation, it is an important brain area for broadcasting key aspects of the sensory stimuli, such as the odor identity or the timing of odor information with respect to the breathing cycle. In this scenario, the current work undermines the possibility of odor cues being already temporally integrated in early sensory cortices and supports a model in which piriform outputs are transmitted to other brain areas for its integration, as it happens for other sensory modalities. For instance, previous reports using visual or auditory tasks revealed that neurons in areas such as the anterodorsal striatum and the posterior parietal cortex displayed changes in their individual firing - as well as in the population response - indicative of perceptual accumulation of evidence. It is probable that the same areas are mediating the accumulation of olfactory evidence in the current task, therefore, one of our future goals is to perform in vivo electrophysiological recordings from those areas during behavior.

Regarding the points about the decision modelling, we agree with the reviewer that our model assumes a linear integration of the information. We took this as a first approach to model the decision noise in the task, since it was shown before that could accurately capture a significant part of the noise during binary decisions that could not be explained with traditional drift-diffusion models (Scott et al., 2015). Nonetheless, our results in Figure 1E explicitly show how mice may give more weight to odor pulses coming around the middle of the sampling period, rather than those coming at the beginning or the end of it. Future models of the decision could, as the reviewer suggested, allow for non-linear integration of odor pulses -for instance, for pulses coming close in time - and also incorporate a time-dependent weight component to account for the results of our mixed model logistic regression analysis.

Another concern involves the correction of the sensory signal using an adjustment based on respiratory synchronization to compute an “effective count” of pulses. Although the methods section describes this procedure, does not supply sufficient detail to ensure reproducibility and robustness. The authors should clearly explain how the adjustment parameters are determined —for example, what criteria were used to link the respiratory phase with the effectiveness of each pulse, and how the bin boundaries for the logistic regression were set. Additionally, they should specify the calibration and validation methods used to assess the variability of the “effective count” in different assays or subjects and include a sensitivity analysis to identify any possible limitations of the method.

We appreciate the reviewer’s concern regarding the methods for effective pulse calculation, and we can clarify this further. First, we pooled data across animals from all trials used for generating the psychometric in Figure 1D (19,911 trials across 7 mice). For

each trial, the breathing signal was split into individual breaths, and each breath was divided into 15 bins. Therefore, the length of each bin could vary from one breath to the other depending on the length of each breath, but each bin will represent a specific phase of the breathing cycle across multiple bins. Next, each odor pulse was assigned to a specific phase bin in the breath based on the arrival time, and a phase histogram was computed for each trial compiling the number of pulses that arrived at each of the 15 phase bins. Subsequently, the binary logistic regression model was fitted for each trial following the equation below:

$$y \sim \sigma\left(\sum_{n=1}^{n=15} x_n\, w_n\right)$$

Where *y* is the binary choice (0 = 'low' side, 1='high' side) done by the mouse in that trial, x_n represents the number of pulses in bin *n*, and w_n corresponds to the weight associated with that specific bin. FIgure 2D depicts the values of the w_n for each of the phase bins.

For the effective pulse calculation, we computed dot product between the phase histogram (i.e. a vector indicating how many pulses occurred at each of the 15 phase bins) with the vector of weights plotted in FIgure 2D. Since odor pulses can be delivered at any time during the sampling window, trials with the same delivered pulse count can differ in the arrival time of those pulses at each breath. Then, it is logical that trials with the same number of pulses delivered display variability in the effective pulse count, as it is shown in Supplementary Figure 2D.

We added part of this in the Methods section (lines 987-1003):

Perceptual weight estimation through binary logistic regression: For each trial, the breathing signal was split into individual breaths, and each breath was divided into 15 bins. Therefore, the length of each bin could vary from one breath to the other depending on the length of each breath, but each bin represented a specific phase of the breathing cycle across multiple bins. Next, each odor pulse was assigned to a specific phase bin in the breath which arrived, and a phase histogram was computed for each trial compiling the number of pulses that arrived at each of the 15 phase bins. Subsequently, the binary logistic regression model was fitted for each trial following Equation 5 below:

$$y \sim \sigma\left(\sum_{n=1}^{n=15} x_n\, w_n\right) \qquad \text{Equation}$$

Where y is the binary choice (0 = 'low' side, 1='high' side) done by the mouse in that trial, x_n represents the number of pulses in bin n, w_n corresponds to the weight associated with that specific bin, and σ indicates the logistic function. Regression coefficients were estimated using bootstrapping (1000 iterations, sampling 15000 trials in each iteration

and then fitting the model). Figure 2D depicts the values of the w_n for each of the phase bins.

Next, effective pulse counts could be calculated by computing the dot product between the phase histogram (i.e. a vector indicating how many pulses occurred at each of the 15 phase bins) and the vector of weights plotted in FIgure 2D.

There are very few assumptions in our effective count calculation: (1) each breathing cycle can be divided into 15 bins independent of the cycle period – this is reasonable since mice are breathing quite steadily in our head-restrained task. (2) the perceptual weight of each bin is independent of the others – again, this is a reasonable assumption, and any dependency cannot be justified at present. Therefore, it is not clear how to calibrate and validate these methods or obtain sensitivity metrics.

Details regarding the specific parameters used in the deconvolution via the DUNL method would also benefit from a clearer presentation. Including these extra details will enhance transparency and help other researchers replicate the study.

We appreciate the Reviewer's point, and we agree that more details about how the deconvolution of APCx responses was conducted. We are including a more extensive explanation of how DUNL was fitted to the anterior piriform cortex population and the paragraph in the Methods section now reads (Lines 1024-1059):

Analysis of APCx neural responses using deconvolutional unrolled neural learning (DUNL): Single-trial neural activity was decomposed into impulse-like responses to odor pulse presentation as it has been described in a previous report (Tolooshams et al., 2024). *DUNL modelled the neural spikes with a Poisson distribution, whose rates in each trial were expressed as the convolution of a kernel characterizing the response of the neuron to an odor pulse with sparse codes (a vector representing the timing of events), together with the strength of the neural responses associated with those events, complemented by a baseline trial activity. DUNL used a bin size of 50 (i.e., for learning a 1s kernel at 1 ms resolution data, the kernel has 20 samples). Kernels were learned fully from data, and in this dataset a single kernel was learnt for each APCx neuron. DUNL has knowledge of the code support, i.e., non-zero elements of the sparse code were aligned to the timing of the odor pulses, and the encoder was unrolled for 100 iterations (with step size of 1) using ReLU activation functions to impose additional sparsity on the sparse codes. The baseline trial activity is computed using the average spike counts scaled by the number of occurred events (odors). Stochasticity in the estimated activity was added by passing the convolved signal through a generative model using a Poisson process. The kernel characterization is achieved per neuron in two stages: i) given the known timing*

(support), DUNL is trained with zero bias (no L1-based sparse) regularization; ii) DUNL is further fine-tuned with additional L1-regularization sparsity and a smoothness regularization on the kernel. We used smooth regularization of 0.1 for all neurons, and the L1 code regularization is fine-tuned based on the best fit (KS and R2) within the set [0, 0.005, …, 1, 2, 3, 4, 5]. This additional code sparsity is applied to account for the fact that neurons may not always respond to an odor. DUNL is trained with Adam optimizer (lr = 0.01, and eps = 0.001) where kernels are initialized from alignment of responses at the odor onset (regardless of overlap). For convenience, we are renaming the code amplitudes as 'response amplitudes' in Figure 4. Analysis on response amplitudes and kernels from APCx neurons was only performed in those neurons that were considered for the analysis of the spiking data. 'Phase clusters' were obtained by performing K-means clustering on the response amplitudes elicited by pulses at different times during respiratory phase in each neuron. 'Kernel clusters' were obtained after performing K-means clustering on the 1s kernels learned for each neuron.

There are also minor issues that, while not detracting from the overall strength of the study, could improve the clarity and presentation of the manuscript. For example, some typographical errors and minor editorial inconsistencies need to be corrected, and some sections could also benefit from a more organized layout. The inclusion of schematics or flow diagrams in the description of the analytical methods and in the presentation of the results would greatly facilitate the understanding of both the experimental procedures and the statistical analyses.

We appreciate the reviewer's point and we are including a schematic of the calculation of the effective odor pulse count (Supplementary Figure 3C) in this resubmission, since it was one of the issues the Reviewer considered unclear. We have also carefully combed through the manuscript for typos and stylistic inconsistencies, and corrected them all.

In summary, the findings of the manuscript are novel and highly relevant and have the potential to significantly influence the field of olfactory neuroscience. However, the current version has several aspects that require clarification and refining to guarantee that its conclusions are solid and reproducible. Given the potentially transformative nature of this work, I recommend a major revision to address these critical points, thereby strengthening the study's foundation and ensuring that its contributions are fully understood and replicable by the scientific community.

We appreciate the reviewer's valuation of our work, and we hope the reviewer finds that the new version of the manuscript provides a better layout and more clarification of the methodologies used in the analysis.

Reviewer 3

The manuscript by Boero and colleagues uses a wide array of experimental and theoretical approaches to investigate how the olfactory system in mice transforms intermittent odor stimuli into neuronal codes along the olfactory system and how it correlates with behavior. In particular, they address how the olfactory system responds to repeated stimuli during longer time windows, how this depends on the sampling, or breathing, cycle and how olfactory sensory neurons and neurons in the anterior piriform cortex deferentially represent such stimuli. Their approach is sophisticated, their results interesting, novel and informative and deepen our understanding of sensory coding within the olfactory system and also in comparison the other sensory modalities.

We thank the reviewer for these positive comments.

1. One issue the authors, somewhat briefly only mention in the discussion, is the question of the breathing frequency range their mice operate in. Guesstimating from the few sniff traces in their figures, the mice sniff around 4 Hz, which is only around 1/3 of their maximal, often used when exploring an olfactory environment, sniff frequency. They attribute this to the olfactory stimuli not being novel, but their head fixation also contributes to the lowered breathing rate. While this is unavoidable, the authors should discuss the implications. In particular, how their "long" inhalation phase (in comparison to a 10-12 Hz sniff rate) will affect not "missing" their odor stimuli, basically if they are over-counting the stimuli mice are perceiving. Alternatively, the mice might also learn (more on this issue below) to match their breathing rate to the task to improve their performance? The authors should state the breathing frequencies and limit their conclusion to that range.

We thank the reviewer for these insightful points, and we agree that our conclusions should be limited to the breathing frequency ranges observed during the task. In the current resubmission we are including two additional figures depicting the change in the dominant breathing frequency over the course of training, and a spectrogram of the breathing frequency for all trials of animals performing in the testing condition (New Supplementary Figures 2A and 2B, respectively). Overall, the new figures show that the dominant breathing frequency ranged from 2.5 to 5 Hz from the initial phases of task learning to testing. The observed respiratory frequencies are in agreement with previous

reports also using head-restrained mice, but they are significantly lower than the 4-12 Hz range displayed by mice when performing on freely-moving tasks requiring olfactory exploration (Wachowiak, 2011; Wesson, Donahou, et al., 2008). Therefore, as the reviewer correctly pointed out, the restricted range of breathing frequencies observed is likely to arise from the head-fixation rather than the lack of odor novelty. Instead, we believe that the latter condition could be underlying the absence of dynamic changes in the breathing frequency of trained animals during the sampling window (New Supplementary Figure 2B).

Regarding the possibility of mice learning an optimal breathing rate: New Supplementary Figure 2A shows that the dominant breathing frequency slightly decreases over the course of training. It is impossible for us to disentangle whether this is a gradual effect of head-fixation, a consequence of habituation to the odor stimuli, or - as the reviewer suggested - a result of mice learning to match their breathing frequency to some approximation of the rates at which pulses were delivered.

Lastly, even though breathing rates were low in the current experimental setup, the relative duration of the inhalation phase within the entire respiratory cycle was relatively constant across breaths (as can be seen in Figure 2A and Figure 3A). When phase histograms were computed for fitting the logistic regression of Figure 2D, bin lengths were warped so each phase bin represented the same phase of the respiratory cycle across breaths with different durations. Therefore, it is unlikely that our analysis could be overcounting the influence of pulses arriving during inhalation. In fact, by comparing the performance of animals on trials with similar pulse counts but different proportions of pulses falling in the inhalation phase (Figure 2E), we empirically showed the perceptual effect of breathing over pulses falling on inhalation without resorting to any statistical model. Nonetheless, we agree with the Reviewer that conclusions from our work should be limited to the current task -and for this range of breathing frequencies, since we do not know how OSNs would respond to intermittent stimuli when mice have bouts of high-frequency breathing, as they do during exploration or trail tracking.

Based on this, we decided to clarify these points in Results, which now reads (Lines 185-191):

The sampling of olfactory inputs by mice is tied to the airflow driven by inhalation and exhalation during breathing, or its active variant, sniffing. <u>While there was a slight trend towards lower breathing rate over the course of task learning, (Supplementary Fig. 2A), trained mice showed a stable breathing rate of ~3 Hz that was not affected by odor pulse presentation (Supplementary Fig. 2B). Therefore</u>, the amount of odor molecules inhaled from each pulse will depend on the arrival time of a given odor pulse relative to the breathing cycle.

And also in the Discussion, which now reads (Lines 680-694):

We would like to note that there are some caveats that may have to be considered before generalizing the scope of these results. First, similar to previous work using head-fixed behavioral setups, mice displayed a limited range of respiratory frequencies while performing the task (mostly 2.5-5 Hz, Supplementary Fig. 2A-B). The current bandwidth is in the lower end of the range displayed during active exploration or trail following (Jayakumar et al., 2025; Khan et al., 2012; Wachowiak, 2011)*. In addition, trained animals did not show any variations in the breathing frequency upon odor pulse presentation (Supplementary Fig. 2B) probably as a result of familiarization with the odorant. This contrasts with previous results revealing dynamic changes in breathing after presentation of novel odors*(Esquivelzeta Rabell et al., 2017; Wesson, Carey, et al., 2008)*. Finally, the decrease over learning in the dominant frequency of breathing prevents us from ruling out the possibility that mice learn to adjust their breathing frequency to the odor pulse delivery rates they experienced. Altogether, it is unclear whether the dependence of perceptual weighing (or OSN activity levels) of intermittent stimuli with the breathing cycle would be similar during high-frequency breathing, or the sampling of novel or unexpected odor cues.*

2. Fig. 5G: So do the authors believe that APCx acts as a low pass filter? Given that cluster 3 is the largest?

We thank the Reviewer for asking for a functional interpretation of the relative sizes of the phase clusters in the APCx. The reviewer is correct in pointing out that Cluster 3 is the largest: this means that most neurons in APCx responded with similar 'response amplitude' (instantaneous change in firing rate triggered by an odor pulse detection) to odor inputs arriving at any time within the breathing cycle. Since response amplitudes in Figure 5H were normalized for the peak response of each neuron, there was a possibility that the distribution of the absolute response amplitudes was different between clusters, but Supplementary Figure 5F clearly shows that is not the case. The lack of phase tuning in Cluster 3 neurons suggest that they not only become active for inputs arriving on the preferred phase of the other clusters but also respond to inputs happening outside the preferred breathing phase of other clusters. We are uncertain about how the phase invariant responses (cluster 3) relate to low (or high) pass filtering. The responses of neurons to odor pulses are still transient and time-locked to the odor pulse onset, but cluster 3 neurons just happen to have approximately the same amplitude independent of which breathing phase the pulse falls in. This is illustrated in the cartoon below where we show how the response of a typical neuron in cluster 1 or 3 will respond to pulses falling on 2 different breathing phases. Therefore, it has more to do with how the odor pulse timing with respect to the breathing phase affects aPCx response. The integration over time is related to the kernel shape, which is transient for most neurons (Supplementary Figure 5D).

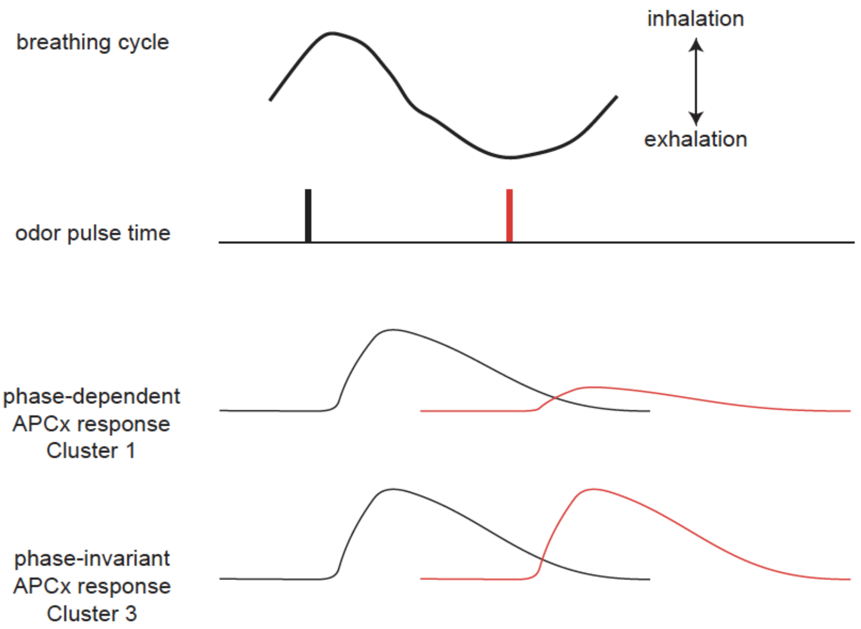

3. It might be worth considering adding Suppl Fig. 5 to the main Fig. 5 given its relevancy.

We appreciate the Reviewer's suggestion, and we now include the three plots in previous Supplementary Figure 5 at the end of the main Figure 5 (New Figure 5J, K, L).

4. How does the training to learn the task affect the encoding in the APCx? Are the observed 4 clusters a consequence of learning? Have the authors recorded from naïve mice using the same stimulus protocol?

We thank the reviewer for this insightful question. Unfortunately, we only collected electrophysiological recordings from the APCx neurons once animals were performing under the experimental conditions used for building the psychometric curve in Figure 1D (a 5s sampling window, $\lambda_1 = 12$, $\lambda_2 = 4$). We do not have recordings of APCx neurons during the training phase of the task. Therefore, we cannot properly answer if the 4 clusters are innately present in APCx or whether they are a consequence of task learning. We agree that it would be important in future experiments to acquire data from APCx neurons across all the learning process to investigate any temporal or learning-dependent changes in the tuning or response properties of APCx neurons.

Based on this question, we decided to add a brief comment in the Discussion section. Now lines 739-751 read:

What are the signals that shape the phase-tuned odor responses in APCx? One possibility is that a subpopulation of APCx neurons may inherit the tuning of their MTC inputs. Recurrent inhibitory signals in the APCx appear to be essential in controlling the timing and gain of APCx population responses to odors[59], and may contribute to phase tuning observed in our experiments. Moreover, a recent report has shown that, even in the absence of odors, inhalation can trigger bidirectional changes in the firing rates of APCx neurons, driven mostly by feedforward bulbar input, but with a small top-down component [27]. Mice decreased their dominant breathing frequency across task training (Supplementary Figure 2A). In this scenario, it becomes unclear if the phase clusters observed are innately present in the APCx population or if they emerge over the course of learning. Future experiments can test these alternatives, as well as assessing whether the functional differences observed across clusters are correlated with differences in molecular identity or connectivity of APCx neurons.

Minor comments:

Line 77: Delete one "how"

We thank the reviewer for pointing out this mistake. Now Lines 78-79 read:

Extensive prior work has characterized how sniffing modulates the basal activity and the time course of neuronal responses in the olfactory pathway

Fig. 1E legend. Some of this text there (especially the conclusion) might be best moved to the main text?

We agree with the reviewer that the legend was too long. We realized this was a result of a mistake during text formatting before manuscript submission, since the preprint version has most of the caption as a separate paragraph at the end of the first section of Results. The current legend reads (Lines 133-136):

***E**) Mixed-effects logistic regression coefficients obtained after fitting the behavioral choices to the 'Null' and 'Full' models using odor information divided in 5 bins. Models were compared using a log-likelihood ratio test with the p-value of the comparison indicated. Data is expressed as mean ± CI 95%.*

The rest of the text that was formerly in the caption, now it is a full paragraph in the Results section (Lines 170-182)

Reviewer 4

Mice can be trained to discriminate odor pulses delivered during multi-second trials based solely on their number. This task required the subjects to integrate information over several seconds. Pulses that arrived during inspiration were more important for the decision process than those arriving during exhalation. The authors show, using calcium imaging, that pulse number can be represent at the first synapse of the olfactory system in the olfactory bulbs. Finally, the authors show, using extracellular recordings, that the piriform or olfactory cortex is unlikely to be the integrator of odor pulse information given the stochastic and transient nature of its responses to a grouping of odor pulses.

This work will be of interest to any investigator trying to understand how animals follow odor trails/plumes including the memorial processes involved. The work is derivative of earlier work by Gumaste et al., 2024 cited it the paper. But, to be fair, the lab head was also an early contributor to this line of investigation (e.g. Rokni et al., 2014).

We thank the reviewer for the thoughtful summary of the work, and for placing it in a larger context. We agree that the Gumaste work is highly relevant (and we cite it prominently), but we also feel that there are significant differences. First, we focused on the pulse rates rather than intermittency as Gumaste et al did, and our pulse widths are briefer than theirs. As pointed out by the reviewer below, it is unlikely intermittency was the defining feature for our mice. Therefore, our study adds important complementary evidence for pulse counting when discontinuous odor stimuli are sensed. Second, we were able to examine in detail the dependence of the sniff phase on both the sensory responses and the behavior – an entirely novel set of experiments not tackled by Gumaste et al. Third, we quantified glomerular signals are much higher spatial resolution than Gumaste et al, allowing characterization of individual glomerular responses. Finally, we obtained single-unit recordings from APCx, which allowed us to characterize brain responses to these pulsatile, intermittent stimuli – again, for the first time to our knowledge. Therefore, we feel that our study is not merely derivative of the earlier work, but adds significant new insights.

1. The use of a single odorant at an exceedingly high concentration---one unlikely ever to be found in nature---requires some justification. For example, such a high concentration would recruit low specificity glomeruli obscuring the bulbs “normal” response profile.

We agree with the Reviewer that the concentration used in the current work is likely to be above the ranges that can be found in nature. The calculation of the phase-dependence

of calcium responses in OSNs only considered odor pulses separated by 2 s from each other, isolating how OSNs responded to single, transient, high-concentration odor inputs. We acknowledge that the time-integrated odor concentration of the odor pulses in the current task may exceed what can be experienced by animals in natural environments. As a result, the number of glomeruli recruited by the odor pulses is probably greater than what would be activated by intermittent stimuli in nature. Nevertheless, it is not our intention to draw conclusions about the absolute number of glomeruli activated by odor pulses in the task. Rather, we want to emphasize that all glomeruli exhibited the same phase dependence in their responses (Supplementary Figure 4), indicating that this phenomenon relies on intrinsic properties of OSNs rather than response patterns attributable to differences in concentration sensitivity.

Based on that, we agreed to include a small comment in the last paragraph of the second section of Discussion (Lines 708-716):

We note that the current work likely used odorant concentrations above what can be found in natural environments (Wachowiak et al., 2025)*. Consequently, the number of glomeruli activated upon odor pulse presentation is probably higher than what a naturalistic intermittent plume would trigger. However, it is important to emphasize that all activated glomeruli shared the same phase-dependence in the response, irrespective of their concentration sensitivity (Supplementary Figure 4). Altogether, our results provide functional evidence of the integration of olfactory stimuli over time for odor plume categorization, although complementary experiments are still required to determine which aspect of odor plume dynamics glomeruli track and what the underlying neural mechanisms are.*

2. The use of identical pulse sizes also would seem highly artificial and thus deserves comment.

We appreciate the Reviewer's concern relative to the nature of the olfactory stimuli presented to animals for decision-making. As Supplementary Figure 1A shows, 50 ms odor pulses of 5% ethyl valerate led to PID signals that had a halfwidth ~40 ms. In isolation, these odor profiles may look artificial, but they can be an accurate representation of transient increases in odor concentration observed during exposure to distant odor cues (Celani et al., 2014; Murlis et al., 1992; Reddy et al., 2022; Riffell et al., 2014; Rigolli et al., 2022; Young et al., 2020). More importantly, as can be seen in the left panels of New Supplementary Figure 1A, since odor pulses arrived randomly during the sampling window, they could also come close in time producing sub-second intervals of high intermittency. Therefore, the odor pulses can be used as 'building-blocks' to stochastically deliver olfactory stimuli with different levels of intermittency, while also accounting for other properties of naturalistic odor signals as periods of blanks and 'whiffs' of variable duration.

We added a brief comment relative to this point in Discussion (lines 550-553):

Importantly, PID recordings confirmed that, when pulses happened close in time, odor signals could sum over time and lead to short intervals of higher intermittency and overall amplitude. Therefore, by building stochastic sequences of odor pulse delivery, our olfactometer could successfully recreate naturalistic odor profiles.

3. The authors make a lot out of the fact that pulses arriving during inspiration were more important for the decision process than those arriving during exhalation. But this might have a trivial explanation in that expired air does not travel the same path across the entirety of the mucosal sheet as inspired air. You would need to uncouple breathing from sniffing to get around this confound.

We agree with the Reviewer that the differences in behavioral weighing of odor pulse coming during inhalation or exhalation likely stem from the different trajectories the odorized air would go through in each case. While this may be expected, it was important to confirm that the olfactory system (or higher-processing centers in the brain) was not attempting to perceptually compensate those pulses that triggered weaker responses in the olfactory periphery. Therefore, a key finding of our study that the high degree of correlation between the behavioral weights and the calcium responses in OSNs, required us to measure the phase dependence of both variables. However, we appreciate the Reviewer's comment, and are incorporating that in the Discussion (Lines 655-668):

The brief nature of the odor pulses used in our task was essential for testing the effects of breathing in the representation of olfactory stimuli across the ascending olfactory pathway and its later weighing for behavior. Previous reports have shown that MTC responses to steady odor stimuli tile the respiratory phase with a bias towards inhalation (Fukunaga et al., 2012; Gupta et al., 2015; Shusterman et al., 2011). *Here, we show that OSN responses were larger for pulses arriving during inhalation than for pulses arriving during exhalation, suggesting that breathing-modulated odor responses are already present at the first relay of the ascending olfactory pathway. This can be logically explained if OSN activity is contingent on the amount of odor molecules carried by the nasal airflow during breathing. While this may be expected, the high-degree of correlation in the phase-dependence of OSN responses and the behavioral weights captured by the logistic regression analysis (FIgure 2D), suggests that decision-making centers in the brain do not compensate for weaker 'exhalatory' inputs, remaining limited by the constraints imposed by breathing.*

4. It's unclear whether or not the authors tested mice without odor in the operant chamber. Showing that the mice work at chance levels without odor is the only way

to be sure they are not using other cues besides odor, e.g. valve noise or vibrations or air movement changes, to discriminate S+ and S- trials (see Burt Slotnick's work for example).

We thank the Reviewer for this excellent point. We took their suggestion and trained a new cohort of animals (n=3) with catch trials (10% of total trials in the session) in which 50 ms pulses of clean air were delivered by the activation of a third valve. This clean air valve was a different valve and vial than the used for alternating with the odor pulses during normal trials. Pulse timing and counts in the catch trials were generated with the same parameters as for the regular odor pulses, and reward sites followed the same dependency based on total pulse counts. We observed that mice preferentially and overwhelmingly chose the 'Low' side for catch trials irrespective of the total number of clean air pulses presented (Supplementary Figure 1E), suggesting that mice interpreted them as 'Low' trials with almost null odor signal. Therefore, this confirmed that mice were actually using olfactory cues for making decisions in the task rather than relying on other non-olfactory signals.

We added a brief, but important, comment about this experiment in Results (Lines 163-169)

To test if mice were using pressure or airflow changes as cues for decision making, we introduced catch trials with varying number of clean air pulses (instead of the odorized air), which were rewarded by following the same rule as for the odor pulses. Catch trials were categorized by mice as 'Low' trials irrespective of the total pulse count (Supplementary Fig. 1E, grey traces), confirming that they were basing their decision on the integrated number of odor inputs rather than mechanosensory or auditory signals arising from any airflow transients or valve openings.

5. At least one example of the raw data from a mouse working to criterion in the operant task should be included if only in supplementary documents.

We agree with the Reviewer that this is an important point and we have currently added a plot showing the learning trajectory of an animal -from the first day of training to testing criterion- in Supplementary Figure 1B.

We also amended the training times initially described for the task that were only considering the training in the initial binary classification of odor pulses with the extreme lambdas. Shaping to the experimental conditions that we used for testing mouse behavior (sampling window: 5s, $\lambda_1 = 12$, $\lambda_2 = 4$) took on average 2-3 additional weeks depending on mice performance. We decided to be cautious and change experimental conditions gradually over shaping. In the future, it would be interesting to test if shaping phase could be reduced by conducting more abrupt changes in the curriculum, and whether those

changes affect the way in which odor information is integrated once mice reach the testing conditions.

Now, lines 877-892 of Methods section read:

Once animals showed commitment to lick until they were satiated, they started the actual training in the behavioral task. A summary of the stages of the training for an example animal is provided in Supplementary Fig. 1B. Initially, mice started in the 'Block training' phase: actual task structure was introduced, with odor pulses being delivered during a 1 s sampling window. At this point, the total number of pulses in a trial could be drawn from two different Poisson distributions with different mean values: λ_1=16 for 'High Trials' and λ_2=1 for 'Low Trials', with both types of trials presented in blocks of 17 trials. Also, for the first 1-2 sessions of 'Block Training', water was delivered automatically to the rewarded side to help the animal to learn the association between the number of pulses and the side of the reward. After the second session in this phase, water was only delivered upon the animal licking in the side associated with reward for that type of trial. Block size was progressively reduced after animals reached more than 80% success in a session until the two types of trials were delivered randomly. Then, the behavioral shaping phase started: keeping the random trial structure, the difference between λ_1 and λ_2 was progressively reduced as well as the sampling window duration was increased. The whole training process, including training in the initial binary classification and later shaping, took 4-6 weeks.

6. Some further interpretation of the data in Figure 1E seems warranted. For example, shouldn't we have expected the rules of primacy and recency to apply; not at all what you are seeing.

We thank the Reviewer for asking this insightful question. Primacy and recency effects in the integration of sensory evidence for decision-making -as well as uniform integration- have been observed across diverse behavioral tasks and species. It is still not entirely clear whether those effects arise as a consequence of task structure, biases in the individual subjects, expertise in the task, or a combination of all. Our results show that animals tend to give more weight to information arriving around the middle part of the trials for making binary choices in the task. Lower weights during the initial part of the sampling period seem to rule out the possibility of a primacy effect. This would also be coherent with the observation of leaky integrators during tasks involving sensory integration over several seconds. Disregarding a recency effect becomes more difficult, especially considering that it is unclear when the animal made the internal decision of the binary choice with respect to the end of the sampling period. It is possible that mice were committing to a decision earlier in the sampling period and giving relatively higher weight

to the sensory information arriving right before that moment. Since the animal already made a decision, sensory inputs coming after that point may have minimal effect on the decision, which could explain the lower weights towards the end of the sampling period. Lastly, it is important to consider that animals could also adapt their strategy based on the length of the sampling period.

Given the Reviewer's question, we considered appropriate to clarify this in the Discussion (lines 601-614):

The reduced weights for inputs coming at the beginning of the sampling period implies the absence of a primacy effect for decision-making, suggesting that the storage of early sensory information could be affected by leakage in the integrator, similar to what was previously shown for rats performing multisensory integration for binary choices (Raposo et al., 2012)*. On the contrary, the reduced weights towards the end of the sampling period could, in principle, indicate the lack of a recency effect. However, it is also possible that the decrease in perceptual weights by the end of the sampling period could be explained by animals already committing to a decision and then neglecting sensory inputs coming afterwards, as it has been proposed by recent work modelling neural dynamics during binary decision-making* (Esnaola□Acebes et al., 2022; Luo et al., 2025)*. In that scenario, animals could be reaching a decision commitment before the end of sampling period and the increased perceptual weights in the middle could be reflecting a recency effect relative to decision commitment. Future experiments with neural recordings from brain areas carrying explicit information about the accumulated olfactory evidence in the task could help to clarify this issue.*

7. Given that Gumaste et al., 2024 found sniffing to influence odor intermittency discrimination some justification for not measuring sniffing modulation in this work is appropriate. In any event, fast sniffing must have influenced the measurement of synchrony of inhalation/exhalation with pulse. Some further explanation about sniffing's effect on synchrony measurements is warranted.

We thank the Reviewer for asking about this point. In Gumaste et al., 2024, authors found the synchrony between odor presentation and sniffing (or breathing) could affect the intermittency perceived by animals. The authors also showed that 'perceived intermittency' would ultimately alter the timing of anticipatory licking, as well as the proportion of 'hits' and 'misses' in the behavioral task (Gumaste et al., 2024, Figure 3D-E). Despite differences in task structure, Figure 2E in our work shows that 'High' trials with more pulses arriving during exhalation - i.e. a lower 'effective count' in the terms of the current work or 'perceived intensity' in terms of Gumaste et al. - were associated with lower performances compared to trials in which pulses (same pulse counts) were

presented mostly during inhalation (higher 'effective count'). Interestingly, Figure 3-figure supplement 1 in Gumaste et al., shows that sniff frequencies were mostly constant across trials with different intermittencies, with the exception of intermittency 0.5 in the 'Square-wave' trials from which they only had data from one session. Sniffing traces in Gumaste et al. show brief intervals of subtle sniff modulation, but without an accurate quantification of them it is unclear how frequent those events were across trials.

We are including in the resubmission an analysis of the dominant breathing frequencies across task training (Supplementary Figure 2A), revealing a trend to decreasing breathing rate with learning. We also show a spectrogram built from breathing traces of 7 animals working in the testing condition (sampling window: 5s, λ_1 = 12, λ_2 = 4) depicting the lack of changes in breathing frequency throughout trials. We attribute the lack of breathing modulation in our current work to animals being head-fixed, a setting that has been shown to constrain their breathing rate- and familiarization with the odorant throughout learning. We cannot disregard that the perceptual effects of the synchronicity between breathing and odor inputs could be different in behavioral settings allowing a higher range of sniff modulation, or in presence of novel stimuli.

Based on this, we decided to clarify these points in Results, which now reads (Lines 185-191):

The sampling of olfactory inputs by mice is tied to the airflow driven by inhalation and exhalation during breathing, or its active variant, sniffing. While there was a slight trend towards lower breathing rate over the course of task learning and shaping (Supplementary Fig. 2A), trained mice showed a stable breathing rate of ~3 Hz that was not affected by odor pulse presentation (Supplementary Fig. 2B). Therefore, the amount of odor molecules inhaled from each pulse will depend on the arrival time of a given odor pulse relative to the breathing cycle.

And also in the Discussion, which now reads (Lines 680-694):

We would like to note that there are some caveats that may have to be considered before generalizing the scope of these results. First, similar to previous work using head-fixed behavioral setups, mice displayed a limited range of respiratory frequencies while they were performing in the task (mostly 2.5-5 Hz, Supplementary Fig. 2A-B). The current bandwidth is in the low-frequency end of the range displayed during active exploration or trail following (Jayakumar et al., 2025; Khan et al., 2012; Wachowiak, 2011)*. In addition, trained animals did not show any variations in the breathing frequency upon odor pulse presentation (Supplementary Fig. 2B) probably as a result of familiarization with the odorant. This contrasts with previous results revealing dynamic changes in breathing after presentation of novel odors* (Esquivelzeta Rabell et al., 2017; Wesson, Carey, et al., 2008)*. Finally, the decrease over learning in the dominant frequency of breathing prevents us from disregarding the possibility of mice learning to adjust their breathing frequency to*

the odor pulse delivery rates they experienced. Altogether, it is unclear whether the dependence of perceptual weighing (or OSN activity levels) of intermittent stimuli with the breathing cycle would be similar during high-frequency breathing, or the sampling of novel or unexpected breathing odor cues.

8. To be convincing, Supplementary Figure 1 should show a PIP sensor readout for multiple puffs including high frequency puffs.

We agree with the Reviewer and we modified Supplementary Figure 1A, which now displays a PID trace corresponding to a trial with multiple ethyl valerate odor pulses, together with a magnification of the signal for a single pulse. The figure also displays the same measurements for the 50 ms methyl butyrate odor pulses used for training the new cohort necessary for answering the following question from the Reviewer.

9. Justification for use of ethyl valerate as the lone odorant used in the study should be added.

We thank the Reviewer for this insightful point. We did not have a particular reason for choosing ethyl valerate over other monomolecular odors. We started by considering odors that we knew did not trigger innate aversive responses in mice. Additionally, we knew it could trigger responses in multiple glomeruli of the dorsal surface of the olfactory bulb. We acknowledge that many other different substances comply with those conditions and could have also been chosen.

To exclude the possibility that the effects reported in this paper were specific to mice integrating ethyl valerate odor pulses, we trained a new cohort of animals in the task (n=3), but using 50 ms of 5% (v/v) methyl butyrate odor pulses. In this resubmission we are including PID measurements of a trial with multiple methyl butyrate pulses, as well as a magnification of the response triggered by a single pulse (Supplementary Figure 1A, bottom). Animals could be successfully trained in the task and the psychometric curve for trials working in the same testing criterion used for the rest of the article (sampling window: 5s, λ_1 = 12, λ_2 = 4) can be found in Supplementary Figure 1E.

Therefore, these additional results confirmed that the behavioral findings initially obtained with the ethyl valerate pulses can be generalized to other odor stimuli. We added a mention to this point in the Results section (Lines 159-163):

Learning or performance in the task did not rely on the integration of a property specific to ethyl valerate since a separate cohort of animals (n=3) could be trained using 50 ms odor pulses (see Supplementary Fig. 1A, bottom, for the PID traces) of 5% methyl

butyrate and reach similar performance in the testing condition (Supplementary Fig. 1E, green traces).

References

Celani, A., Villermaux, E., & Vergassola, M. (2014). Odor landscapes in turbulent environments. *Physical Review X*, *4*(4), 041015. https://doi.org/10.1103/PhysRevX.4.041015

Davis, H., & Pérusse, R. (1988). Numerical competence in animals: Definitional issues, current evidence, and a new research agenda. *Behavioral and Brain Sciences*, *11*(4), 561–579. https://doi.org/10.1017/S0140525X00053437

Esnaola-Acebes, J. M., Roxin, A., & Wimmer, K. (2022). Flexible integration of continuous sensory evidence in perceptual estimation tasks. *Proceedings of the National Academy of Sciences of the United States of America*, *119*(45), e2214441119. https://doi.org/10.1073/pnas.2214441119

Esquivelzeta Rabell, J., Mutlu, K., Noutel, J., Martin Del Olmo, P., & Haesler, S. (2017). Spontaneous rapid odor source localization behavior requires interhemispheric communication. *Current Biology*, *27*(10), 1542-1548.e4. https://doi.org/10.1016/j.cub.2017.04.027

Fukunaga, I., Berning, M., Kollo, M., Schmaltz, A., & Schaefer, A. T. (2012). Two distinct channels of olfactory bulb output. *Neuron*, *75*(2), 320–329. https://doi.org/10.1016/j.neuron.2012.05.017

Gallistel, C. R., & Gelman, I. (2000). Non-verbal numerical cognition: from reals to integers. *Trends in Cognitive Sciences*, *4*(2), 59–65. https://doi.org/10.1016/s1364-6613(99)01424-2

Gumaste, A., Baker, K. L., Izydorczak, M., True, A. C., Vasan, G., Crimaldi, J. P., & Verhagen, J. (2024). Behavioral discrimination and olfactory bulb encoding of odor plume intermittency. *ELife*, *13*. https://doi.org/10.7554/eLife.85303

Gupta, P., Albeanu, D. F., & Bhalla, U. S. (2015). Olfactory bulb coding of odors, mixtures and sniffs is a linear sum of odor time profiles. *Nature Neuroscience*, *18*(2), 272–281. https://doi.org/10.1038/nn.3913

Jayakumar, S., Rigolli, N., Mathis, M. W., Vergassola, M., Mathis, A., & Murthy, V. N. (2025). Mice navigate scent trails using predictive policies. *BioRxiv*.

Kaufman, E. L., Lord, M. W., Reese, T. W., & Volkmann, J. (1949). The discrimination of visual number. *The American Journal of Psychology*, *62*(4), 498. https://doi.org/10.2307/1418556

Khan, A. G., Sarangi, M., & Bhalla, U. S. (2012). Rats track odour trails accurately using a multi-layered strategy with near-optimal sampling. *Nature Communications*, *3*, 703. https://doi.org/10.1038/ncomms1712

Luo, T. Z., Kim, T. D., Gupta, D., Bondy, A. G., Kopec, C. D., Elliot, V. A., DePasquale, B., & Brody, C. D. (2025). Transitions in dynamical regime and neural mode underlie perceptual decision-making. *BioRxiv*. https://doi.org/10.1101/2023.10.15.562427

Mahajan, S., Tamboli, S., Das, S., Bhattacharjee, A. S., Pardasani, M., Srikanth, P., Marathe, S. D., Adlakha, A., Ranjan, L., Pandey, S., & Abraham, N. M. (2024). Mouse olfactory system acts as anemo-detector and -discriminator. *BioRxiv*.

Murlis, J., Elkinton, J. S., & Cardé, R. T. (1992). Odor Plumes and How Insects Use Them. *Annual Review of Entomology*, *37*(1), 505–532. https://doi.org/10.1146/annurev.en.37.010192.002445

Pnevmatikakis, E. A., & Giovannucci, A. (2017). NoRMCorre: An online algorithm for piecewise rigid motion correction of calcium imaging data. *Journal of Neuroscience Methods*, *291*, 83–94. https://doi.org/10.1016/j.jneumeth.2017.07.031

Pnevmatikakis, E. A., Soudry, D., Gao, Y., Machado, T. A., Merel, J., Pfau, D., Reardon, T., Mu, Y., Lacefield, C., Yang, W., Ahrens, M., Bruno, R., Jessell, T. M., Peterka, D. S., Yuste, R., & Paninski, L. (2016). Simultaneous denoising, deconvolution, and demixing of calcium imaging data. *Neuron*, *89*(2), 285–299. https://doi.org/10.1016/j.neuron.2015.11.037

Raposo, D., Sheppard, J. P., Schrater, P. R., & Churchland, A. K. (2012). Multisensory decision-making in rats and humans. *The Journal of Neuroscience*, *32*(11), 3726–3735. https://doi.org/10.1523/JNEUROSCI.4998-11.2012

Reddy, G., Murthy, V. N., & Vergassola, M. (2022). Olfactory sensing and navigation in turbulent environments. *Annual Review of Condensed Matter Physics*, *13*(1). https://doi.org/10.1146/annurev-conmatphys-031720-032754

Riffell, J. A., Shlizerman, E., Sanders, E., Abrell, L., Medina, B., Hinterwirth, A. J., & Kutz, J. N. (2014). Sensory biology. Flower discrimination by pollinators in a dynamic chemical environment. *Science*, *344*(6191), 1515–1518. https://doi.org/10.1126/science.1251041

Rigolli, N., Magnoli, N., Rosasco, L., & Seminara, A. (2022). Learning to predict target location with turbulent odor plumes. *ELife*, *11*. https://doi.org/10.7554/eLife.72196

Scott, B. B., Constantinople, C. M., Erlich, J. C., Tank, D. W., & Brody, C. D. (2015). Sources of noise during accumulation of evidence in unrestrained and voluntarily head-restrained rats. *ELife*, *4*, e11308. https://doi.org/10.7554/eLife.11308

Shusterman, R., Smear, M. C., Koulakov, A. A., & Rinberg, D. (2011). Precise olfactory responses tile the sniff cycle. *Nature Neuroscience*, *14*(8), 1039–1044. https://doi.org/10.1038/nn.2877

Tolooshams, B., Matias, S., Wu, H., Temereanca, S., Uchida, N., Murthy, V. N., Masset, P., & Ba, D. (2024). Interpretable deep learning for deconvolutional analysis of neural signals. *BioRxiv*, *Accepted at Neuron*. https://doi.org/10.1101/2024.01.05.574379

von Glasersfeld, E. (1982). Subitizing: The role of figural patterns in the development of numerical concepts. *Archives de Psychologie*, *50*(194), 191.

Wachowiak, M., Dewan, A., Bozza, T., O’Connell, T. F., & Hong, E. J. (2025). Recalibrating olfactory neuroscience to the range of naturally occurring odor

concentrations. *The Journal of Neuroscience*, *45*(10). https://doi.org/10.1523/JNEUROSCI.1872-24.2024

Wachowiak, M. (2011). All in a sniff: olfaction as a model for active sensing. *Neuron*, *71*(6), 962–973. https://doi.org/10.1016/j.neuron.2011.08.030

Wesson, D. W., Carey, R. M., Verhagen, J. V., & Wachowiak, M. (2008). Rapid encoding and perception of novel odors in the rat. *PLoS Biology*, *6*(4), e82. https://doi.org/10.1371/journal.pbio.0060082

Wesson, D. W., Donahou, T. N., Johnson, M. O., & Wachowiak, M. (2008). Sniffing behavior of mice during performance in odor-guided tasks. *Chemical Senses*, *33*(7), 581–596. https://doi.org/10.1093/chemse/bjn029

Young, B. D., Escalon, J. A., & Mathew, D. (2020). Odors: from chemical structures to gaseous plumes. *Neuroscience and Biobehavioral Reviews*, *111*, 19–29. https://doi.org/10.1016/j.neubiorev.2020.01.009

Response to Reviewers

There were no additional concerns or questions from the reviewers – therefore, no response is necessary. We are grateful for the helpful and thorough review process.

REVIEWERS' COMMENTS

Reviewer #2 (Remarks to the Author):

After evaluating the authors' responses and the revised manuscript, I am satisfied that the main concerns raised in the previous review round have been fully addressed.
The authors now provide a clear and well-supported rationale for the absence of evidence-accumulation signals in the piriform cortex, placing their findings appropriately within the broader literature on sensory decision-making. Their response convincingly explains why the lack of ramping activity is unlikely to result from methodological limitations and clarifies that their conclusions apply specifically to the present task.
The expanded discussion on alternative decision strategies, as well as the justification of the modeling assumptions, also strengthens the conceptual framework of the study.
Importantly, the authors have substantially improved methodological transparency by providing detailed explanations of the effective pulse calculation and the DUNL deconvolution procedure, which now meet standards for reproducibility.
Minor issues of clarity and presentation have been corrected, and the inclusion of additional schematics helps visualize key analytical steps.
Overall, the manuscript presents novel and rigorous work that will be of significance to the field. The revisions adequately support the main conclusions.
I recommend the manuscript for publication

Reviewer #3 (Remarks to the Author):

The manuscript by Boero and colleagues uses a wide array of experimental and theoretical approaches to investigate how the olfactory system in mice transforms intermittent odor stimuli into neuronal codes along the olfactory system and how it correlates with behavior. In particular, they address how the olfactory system responds to repeated stimuli during longer time windows, how this depends on the sampling, or breathing, cycle and how olfactory sensory neurons and neurons in the anterior piriform cortex differentially represent such stimuli. Their approach is sophisticated, their results interesting, novel and informative and deepen our understanding of sensory coding

within the olfactory system and also in comparison the other sensory modalities.

In their revised manuscript, the authors added new analysis to address questions raised and also discussed them appropriately and expertly in the discussion. I would like to thank them for the added revisions to improve their manuscript. I have no further concerns.

Reviewer #4 (Remarks to the Author):

All of my comments on the original draft have been thoroughly dealt with in the resubmission.
